# Liver cirrhosis in 2021: Global Burden of Disease study

**DOI:** 10.1371/journal.pone.0328493

**Published:** 2025-07-18

**Authors:** Hong Duo, Jian You, Siqi Du, Mengran Yu, Shaomei Wu, Pengpeng Yue, Xiao Cui, Yihui Huang, Jun Luo, Huaqin Pan, Qifa Ye

**Affiliations:** 1 National Quality Control Center for Donated Organ Procurement, Hubei Key Laboratory of Medical Technology on Transplantation, Hubei Clinical Research Center for Natural Polymer Biological Liver, Hubei Engineering Center of Natural Polymer-Based Medical Materials, Zhongnan Hospital of Wuhan University, Institute of Hepatobiliary Diseases of Wuhan University, Transplant Center of Wuhan University, Wuhan, Hubei, China; 2 Department of Gastroenterology, Renmin Hospital of Wuhan University, Wuhan, Hubei, China; 3 Transplant Intensive Care Unit, Department of Critical Care Medicine, Zhongnan Hospital of Wuhan University, Institute of Hepatobiliary Diseases of Wuhan University, Transplant Center of Wuhan University, Hubei Key Laboratory of Medical Technology on Transplantation; Clinical Research Center of Hubei Critical Care Medicine, Wuhan, China; Instituto Nacional de Ciencias Medicas y Nutricion Salvador Zubiran, MEXICO

## Abstract

**Background:**

Liver cirrhosis is a chronic progressive disease caused by various liver injury mechanisms, characterized by irreversible fibrosis, hepatocyte degeneration and necrosis, and the formation of regenerative nodules, ultimately leading to liver failure and multiple severe complications, significantly increasing the risk of mortality.

**Methods:**

This study analyzes global and China-specific trends in liver cirrhosis incidence, prevalence, mortality, and disability-adjusted life years (DALYs) from 1990 to 2021, focusing on various causes including hepatitis B virus (HBV), hepatitis C virus (HCV), alcoholic liver disease, and non-alcoholic fatty liver disease (NAFLD). Data from the Global Burden of Disease (GBD) database were used to assess changes in liver cirrhosis statistics globally and in China. The study examined incidence, prevalence, mortality, and DALYs over the period from 1990 to 2021.

**Results:**

Global liver cirrhosis incidence reached 58.4 million in 2021, up from 36.9 million in 1990, primarily driven by NAFLD, which increased from 24.8 million in 1990 to 48.3 million in 2021. While HBV- and HCV-related cirrhosis declined, deaths from alcoholic cirrhosis rose. In China, NAFLD became the main cause, although alcohol-related cirrhosis and an aging population remain major challenges.

**Conclusions:**

The growth of NAFLD and alcohol-related cirrhosis undermines progress in controlling HBV and HCV. Targeted prevention and management strategies are needed, especially in regions with low and middle SDI, which show higher cirrhosis mortality. For China, early intervention for NAFLD and continued control of HBV and HCV are critical to reduce the cirrhosis burden.

## Introduction

Liver cirrhosis is a chronic, progressive disease caused by various mechanisms of liver injury, characterized by irreversible fibrosis, hepatocyte degeneration, necrosis, and the formation of regenerative nodules in liver tissue. These pathological changes ultimately lead to liver failure and trigger several severe complications, such as ascites, esophageal variceal bleeding, and hepatic encephalopathy. These complications significantly increase the risk of mortality [1]. The etiology of liver cirrhosis is complex and multifactorial, primarily involving chronic infections with hepatitis B virus (HBV) and hepatitis C virus (HCV), alcoholic liver disease, and non-alcoholic fatty liver disease (NAFLD) [2–4]. The progression of liver cirrhosis typically occurs in two stages: compensated and decompensated. The compensated stage is often asymptomatic, during which the liver maintaining basic physiological functions. However, once the disease progresses to the decompensated stage, patients develop complications such as ascites, esophageal variceal bleeding, and hepatic encephalopathy. These complicationsnot only severely impair the quality of life but also significantly increase the risk of death [5]. Studies indicate that once decompensation occurs, the mortality risk for liver cirrhosis patients increases almost tenfold [2,6].

Globally, the incidence and mortality rates of liver cirrhosis show significant geographic and temporal variations. From 1990 to 2019, liver cirrhosis-related deaths increased from 10.21 million to 14.05 million, accounting for 2% of all deaths in 2019 [7]. Since 1980, the mortality rate of liver cirrhosis has decreased from 20 per 100,000 people to 15.8 in 2010 [8]. Although the mortality rate of chronic liver diseases caused by HBV and HCV has declined over the past three decades, total number of deaths continues to rise. From 1990 to 2019, the global burden of liver cirrhosis and other chronic liver diseases has evolved significantly. The primary cause of liver cirrhosis shifted from hepatitis B to hepatitis C during this period, while the impact of alcohol consumption increased markedly. Concurrently, the global incidence of liver cirrhosis in children and adolescents rose, with a decline in hepatitis B-related cirrhosis, while hepatitis C, NAFLD, and alcohol-related cirrhosis saw an increase. Nonetheless, the global disability-adjusted life year (DALY) rates for liver cirrhosis and other chronic liver diseases decreased [9–11]. The rapid growth in alcohol-related liver disease and NAFLD has, partially, offset the decline in the burden caused by viral hepatitis. Studies show that alcoholic liver disease contributes to 48% of all liver cirrhosis-related deaths, with approximately 15%–20% of individuals with chronic heavy alcohol consumption eventually developing cirrhosis [12,13]. NAFLD has become a major global health burden, with its incidence, mortality, and DALYs increasing between 1990–2019. China accounts for 23.6% of cases. Key risk factors include lifestyle factors, metabolic factors, smoking, and high fasting blood glucose. Early prevention and control of these factors is crucial [14]. The effects of liver cirrhosis and other chronic liver diseases vary notably based on geographical location, gender, race, ethnicity, and socioeconomic status, with these effects evolving significantly over time. This study primarily focuses on the incidence, prevalence, mortality, and DALYs of liver cirrhosis of various etiologies (including HBV-induced cirrhosis, HCV-induced cirrhosis, alcoholic cirrhosis, NAFLD, and cirrhosis due to other causes) worldwide and in China from 1990 to 2021, with age-standardized data. We conducted an in-depth analysis of the disease burden in both global and Chinese contexts. We provided detailed data summaries by region, gender, country, age group, and socio-demographic index (SDI). This study aims to offer the most comprehensive and up-to-date insights into the burden of liver cirrhosis and other chronic liver diseases globally and in China.

## Methods

The latest data from the Global Burden of Disease (GBD) database, covering up to 2021, includes 204 countries and regions, as well as five SDI levels, providing a comprehensive assessment of the impacts of 371 diseases, 88 risk factors, and injuries [15]. These valuable data can be accessed through the dedicated website managed by the Institute for Health Metrics and Evaluation at the University of Washington: https://vizhub.healthdata.org/gbd-results. This study primarily focuses on the incidence, prevalence, mortality, and DALYs for liver cirrhosis from 1990 to 2021, with age-standardized data processing. In particular, we conducted an in-depth analysis of the disease burden in both global and Chinese contexts, with detailed summaries of the data by region, gender, country, and different age groups. The design and reporting of this study adhered to both the STROCSS guidelines and the STROBE statement [16,17].

Countries and regions are categorized into five development levels based on SDI, and the world is geographically subdivided into 21 regions. The SDI is a composite measure that includes factors such as per capita income, average years of schooling, and fertility rates among young women, reflecting the overall development status of each country. The SDI values range from 0 to 1, divided into five categories: high (0.805129–1), high-middle (0.689504–0.805129), middle (0.607679–0.689504), low-middle (0.454743–0.607679), and low (0–0.454743) [18]. To accurately measure the trends in the incidence and mortality of liver cirrhosis and its types across different populations, this study employed age-standardized rates (ASR) and estimated annual percentage change (EAPC) [19,20]. ASR trends help capture changes in disease patterns across populations in greater detail, providing scientific evidence for developing targeted liver cirrhosis prevention strategies. The EAPC summary is used to quantify the ASR trends over a specific period across different populations, revealing dynamic changes in the disease burden. We calculated DALYs by combining years of life lived with disability and years of life lost, and standardized the results according to the global population data from the GBD. The final results are presented in terms of age-standardized DALY rates, incidence, prevalence, and mortality per 100,000 people, ensuring the international comparability and practical value of the data.

All data were obtained from the GBD 2021 database, and the estimation process was completed by the Institute for Health Metrics and Evaluation (IHME). Specifically, the IHME employed standard modeling tools, including the Cause of Death Ensemble Model (CODEm) and DisMod-MR 2.1, to estimate cause-specific mortality and nonfatal outcomes separately for combinations of sex, age, region, and year [21–23]. DisMod-MR 2.1 is a Bayesian meta-regression tool developed by IHME to integrate all available data on disease incidence, prevalence, remission rate, and mortality. CODEm is a systematic cause-of-death modeling method that combines covariate information and multiple data sources to generate the most robust model ensemble. Based on this modeling framework, IHME estimated the incidence, prevalence, mortality and DALYs of liver cirrhosis and its main causes (including hepatitis B, hepatitis C, alcohol, non-alcoholic fatty liver disease, and other causes), respectively, and provided 95% uncertainty intervals for all indicators to reflect the variability in the estimation process.

## Statistical analysis

This study extracted data on the incidence, prevalence, mortality, and DALYs of liver cirrhosis globally and in China from the Global Burden of Disease database. We calculated the corresponding age-standardized incidence rate (ASIR), age-standardized prevalence rate (ASPR), age-standardized mortality rate (ASMR), and age-standardized DALY rate (ASDR). The formula for age-standardized rates is as follows $\frac{\sum_{i=1}^{N}\alpha_{i}W_{i}}{\sum_{i=1}^{N}W_{i}}$, where ai represents the age-specific rate for the ith age group, and wi denotes the number of individuals (or the weighting) in the same age group within the GBD standard population [24,25]. The 95% uncertainty intervals (UIs) were defined as the 25th and 975th values from 1000 simulations. Additionally, we examined the correlation between health indicators related to liver cirrhosis and the SDI. A linear regression model based on the equation Y = α + βx + ε was used to assess the time trend, where Y is the natural logarithm of ASR, X represents the calendar year, and ε epsilon∊ is the error term. This model was used to determine the EAPC and its 95% confidence intervals (CIs). A smoothing spline model was employed to evaluate the relationship between the burden of liver cirrhosis-related diseases and SDI, covering 21 global regions and 204 countries and territories. The model integrates data on SDI and disease rates from different regions to calculate expected values. We used local weighted scatter plot smoothing to fit the model, which automatically adjusts the degree, number, and position of nodes based on the data and span parameters. To quantitatively analyze the correlation between age-standardized rates and SDI, Spearman’s rank correlation analysis was used to estimate the correlation coefficient (r value) and p-value. All data analysis and plotting were performed using R software (version 4.3.0), and a p-value less than 0.05 was considered statistically significant.

## Results

### Global and China incidence, prevalence, mortality, and DALYs

From 1990 to 2021, significant changes in the epidemiological indicators of liver cirrhosis occurred globally, reflecting the evolving patterns of different etiologies in terms of incidence, prevalence, mortality, and disease burden. In 2021, the global incidence of liver cirrhosis reached 58.4 million (95% UI: 54.2 million–62.8 million), showing a notable increase from 36.9 million in 1990. This rise is primarily attributed to NAFLD, whose incidence increased from 24.8 million in 1990 to 48.3 million in 2021, making it the leading cause of liver cirrhosis worldwide. In contrast, the incidence of HBV and HCV has significantly declined, with HBV decreasing from 7.7 million to 4.8 million, and HCV increasing from 3.7 million to 4.5 million. Although the incidence of alcohol-related liver cirrhosis has slightly decreased, its global burden remains substantial, with deaths rising from 223,000 in 1990–354,000 in 2021, underscoring the continued threat of alcohol consumption to public health. The global prevalence of liver cirrhosis also increased significantly during this period, rising from 988 million cases in 1990 to 1.7 billion in 2021, primarily driven by NAFLD, which accounted for 1.27 billion cases, far exceeding other etiologies. Despite declines in the prevalence of HBV and HCV, their disease burden remains high in certain regions, highlighting the need for continued efforts in hepatitis screening and treatment coverage. Globally, liver cirrhosis-related deaths increased from 1.02 million in 1990 to 1.43 million in 2021, indicating the ongoing health threat posed by this disease. Nevertheless, the ASMR decreased from 24.4 to 16.6. Meanwhile, global liver cirrhosis-related DALYs rose from 36.3 million in 1990 to 46.4 million in 2021.

Regarding trend changes, NAFLD’s ASIR had an EAPC of +0.73, making it the fastest-growing cause, reflecting the increasing global burden associated with rising obesity and metabolic syndrome. In contrast, the ASIR of HBV and HCV decreased by −2.74 and −0.51, respectively, reflecting the effectiveness of global efforts in antiviral treatment and vaccination. Although the incidence and mortality of alcohol-related liver cirrhosis have slightly declined, the global disease burden remains high. Overall, despite the declining trends in age-standardized mortality and disease burden, the global health burden of liver cirrhosis remains heavy due to the continued rise of NAFLD. In comparison with global trends, the epidemiological features of liver cirrhosis in China have shown similar changes, particularly concerning the influence of NAFLD and viral hepatitis. The incidence and prevalence of liver cirrhosis in China have significantly increased since 1990, with NAFLD emerging as the primary cause. In 2021, the incidence of liver cirrhosis in China was 10.99 million (95% UI: 10.12 million–11.86 million), showing an increase from 9.67 million in 1990, with a notable rise in NAFLD incidence from 6.18 million in 1990 to 9.56 million in 2021. Furthermore, the incidence of hepatitis B and C in China has declined, benefiting from the promotion of vaccination and antiviral treatment. However, despite the decrease in alcohol-related liver cirrhosis incidence, the health issues caused by alcohol consumption continue to pose a threat to public health in China. The death rate for liver cirrhosis in China also followed a similar trend to global patterns, decreasing from 179,200 in 1990–156,400 in 2021, reflecting the effectiveness of medical interventions and management. The ASMR in China decreased from 20.49 in 1990 to 7.69 in 2021, indicating improvements in mortality due to enhanced healthcare. However, liver cirrhosis-related DALYs in China remain high, at 4.5 million (95% UI: 3.55 million–5.56 million) in 2021. Although this is a decrease from 1990, the burden remains heavy due to the continued growth of NAFLD. The trends in liver cirrhosis in China align with global patterns, with NAFLD emerging as the main driver of future liver cirrhosis burden (S1-S2 Table).

### Heterogeneity analysis by etiology, region, and gender

From 1990 to 2021, the global burden of cirrhosis and its major etiologies exhibited complex and significant changes across different regions and SDI levels. During this period, the overall burden of cirrhosis continued to rise, while the incidence, prevalence, mortality, and DALYs were notably influenced by various factors such as etiology, gender, and regional differences. NAFLD gradually became the primary driver of the increasing cirrhosis burden, with both incidence and prevalence rising significantly across all SDI levels, particularly in high-income regions. This shift indicates that NAFLD has moved beyond being a personal health issue to becoming a major global public health challenge. In stark contrast, the burden of cirrhosis due to hepatitis B and C has significantly declined, particularly in high and upper-middle SDI regions. However, the decline in hepatitis B and C-related cirrhosis has been less pronounced in low-income and lower-middle-income regions. Alcohol-related cirrhosis presents a more complex trend worldwide. In high-SDI regions, both incidence and prevalence have slightly decreased, while in low-income regions, the burden of alcohol-related cirrhosis remains high, especially among men, highlighting the persistent public health threat posed by alcohol consumption. The burden of cirrhosis due to other causes, such as genetic diseases and autoimmune disorders, has slightly increased in high-income regions, possibly due to improved diagnostic rates and advances in medical technology, while remaining relatively stable in low-income regions. Overall, despite significant progress in the effective control of hepatitis B and C globally, the rapid rise of NAFLD and alcohol-related cirrhosis is offsetting these efforts. Particularly, the rise of NAFLD in high-income regions and the ongoing prevalence of alcohol-related diseases in low-income regions are presenting new health challenges. From a gender perspective, males exhibit significantly higher incidence, prevalence, and mortality across all etiologies, especially in alcohol-related liver disease and viral hepatitis, with gender differences being particularly pronounced. These differences reflect the influence of specific behaviors and health risks on disease burden. Regarding mortality, the number of deaths related to NAFLD has increased significantly, with a steady rise across all SDI levels, particularly in high-income regions. This increase is closely linked to the growing prevalence of obesity and metabolic syndrome. The rapid rise in the NAFLD-related mortality burden further underscores its status as a major global public health challenge. In contrast, mortality due to hepatitis B and C-related cirrhosis has significantly decreased, especially in high and middle SDI regions. However, this decline is less pronounced in low-income regions, where hepatitis B remains a major cause of death. Alcohol-related cirrhosis mortality displays regional variation: in high-income regions, the mortality rate has slightly decreased, likely due to policy interventions and alcohol consumption restrictions; while in low and lower-middle SDI regions, the mortality rate remains high, particularly affecting males, highlighting the long-term public health risks of alcohol. Additionally, mortality from cirrhosis due to other causes (e.g., genetic and autoimmune diseases) has slightly increased in high-SDI regions, with little change observed in low-SDI regions (S1-S4 Fig in S1 File).

### Trend for cirrhosis-related deaths globally (1990–2021)

**Fig 1:**
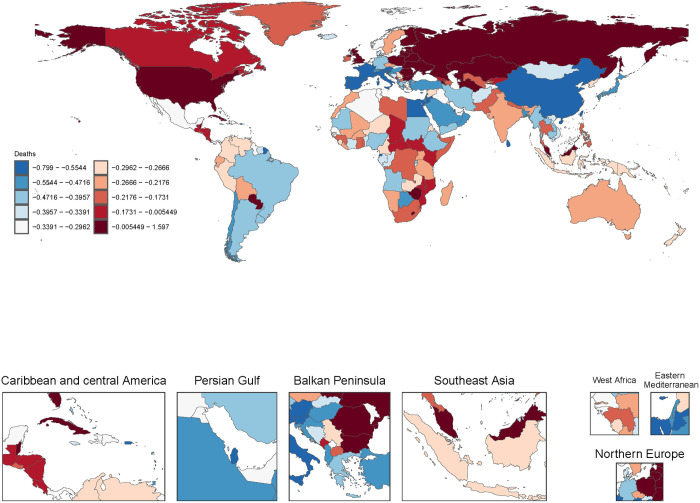
Global trends in percentage change of age-standardized deaths rates for cirrhosis, 1990–2021.

From 1990 to 2021, the age-standardized mortality rates (ASMR) for cirrhosis-related deaths showed varied trends across the globe. The ASMR for cirrhosis and other chronic liver diseases ranged from −0.799 to 1.597, with the most notable increases observed in Northern and Eastern Europe, while Sub-Saharan Africa and Southeast Asia showed a clear declining trend. For HBV, the ASMR ranged from −0.838 to 1.276, with significant decreases in Southeast Asia and Sub-Saharan Africa (−0.366 to −0.613), while Eastern Europe experienced an increase greater than 0.5. The ASMR for chronic HCV ranged from −0.823 to 1.628, with significant reductions in high-income regions such as North America (−0.418 to −0.823), while Central Asia saw an increase. The ASMR for alcohol-related cirrhosis ranged from −0.763 to 1.639, with marked increases in Northern and Eastern Europe, while Latin America and the Caribbean showed a decline (−0.5307 to −0.763). NAFLD saw the largest increase in ASMR, ranging from −0.704 to 2.831. This increase was closely linked to the rising prevalence of obesity and metabolic syndrome in high-income countries such as the United States and parts of Europe. Meanwhile, some regions of Latin America showed a moderate decline. The ASMR for cirrhosis due to other causes ranged from −0.785 to 1.262, with a moderate decline in Southeast Asia and Sub-Saharan Africa, possibly reflecting improvements in overall healthcare services (Fig 1 and S5-S8 Fig in S1 File). In 2021, the distribution of causes of cirrhosis-related deaths showed significant regional and gender-based differences. Chronic HBV was the leading cause of cirrhosis-related deaths in East Asia, Southeast Asia, and Sub-Saharan Africa, where it accounted for a substantial proportion of deaths. HCV contributed significantly to cirrhosis-related mortality in both Central Asia and Eastern Europe. Alcohol-related cirrhosis was most prominent in Eastern Europe, Central Asia, and Latin America, particularly among men. In contrast, NAFLD accounted for a notable proportion of cirrhosis-related deaths in high-income regions, such as North America, Western Europe, and high-income Asia-Pacific countries. However, its overall share remained relatively small. Gender differences were also evident, with a significantly higher proportion of male deaths attributed to alcohol-related cirrhosis, while women had a slightly higher proportion of deaths due to HBV, especially in Southeast Asia and Sub-Saharan Africa. Overall, viral hepatitis and alcohol-related cirrhosis remain the primary drivers of global cirrhosis mortality, with considerable differences in etiology across regions (S9-S12 Fig in S1 File).

### Gender and Age Differences in the Global Liver Disease Burden in 2021

**Fig 2:**
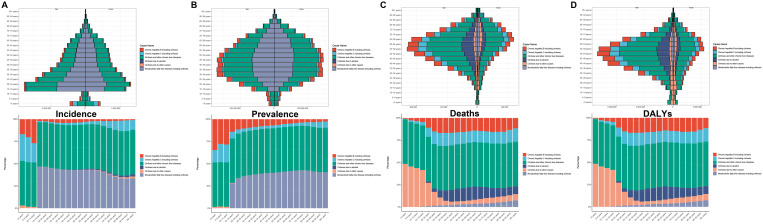
Global burden of disease for cirrhosis and chronic liver diseases: age and sex distribution of incidence, prevalence, mortality, and DALYs, 2021.

Global data reveal that the incidence of liver disease peaks in the young adult age group (20–34 years), with significantly higher rates in men compared to women. This elevated incidence is primarily driven by NAFLD and alcohol-related cirrhosis. As age increases, the gender disparity in incidence gradually narrows, becoming nearly equal in the elderly population. Regarding prevalence, liver disease prevalence increases significantly with age, with the highest prevalence observed in the middle-aged group (50–69 years). Men show a markedly higher prevalence than women, primarily due to the cumulative effects of chronic hepatitis B, hepatitis C, and alcohol-related cirrhosis. However, in the elderly population (70 years and older), the gender gap in prevalence diminishes, indicating that liver disease burden in this age group is more influenced by complications of end-stage diseases. Analysis of mortality rates shows that men have higher mortality rates across all age groups, particularly in the middle-aged group (45–59 years), where alcohol-related cirrhosis and chronic viral hepatitis are the leading causes of death. In the elderly population (75 years and above), mortality rates increase sharply, peaking in this age group. The analysis of DALYs further reveals that in 2021, men aged 15–54 had the most significant health loss burden, mainly driven by chronic hepatitis B, hepatitis C, and alcohol-related cirrhosis. However, after the age of 50, women’s DALYs rise significantly and approach those of men. In summary, results highlight that NAFLD and cirrhosis-related diseases dominate in terms of incidence, prevalence, mortality, and DALYs, playing a central role in the overall liver disease burden in 2021. Overall, liver disease burden displays significant gender and age-specific patterns, with young men and elderly women being key targets for intervention (Fig 2).

### Liver cirrhosis mortality by age in 1990 and 2021 in Global and China

Between 1990 and 2021, both the global and Chinese burdens of liver cirrhosis experienced significant changes. Overall, the incidence, prevalence, and mortality of liver cirrhosis gradually shifted from the middle-aged group (20−49 years) to the elderly group (≥60 years). In 2021, the incidence, prevalence, and mortality of liver cirrhosis in individuals aged 60 and above significantly increased compared to 1990, with the most notable rise observed in those aged 70 and above. This trend indicates that the elderly population has become the primary demographic group affected by liver cirrhosis, while the disease burden in younger individuals has decreased over this period. China’s trend mirrors the global pattern, though the impact of aging is more pronounced in China. In 2021, the incidence, mortality, and DALYs in individuals aged 65 and above were significantly higher than in 1990, with a particularly sharp increase in both prevalence and mortality rates in those aged 70 and above. At the same time, the incidence and mortality rates in the 20−49 age group decreased noticeably, suggesting that health interventions have made certain progress in reducing the disease burden among younger populations. However, compared to the decline in the burden among younger groups, the disease burden in the elderly population increased at a faster rate, leading to an overall rise in the liver cirrhosis burden. Changes in the number of cases and deaths indicate that, by 2021, the disease burden among middle-aged and young populations (20−49 years) in both the global and Chinese contexts had been alleviated. However, in China, the burden in individuals aged 65 and above exhibited a more significant upward trend than the global average. Particularly in terms of mortality rates, China’s growth in the 65 and above group was notably higher than the global average, suggesting that there is considerable room for improvement in disease management and healthcare resource allocation for this demographic. Overall, from 1990 to 2021, both globally and in China, liver cirrhosis burden displayed a significant aging trend. The increase in burden among the elderly population in China stands out, while the improvements in the younger population underscore the positive outcomes of health interventions (S13-14 Fig in S1 File). Results illustrate the long-term trends in the incidence, prevalence, deaths, and DALYs of liver cirrhosis and its major causes by sex in China from 1990 to 2021 (S15 Fig in S1 File). The results demonstrate that the burden of cirrhosis due to hepatitis B and C continued to decrease, while the burden of NAFLD increased significantly. Alcohol-related cirrhosis remained high or slightly increased in males. Across all causes, males generally exhibited higher mortality and DALY rates than females, particularly in cases related to viral hepatitis and alcohol consumption. S16 Fig in S1 File further shows the prevalence, incidence, mortality, and DALYs distribution of liver cirrhosis and its main causes in different age and gender groups in 1990 and 2021 in China. The results showed that the burden of various indicators in people aged 60 and above increased significantly in 2021 compared with 1990, especially in the age group of 70 and above. The burden caused by different causes also showed differences between genders: hepatitis B and alcoholic liver cirrhosis were dominated by males, while the proportion of liver cirrhosis caused by NAFLD was relatively high in females. Compared to 1990, the burden among young and middle-aged groups (particularly those aged 20–49 years) declined, whereas it increased notably among older adults, emphasizing a demographic shift in the distribution of cirrhosis burden over time.

### Global analysis of the mortality rate of cirrhosis and other chronic liver diseases and its association with the SDI

**Fig 3:**
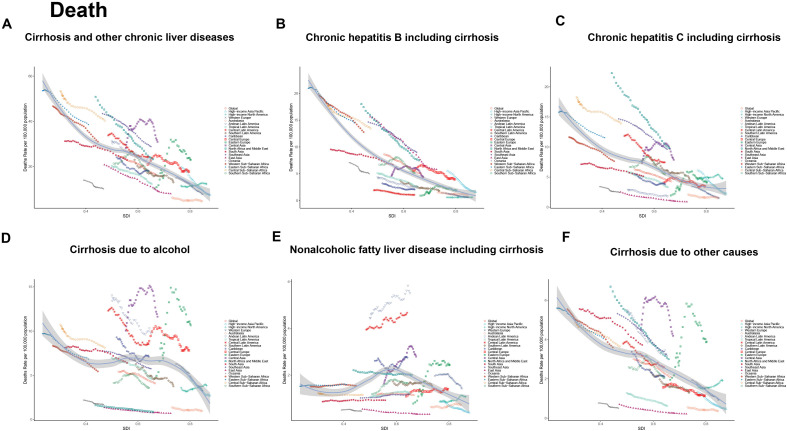
Age-standardized mortality rates of cirrhosis, globally and for 21 GBD regions, by SDI, 1990–2021.

In 2021, the global mortality rate from cirrhosis and other chronic liver diseases (per 100,000 population) exhibited a nonlinear inverse correlation with the SDI. Specifically, mortality rates in low-SDI and middle-SDI countries were significantly higher than in high-SDI countries. However, the distribution of different etiologies and their trends varied significantly across the SDI spectrum (Fig 3). Overall, mortality rates decreased with higher SDI, but significant regional differences associated with major etiologies were observed in low-SDI regions (e.g., Sub-Saharan Africa), middle-SDI regions (e.g., Eastern Europe and Central Asia), and high-SDI regions (e.g., North America and high-income Asia-Pacific). HBV-related mortality rates dominated in low-SDI regions, particularly in Sub-Saharan Africa, where mortality rates were significantly higher than the global average. This suggests that insufficient vaccination coverage and low antiviral treatment access are major drivers of the high cirrhosis burden in these areas. HCV-related mortality reached its peak in middle-SDI regions (e.g., Eastern Europe and Central Asia), reflecting the sustained impact of high infection rates and inadequate antiviral treatment coverage on disease burden. NAFLD-related mortality was primarily concentrated in high-SDI regions (e.g., North America, Western Europe, and high-income Asia-Pacific), where the burden of NAFLD significantly increased with higher SDI. This trend reflects the growing influence of obesity and metabolic syndrome on the cirrhosis burden in these areas. Alcohol-related cirrhosis mortality was notably higher in middle-SDI regions (e.g., Eastern Europe and parts of Latin America), indicating that alcohol consumption remains a major health challenge in these areas. Mortality from other causes (e.g., genetic disorders, autoimmune diseases) accounted for a smaller proportion globally but was relatively evenly distributed across different SDI regions. In summary, low-SDI regions, such as Sub-Saharan Africa, remain the core areas of the global cirrhosis burden due to high mortality rates related to HBV and HCV. Middle-SDI regions, such as Eastern Europe and Central Asia, continue to experience relatively high mortality due to alcohol consumption and HCV prevalence, while high-SDI regions, although having overall lower mortality, show a significant rise in NAFLD-related mortality, indicating that obesity and metabolic-related diseases have become major drivers of cirrhosis. In 2021, the global DALYs rate for cirrhosis showed a significant negative correlation with SDI. Low-SDI countries, such as the Central African Republic and Somalia, faced the heaviest burden, with DALYs exceeding 1,500 per 100,000 population, primarily due to a lack of medical resources and the prevalence of viral hepatitis. In contrast, high-SDI countries, such as Japan and Singapore, experienced a significantly reduced burden, with most countries reporting DALYs below 100 per 100,000 population, benefiting from well-established healthcare systems and vaccination programs. China, at a mid-to-high SDI level, exhibited DALYs close to the trendline, indicating recent progress in prevention and control. However, the burden among the elderly remains a prominent concern, and there is an urgent need to enhance the early prevention and management of chronic liver diseases. Future efforts should focus on resource optimization and targeted interventions to further reduce the burden of cirrhosis (S17-S22 Fig in S1 File).

### Trends in the burden of cirrhosis in China and worldwide

From 1990 to 2021, From 1990 to 2021, the global ASPR and DALYs for cirrhosis showed an overall decreasing trend an overall decreasing trend. Specifically, the burden of HBV-related cirrhosis significantly decreased, while HCV-related cirrhosis saw a slight decline. Conversely, the burden of cirrhosis associated with NAFLD continued to rise, and the burden from other causes remained relatively stable. The trends in China mirrored the global pattern, with a significant reduction in both the ASPR and DALYs for viral hepatitis-related cirrhosis, particularly for HBV-related cirrhosis, which saw a more pronounced decline. Similarly, HCV-related cirrhosis showed a slight decrease. However, the burden of NAFLD-related cirrhosis in China increased notably. The burden of cirrhosis from other causes remained stable, with minimal changes being observed (S23 Fig in S1 File).

## Discussion

Our research provides a comprehensive analysis of the global burden of cirrhosis and its major etiologies from 1990 to 2021, with a particular focus on global trends and the situation in China. By utilizing the latest data from the GBD study, we assessed the incidence, prevalence, mortality, and DALYs of cirrhosis across different regions, countries, periods, genders, and sociodemographic levels. The results offer valuable insights into the epidemiological transitions of cirrhosis and the inequalities driven by various causes, with important implications for public health strategies and can help guide the development of targeted cirrhosis prevention programs in different countries.

From 1990 to 2021, the global burden of cirrhosis underwent significant changes. Although the ASMR showed an overall decline, the total number of cases and deaths from cirrhosis increased because of population growth and aging. The global incidence of cirrhosis rose markedly from 36.92 million cases in 1990 to 58.41 million cases in 2021. This increase was primarily driven by the rapid rise of NAFLD, which, with the continued rise in obesity and metabolic syndrome, has become the leading cause of cirrhosis incidence and prevalence worldwide, particularly in high-income countries, where the number of cases has significantly increased [26–28]. Given the absence of approved pharmacological treatments for NAFLD, lifestyle interventions such as diet and exercise are considered the primary therapeutic approaches. Studies have shown that weight loss exceeding 10% may help reverse liver fibrosis in 45% of patients [29]. Moreover, first-degree relatives of NAFLD patients with advanced fibrosis are at 12.5 times the risk of developing advanced fibrosis compared to the general population, further highlighting the necessity of early identification and active intervention for patients [30]. In contrast, the burden of cirrhosis caused by HBV and HCV has significantly declined, thanks to global vaccination, antiviral treatments, and public health measures. By 2012, 183 countries had included the hepatitis B vaccine in their national immunization programs for infants, with 79% of children born in 2008 receiving all three doses of the hepatitis B vaccine. The Chinese government has prioritized HBV vaccination as a public health initiative [31,32]. This trend is particularly notable in regions such as East Asia and sub-Saharan Africa, where HBV is endemic. Alcoholic liver disease presents a more complex trend. Despite a slight decline in the global age-standardized mortality rate, the absolute number of deaths from alcoholic liver disease has increased, particularly in parts of Eastern Europe and Latin America. In these regions, individuals consuming more than 40 grams of pure alcohol daily are more than nine times more likely to develop cirrhosis than those who abstain from alcohol [33,34]. A large-scale prospective study in Mexico City found that compared to occasional drinkers, those consuming ≥210 grams of alcohol per week had a 43% higher all-cause mortality rate and nearly three times higher alcohol-related mortality. Liver disease mortality was particularly high among those drinking ≥140 grams per week, with a more than four times greater risk of death from liver disease compared to occasional drinkers [35]. Drinking patterns are also a significant factor in liver cirrhosis risk, with European countries primarily consuming spirits rather than wine or beer. Binge drinking or long-term daily alcohol consumption is strongly associated with an increased burden of alcoholic liver cirrhosis [36]. This indicates that harmful alcohol consumption continues to have a profound impact on public health. Therefore, countries should tailor their strategies based on local drinking habits, liver cirrhosis risk factors, and economic development levels. In particular, given the significantly high mortality rate from alcohol-induced liver cirrhosis, measures should be taken to limit excessive alcohol consumption and reduce the burden of alcoholic liver cirrhosis.

Trends in China generally mirror the global pattern, with NAFLD having become the main driver of the cirrhosis burden. From 1990 to 2021, cirrhosis cases related to NAFLD increased significantly in China, closely linked to the prevalence of obesity and sedentary lifestyles [37,38]. At the same time, the burden of HBV-related cirrhosis declined markedly due to the nationwide implementation of hepatitis B vaccination programs and the wider availability of antiviral treatments [39]. The incidence and prevalence of HCV-related cirrhosis also showed moderate declines, reflecting progress in HCV screening and treatment. Despite the progress in controlling HBV and HCV, alcoholic liver cirrhosis continues to be an ongoing issue in China. Although its incidence has slightly decreased, the health burden related to alcohol consumption remains significant. Additionally, the aging population in China has led to a shift in the burden of cirrhosis to older age groups, with an increasing proportion of cirrhosis cases and deaths occurring among those aged 65 and older.

The burden of cirrhosis varies significantly across regions and sociodemographic index (SDI) levels, reflecting differences in healthcare access, disease prevention, and lifestyle factors. In high-SDI regions such as North America and Western Europe, NAFLD-related cirrhosis dominates the disease burden due to the rising prevalence of obesity and metabolic syndrome. In contrast, in low-SDI regions such as sub-Saharan Africa, the burden of HBV-related cirrhosis remains high due to insufficient vaccination coverage and limited access to antiviral treatments [40]. Alcoholic liver disease is more prevalent in moderate-SDI regions such as Eastern Europe and parts of Latin America, where harmful drinking habits and inadequate policy interventions persist. NAFLD has rapidly become the leading cause of cirrhosis incidence and prevalence globally. The rise in NAFLD is closely linked to the global epidemics of obesity and metabolic syndrome, driven by unhealthy dietary patterns, urbanization, and sedentary lifestyles. Unlike HBV and HCV, NAFLD is closely related to socioeconomic development and primarily affects high- and middle-income countries. In China, the surge in NAFLD cases is closely associated with the country’s rapid economic growth, urbanization, and the adoption of Western dietary habits. The increasing prevalence of childhood and adolescent obesity in China signals a concerning trend, as early-onset metabolic diseases increase the risk of progressing to advanced liver disease later in life. These findings underscore the urgent need to prioritize NAFLD as a public health target, particularly in emerging economies undergoing rapid socioeconomic transitions, where urgent action is required.

Our findings highlight the need for region-specific and etiology-targeted strategies to mitigate the global burden of cirrhosis. For NAFLD, early identification and lifestyle interventions, including weight loss, dietary changes, and increased physical activity, are crucial for slowing disease progression. The development of pharmacological treatments for NAFLD should be a priority. For HBV, expanding vaccination coverage and improving access to antiviral treatments in low-SDI regions remain key tasks. Similarly, strategies to reduce transmission risks, such as needle exchange programs and antiviral treatments, should be strengthened to curb the spread of HCV. Alcoholic liver disease requires a comprehensive approach, including public health campaigns to reduce harmful alcohol consumption, strict alcohol policies, and improved healthcare services for high-risk populations. In China, the escalating burden of NAFLD-related cirrhosis urgently needs to be addressed. Public health campaigns promoting healthy lifestyles and weight management, as well as early screening programs for NAFLD, will help curb this trend. Moreover, continued efforts to control HBV and HCV through vaccination and antiviral treatments remain key to reducing the burden of viral hepatitis-related cirrhosis.

## Limitations

Although this study is based on GBD 2021 data and has broad coverage and considerable representativeness, there are still some limitations. First of all, GBD data are derived from primary sources of varying quality across countries, and the development level of health information systems differs between regions, which may lead to variations in data completeness and accuracy. Especially in low and middle SDI) regions, underreporting or misreporting of diseases is common, which may result in underestimation of the actual disease burden. Second, there are differences in the diagnosis, reporting, and data collection of liver cirrhosis and its causes across countries and regions, which may affect the comparability of global data. Third, this study used publicly available aggregate-level data, limiting in-depth analysis of potential influencing factors at the individual level (e.g., behavioral habits, environmental exposures, etc.). Therefore, future studies should strengthen quality control of data sources and combine individual-level case data to improve the accuracy and interpretability of estimates.

## Conclusion

In summary, the global burden of cirrhosis is undergoing profound changes, with NAFLD emerging as the leading cause of cirrhosis burden, and the burden of HBV and HCV-related cirrhosis is declining due to successful public health interventions. Alcoholic liver disease remains a significant issue in many regions. The findings underscore the need for region-specific prevention and management strategies tailored to different regions and SDI levels. For China, prioritizing NAFLD prevention and ongoing efforts to control HBV and HCV are crucial for reducing the long-term burden of cirrhosis.

## Supporting information

S1 Table Global burden of liver cirrhosis and its main etiologies in 1990 and 2021, and estimated annual percentage change (EAPC) from 1990 to 2021. (DOCX)

S2 Table China’s burden of liver cirrhosis and its main etiologies in 1990 and 2021, and estimated annual percentage change (EAPC) from 1990 to 2021. (DOCX)

S1 File This file contains Supporting Figures S1–S23. (PDF)
