# Supplementary material for: Liver cirrhosis in 2021: Global Burden of Disease study
Source: PLoS One. 2025 Jul 18;20(7):e0328493. doi: 10.1371/journal.pone.0328493 (PMC12273999; doi:10.1371/journal.pone.0328493)
Supplement: S1 Table — (DOCX) [file pone.0328493.s001.docx]

**Global**

| **Year** | **Cirrhosis** | **Hepatitis B** | **Hepatitis C** | **Alcohol** | **Other causes** | **NAFLD** |
| --- | --- | --- | --- | --- | --- | --- |
| **1990** |  |  |  |  |  |  |
| Incidence (95% UI) | 36922607.506(34219866.737-40051996.947) | 7746695.589  (6472836.109-9071842.770) | 3718033.693  (3088274.959-4416138.633) | 278939.603  229295.549-327575.216) | 337193.879  (290786.715-383918.813) | 24841744.741(22565085.702-27319936.665) |
| Prevalence (95% UI) | 988390222.578(919501408.059-1063915828.610) | 309391595.533 (282353914.311-340532265.530) | 109293618.793(89259257.434-131707609.271) | 1736365.483(1444016.615-2017537.618) | 3555323.534(3022838.859-4148935.488) | 564415673.975(516508605.551-618084432.530) |
| Death (95% UI) | 1021776.624(936628.902-1144617.860) | 359849.746(306103.995-423841.517) | 257010.289(214870.074-315977.351) | 223005.239(185494.577-263038.302) | 137050.070(112266.279-164272.017) | 44861.280(31679.790-61013.791) |
| DALYS (95% UI) | 36284496.065(33528220.598-40328345.030) | 12474269.958(10663158.256-14629527.090) | 8630240.340(7148331.317-10496175.210) | 7254802.222(6075464.137-8534060.167) | 6639942.029(5666015.959-7746660.094) | 1285241.516(895297.324-1775499.925) |
| ASIR (95% UI) | 691.292(645.489-745.210) | 134.578(113.244-156.395) | 68.547(56.686-81.136) | 6.636(5.485-7.817) | 6.350(5.437-7.273) | 475.180(432.271-517.764) |
| ASPR (95% UI) | 20088.744(18743.125-21508.361) | 5726.014(5234.404-6271.388) | 2174.776(1776.452-2606.107) | 40.226(33.502-46.631) | 63.100(53.522-73.591) | 12084.692(11058.040-13183.892) |
| ASMR (95% UI) | 24.424(22.375-27.484) | 8.602(7.288-10.108) | 6.261(5.234-7.648) | 5.398(4.517-6.388) | 3.004(2.419-3.694) | 1.158(0.810-1.574) |
| ASDR (95% UI) | 799.941(738.593-891.543) | 279.527(237.413-328.771) | 195.297(162.757-236.697) | 167.641(139.987-197.381) | 126.903(107.154-148.538) | 30.574(21.196-42.037) |
| **2021** |  |  |  |  |  |  |
| Incidence (95% UI) | 58417005.593(54231380.949-62796051.234) | 4767975.359 (4076108.469-5423253.826) | 4477374.428 (3691726.159-5356718.084) | 462685.917 (380016.824-543247.331) | 397988.918 (338934.710-461312.101) | 48310980.972(44191373.556-52313164.652) |
| Prevalence (95% UI) | 1697259271.732(1575345915.689-1823835811.837) | 283640016.232 (260114176.124-307691593.934) | 138559101.611(111487772.343-167350517.349) | 3015815.639(2502786.510-3480606.756) | 4229536.932(3549907.491-4956657.558) | 1267815566.469(1157880130.189-1380382443.091) |
| Death (95% UI) | 1425141.860(1308121.012-1563089.036) | 431964.393(365199.260-502419.327) | 368272.570(314030.728-427547.690) | 354253.949(299197.777-418947.379) | 173247.580(137379.363-214525.498) | 97403.367(69529.546-130168.313) |
| DALYS (95% UI) | 46417777.159(43056396.911-50687931.174) | 13882279.584(11749492.552-15998379.590) | 11822889.721(10109233.470-13698291.520) | 11146733.981(9444536.526-13296818.474) | 6894081.298(5716909.276-8275685.973) | 2671792.576(1895293.466-3602043.792) |
| ASIR (95% UI) | 724.306(672.982-779.555) | 61.506(52.439-69.808) | 59.367(48.902-70.741) | 5.338(4.404-6.253) | 5.311(4.561-6.078) | 592.784(542.226-643.236) |
| ASPR (95% UI) | 20302.624(18845.228-21791.911) | 3490.137(3197.573-3789.017) | 1704.785(1373.459-2061.699) | 34.811(28.882-40.257) | 55.442(46.652-64.851) | 15017.457(13755.839-16360.798) |
| ASMR (95% UI) | 16.640(15.283-18.262) | 8.602(7.288-10.108) | 4.294(3.672-4.980) | 4.084(3.452-4.813) | 2.092(1.674-2.563) | 1.139(0.818-1.518) |
| ASDR (95% UI) | 545.069(506.130-594.991) | 161.923(137.247-186.245) | 137.991(117.990-159.906) | 128.391(108.566-152.776) | 85.862(72.071-101.914) | 30.903(22.174-41.499) |
| **1990-2021** |  |  |  |  |  |  |
| ASIR (EAPC,  95% CI) | 0.12(0.08,0.16) | -2.74(-2.86,-2.62) | -0.51(-0.55,-0.48) | -0.78(-0.83,-0.73) | -0.69(-0.78,-0.59) | 0.73(0.69,0.77) |
| ASPR (EAPC,  95% CI) | 0.01(-0.03,0.05) | -1.69(-1.81,-1.58) | -0.85(-0.93,-0.77) | -0.53(-0.62,-0.45) | -0.49(-0.57,-0.40) | 0.73(0.67,0.79) |
| ASMR (EAPC,  95% CI) | -1.26(-1.35,-1.17) | -1.83(-1.93,-1.74) | -1.22(-1.29,-1.14) | -0.91(-1.03,-0.79) | -1.10(-1.22,-0.97) | -0.03(-0.10,0.04) |
| ASDR (EAPC,  95% CI) | -1.27(-1.38,-1.16) | -1.86(-1.96,-1.77) | -1.14(-1.23,-1.05) | -0.89(-1.03,-0.74) | -1.18(-1.34,-1.02) | 0.04(-0.06,0.13) |

DALYs disability-adjusted life-years, ASIR age-standardized incidence rate, ASPR age-standardized prevalence rate, ASMR age-standardized mortality rate, ASDR age-standardized DALYs rate, EAPC estimated annual percentage change, CI confdence interval, UI uncertainty intervals
