# Supplementary material for: Liver cirrhosis in 2021: Global Burden of Disease study
Source: PLoS One. 2025 Jul 18;20(7):e0328493. doi: 10.1371/journal.pone.0328493 (PMC12273999; doi:10.1371/journal.pone.0328493)
Supplement: S2 Table — (DOCX) [file pone.0328493.s002.docx]

**China**

| **Year** | **Cirrhosis** | **Hepatitis B** | **Hepatitis C** | **Alcohol** | **Other causes** | **NAFLD** |
| --- | --- | --- | --- | --- | --- | --- |
| **1990** |  |  |  |  |  |  |
| Incidence (95% UI) | 9664942.113(8925280.848-10538568.287) | 2803712.027(2360190.213-3225497.666) | 645186.121(530373.233-773476.494) | 14602.178(11133.781-18701.781) | 21691.644(17491.670-26396.523) | 6179750.144(5558026.279-6815755.457) |
| Prevalence (95% UI) | 291845382.228(271775839.953-315478805.488) | 127611666.257(115925328.108-140883473.970) | 26803824.861(21798633.263-32869400.758) | 116023.763(90226.334-146474.876) | 200402.660(152135.620-256945.138) | 137113464.687(125346813.251-151339952.882) |
| Death (95% UI) | 179245.638(151558.063-207693.301) | 137123.868(115512.442-158455.721) | 20747.227(16692.158-25755.198) | 10947.065(8329.338-13740.094) | 6204.364(4728.498-7906.649) | 4223.116(2786.391-5981.390) |
| DALYS (95% UI) | 6236551.204(5286217.071-7236713.314) | 4743312.288(3994645.263-5500372.236) | 713821.661(566034.587-892770.110) | 361175.566(272353.037-451622.191) | 289998.623(229909.938-369866.515) | 128243.066(83671.095-181154.214) |
| ASIR (95% UI) | 792.590(735.607-859.074) | 234.996(197.590-271.427) | 58.869(48.618-70.360) | 1.558(1.186-1.989) | 2.009(1.619-2.445) | 495.158(448.667-540.705) |
| ASPR (95% UI) | 25844.856(24157.719-27750.385) | 10709.669(9767.865-11756.696) | 2318.478(1894.984-2835.063) | 12.019(9.384-15.201) | 17.305(13.360-21.698) | 12787.385(11678.389-14025.329) |
| ASMR (95% UI) | 20.486(17.405-23.765) | 15.639(13.216-18.059) | 2.382(1.934-2.939) | 1.230(0.962-1.532) | 0.699(0.526-0.916) | 0.536(0.354-0.759) |
| ASDR (95% UI) | 631.121(535.676-731.747) | 479.319(405.526-555.493) | 72.990(58.327-90.490) | 37.597(28.518-47.100) | 27.227(21.488-34.589) | 13.988(9.071-19.726) |
| **2021** |  |  |  |  |  |  |
| Incidence (95% UI) | 10984618.094(10121971.311-11863094.650) | 956384.070(717724.636-1236377.631) | 430364.744(347696.407-528079.243) | 23483.743(17917.460-29443.023) | 13242.884(10363.121-16882.631) | 9561142.653(8776596.790-10366756.252) |
| Prevalence (95% UI) | 394433302.082(366030670.938-423675081.549) | 83045549.672(74545164.662-91217016.202) | 19829778.814(15664995.186-24260961.573) | 193023.617(151655.415-239639.983) | 117781.949(91048.920-148637.309) | 291247168.029(265154838.205-317831031.679) |
| Death (95% UI) | 156418.749(123264.259-191698.968) | 115932.760(91235.127-142893.192) | 16665.364(13022.564-21553.126) | 13395.476(9993.527-18080.901) | 4086.120(2785.408-5725.586) | 6339.029(4018.185-9241.388) |
| DALYS (95% UI) | 4500055.578(3552251.221-5555056.600) | 3348578.709(2631293.231-4157898.252) | 481674.137(373067.678-629745.523) | 397254.110(295809.992-539396.035) | 114286.053(81613.230-155857.155) | 158262.569(102325.488-231673.166) |
| ASIR (95% UI) | 720.205(663.331-780.328) | 60.982(47.217-76.562) | 35.853(29.096-43.404) | 1.102(0.859-1.359) | 1.083(0.860-1.323) | 621.184(565.753-677.230) |
| ASPR (95% UI) | 20302.624(18845.228-21791.911) | 3490.137(3197.573-3789.017) | 1704.785(1373.459-2061.699) | 34.811(28.882-40.257) | 55.442(46.652-64.851) | 15017.457(13755.839-16360.798) |
| ASMR (95% UI) | 7.688(6.080-9.375) | 5.715(4.520-7.028) | 0.818(0.640-1.047) | 0.627(0.472-0.842) | 0.213(0.154-0.288) | 0.314(0.200-0.452) |
| ASDR (95% UI) | 223.634(177.205-275.333) | 167.035(131.455-206.936) | 23.731(18.272-30.964) | 18.607(13.874-25.176) | 6.650(4.990-8.707)  \ | 7.611(5.007-10.941) |
| **1990-2021** |  |  |  |  |  |  |
| ASIR (EAPC,  95% CI) | -0.44(-0.65,-0.24) | -4.74(-4.95,-4.53) | -2.06(-2.35,-1.77) | -1.13(-1.25,-1.02) | -2.20(-2.34,-2.05) | 0.74(0.57,0.91) |
| ASPR (EAPC,  95% CI) | -0.71(-0.90,-0.52) | -2.88(-3.03,-2.74) | -2.42(-2.85,-1.98) | -0.83(-1.00,-0.65) | -2.26(-2.46,-2.05) | 0.67(0.44,0.91) |
| ASMR (EAPC,  95% CI) | -3.38(-3.52,-3.24) | -3.54(-3.72,-3.36) | -3.38(-3.53,-3.23) | -2.14(-2.30,-1.97) | -3.81(-3.96,-3.65) | -1.77(-1.92,-1.61) |
| ASDR (EAPC,  95% CI) | -3.60(-3.72,-3.47) | -3.72(-3.88,-3.56) | -3.61(-3.76,-3.46) | -2.25(-2.44,-2.06) | -4.58(-4.75,-4.40) | -2.03(-2.19,-1.87) |

DALYs disability-adjusted life-years, ASIR age-standardized incidence rate, ASPR age-standardized prevalence rate, ASMR age-standardized mortality rate, ASDR age-standardized DALYs rate, EAPC estimated annual percentage change, CI confdence interval, UI uncertainty intervals
