# Supplementary material for: Liver cirrhosis in 2021: Global Burden of Disease study
Source: PLoS One. 2025 Jul 18;20(7):e0328493. doi: 10.1371/journal.pone.0328493 (PMC12273999; doi:10.1371/journal.pone.0328493)
Supplement: S1 File — (PDF) [file pone.0328493.s003.pdf]

Liver cirrhosis in 2021: Global burden of disease study

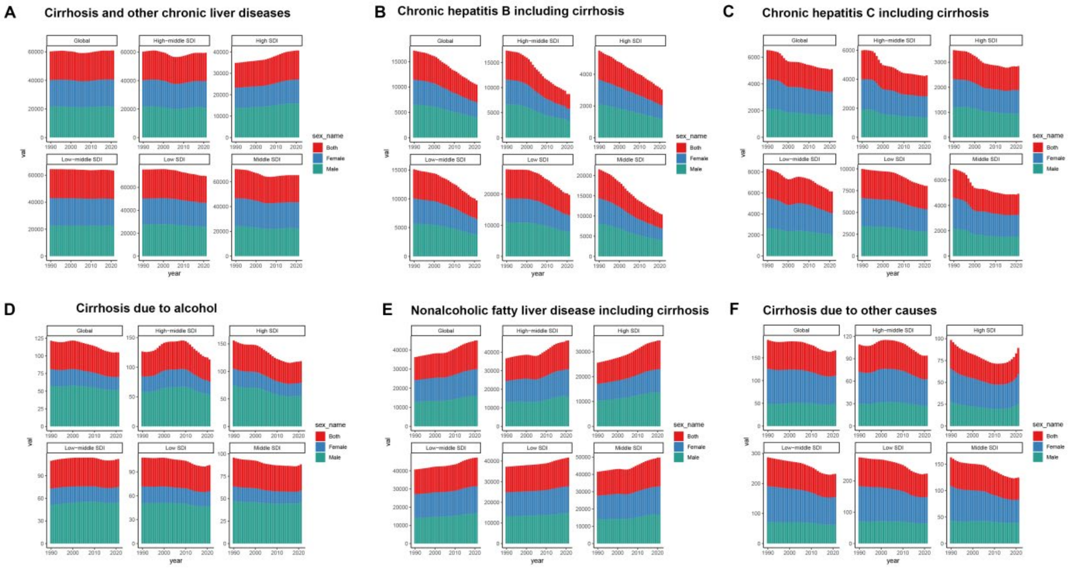

Fig.S1 Global and Regional Deaths Trends of Cirrhosis by Cause and SDI Levels

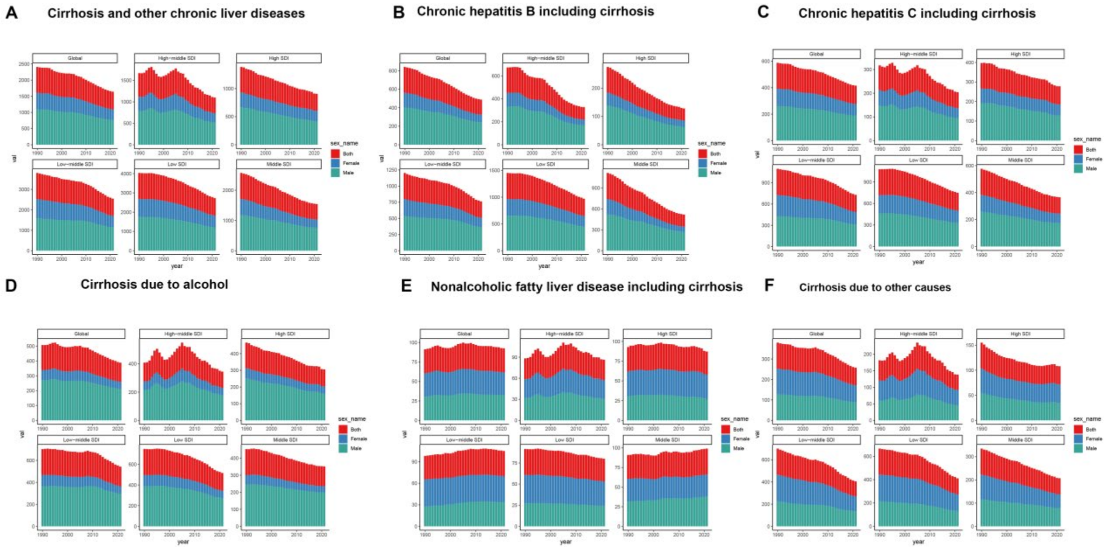

Fig.S2 Global and Regional DALYS Trends of Cirrhosis by Cause and SDI Levels

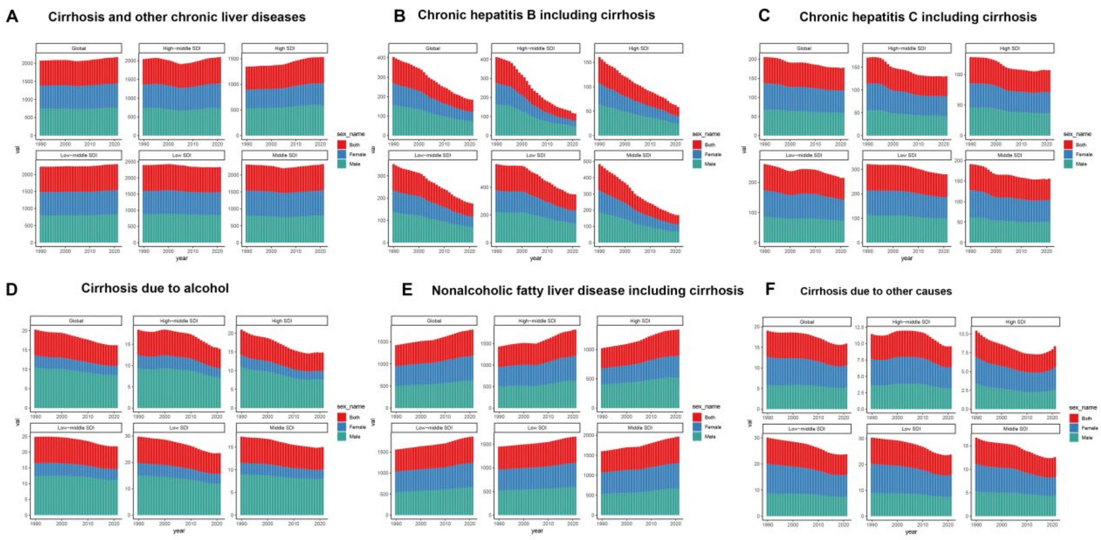

Fig.S3 Global and Regional Incidence Trends of Cirrhosis by Cause and SDI Levels

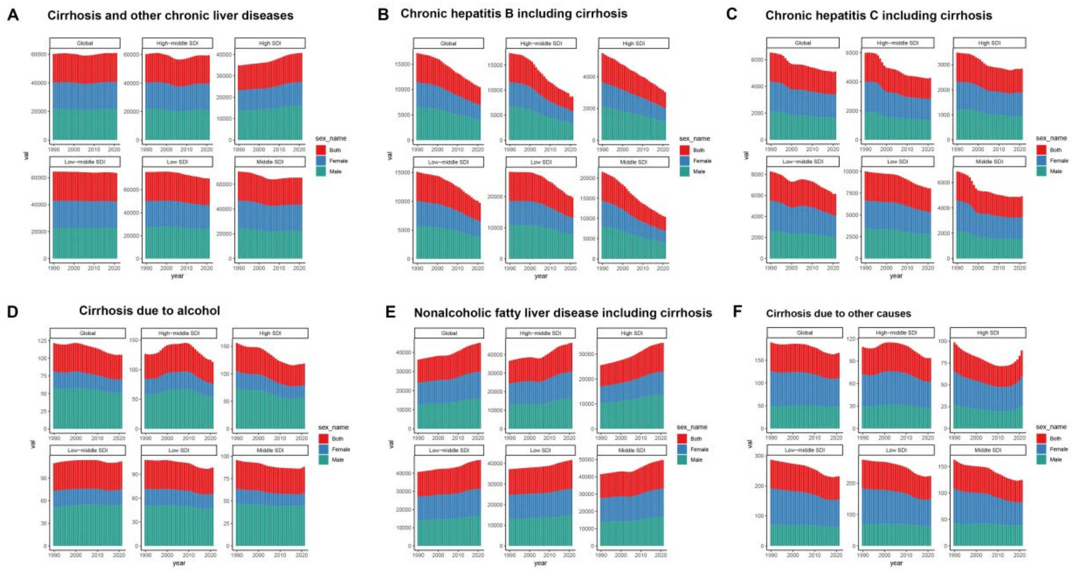

Fig.S4 Global and Regional Prevalence Trends of Cirrhosis by Cause and SDI Levels

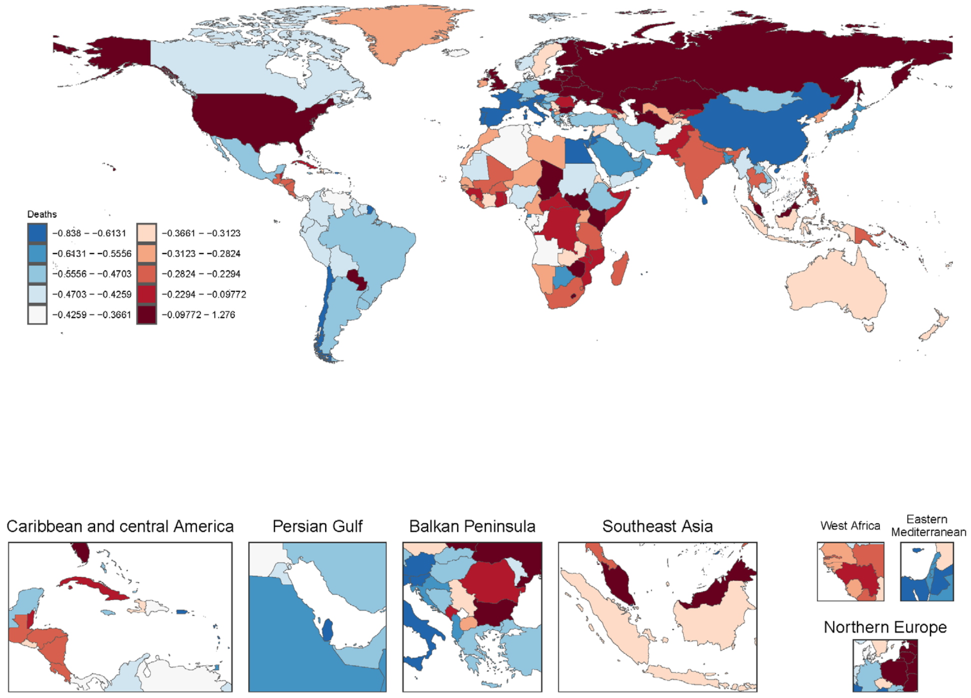

Fig.S5a Global Trends in Age-Standardized Percentage Change of Cirrhosis due to hepatitis B Deaths, 1990–2021

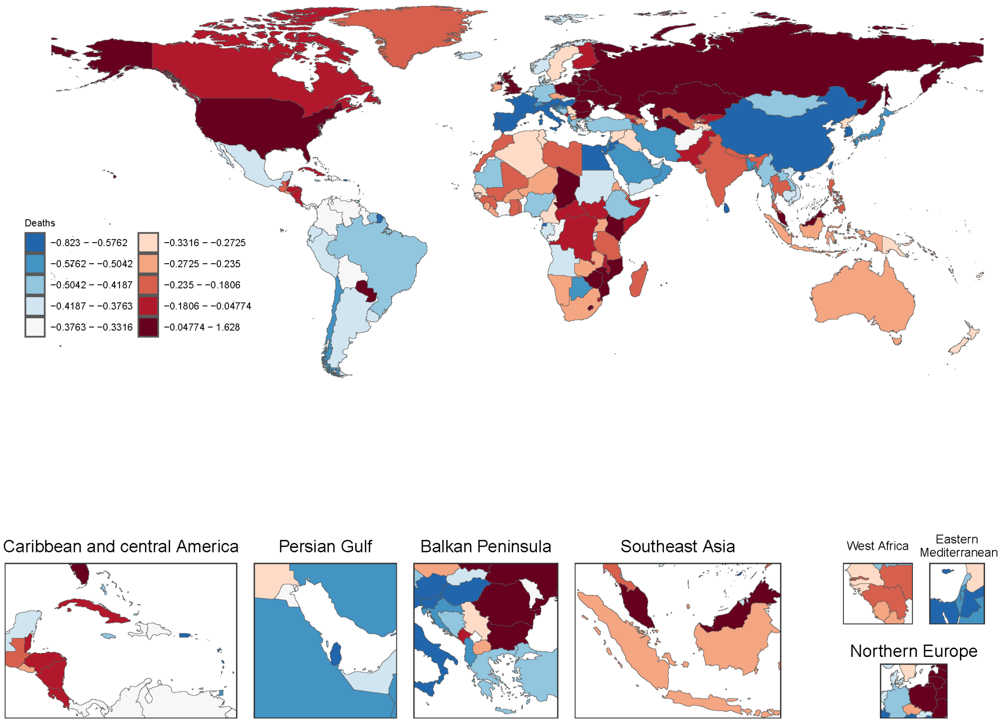

Fig. S5b Global Trends in Age-Standardized Percentage Change of Cirrhosis due to hepatitis C Deaths, 1990–2021

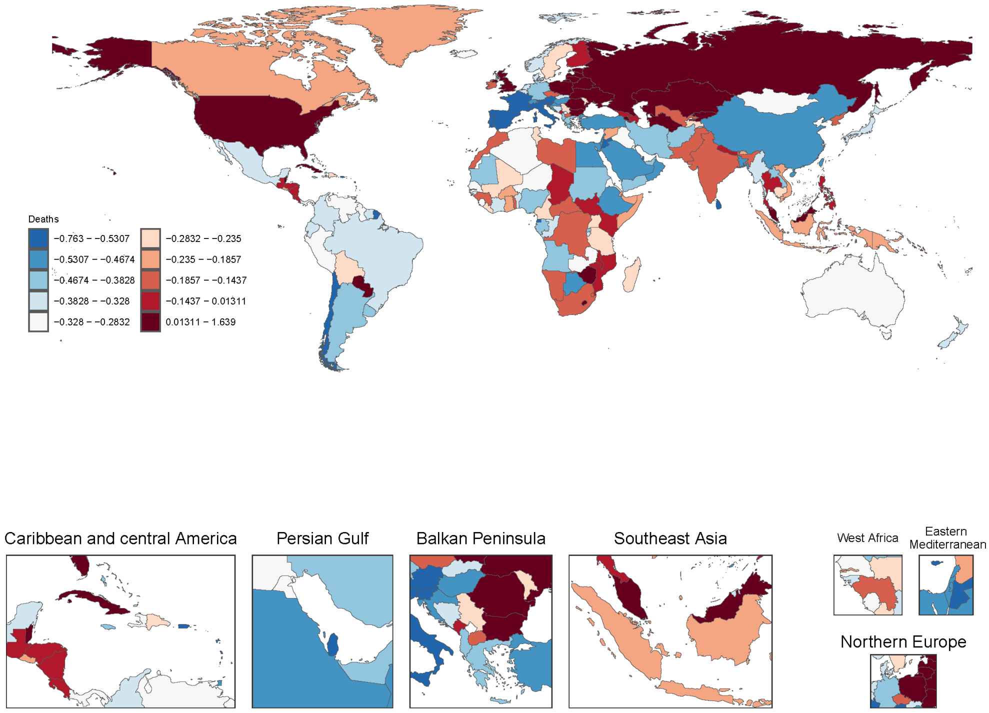

Fig.S5c Global Trends in Age-Standardized Percentage Change of Cirrhosis due to alcohol Deaths, 1990–2021

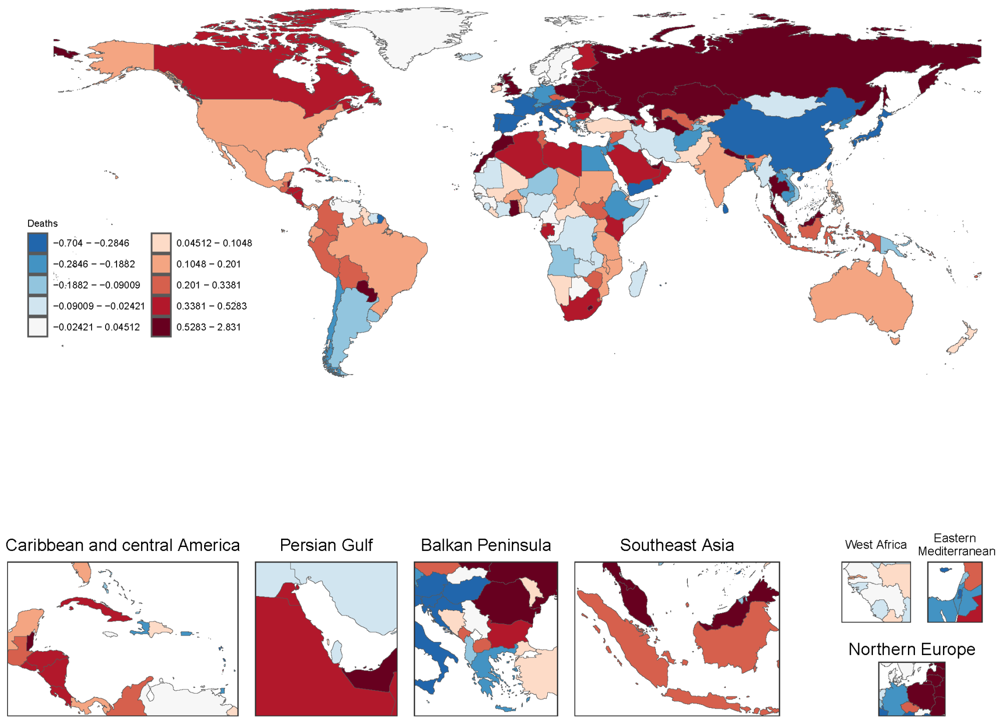

Fig.S5d Global Trends in Age-Standardized Percentage Change of Cirrhosis due to NAFLD Deaths, 1990–2021

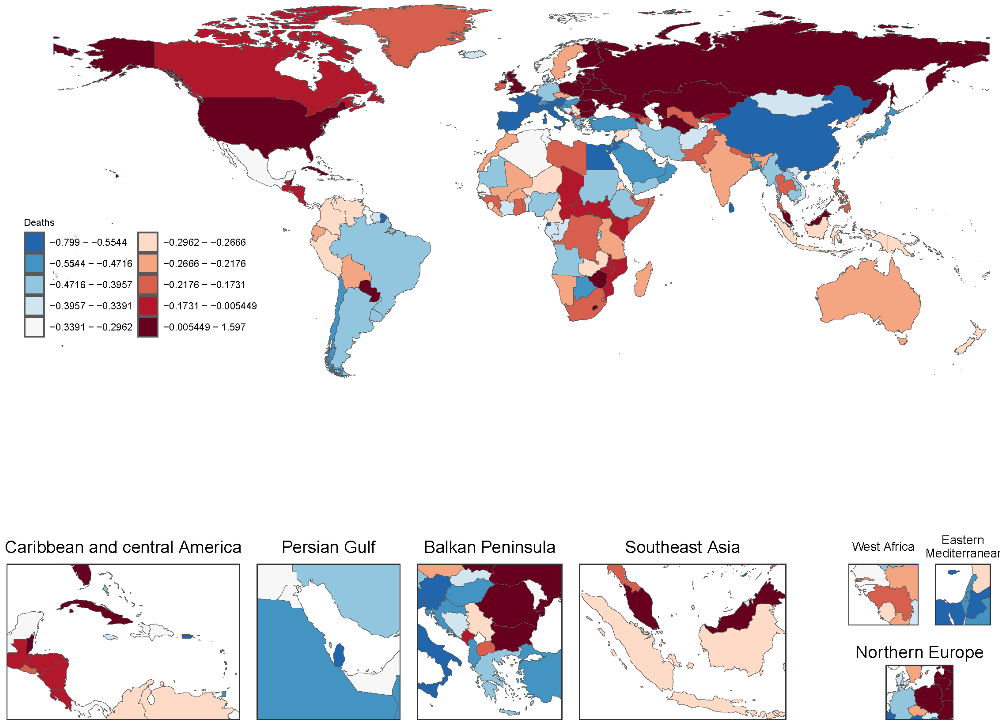

Fig.S5e Global Trends in Age-Standardized Percentage Change of Cirrhosis due to other causes Deaths, 1990–2021

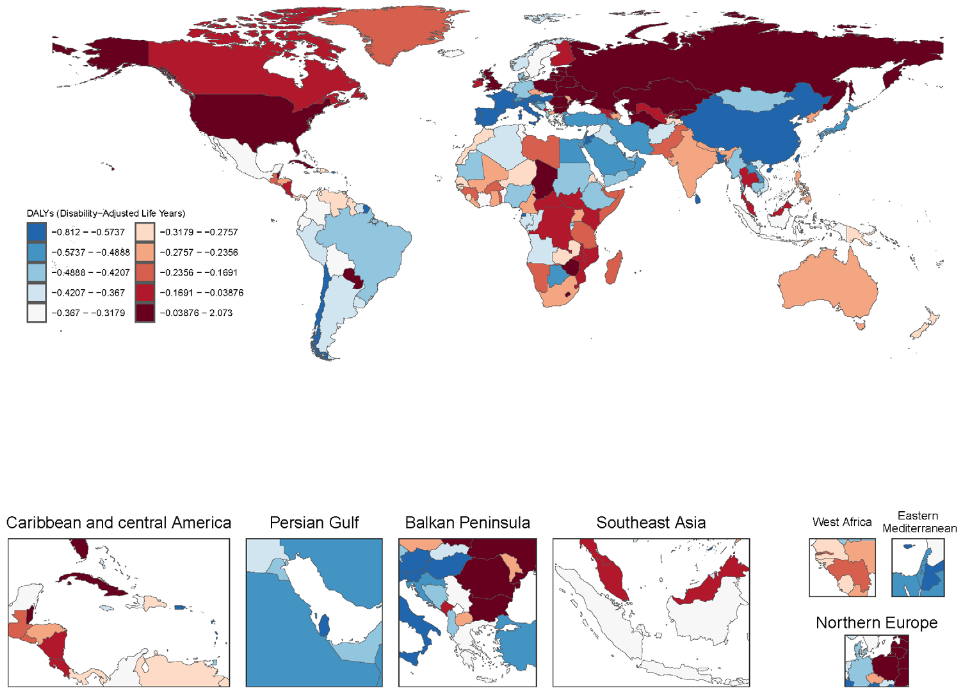

Fig.S6a Global Trends in Age-Standardized Percentage Change of Cirrhosis DALYS, 1990–2021

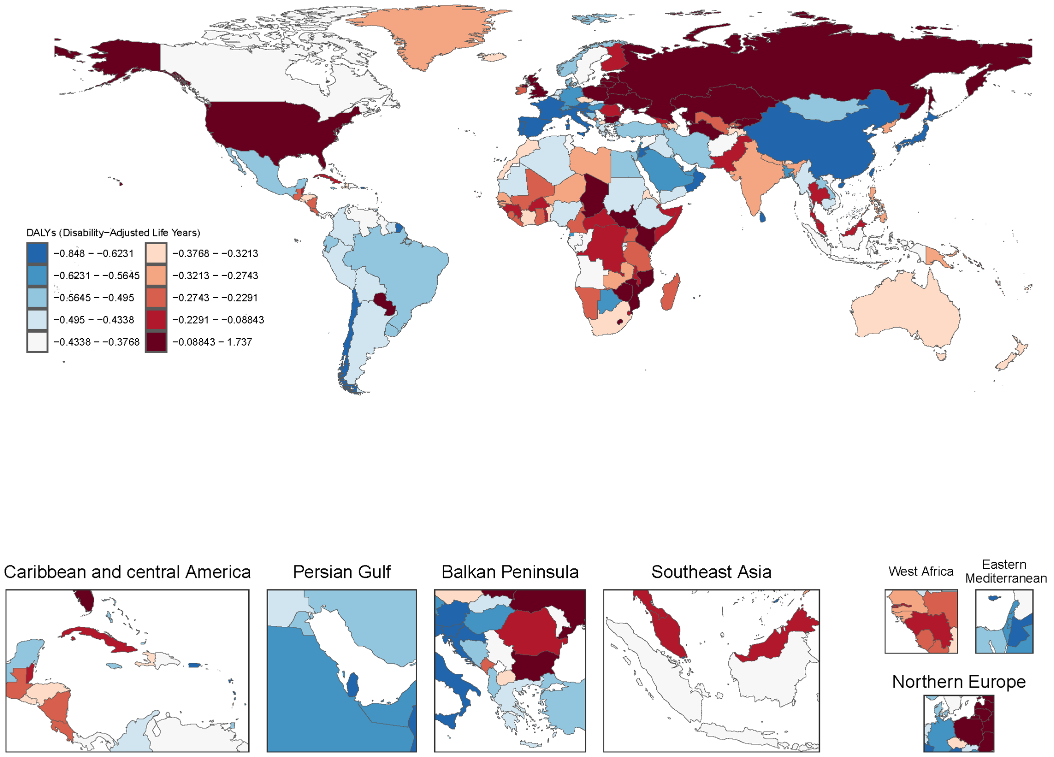

Fig.S6b Global Trends in Age-Standardized Percentage Change of Cirrhosis due to hepatitis B DALYS, 1990–2021

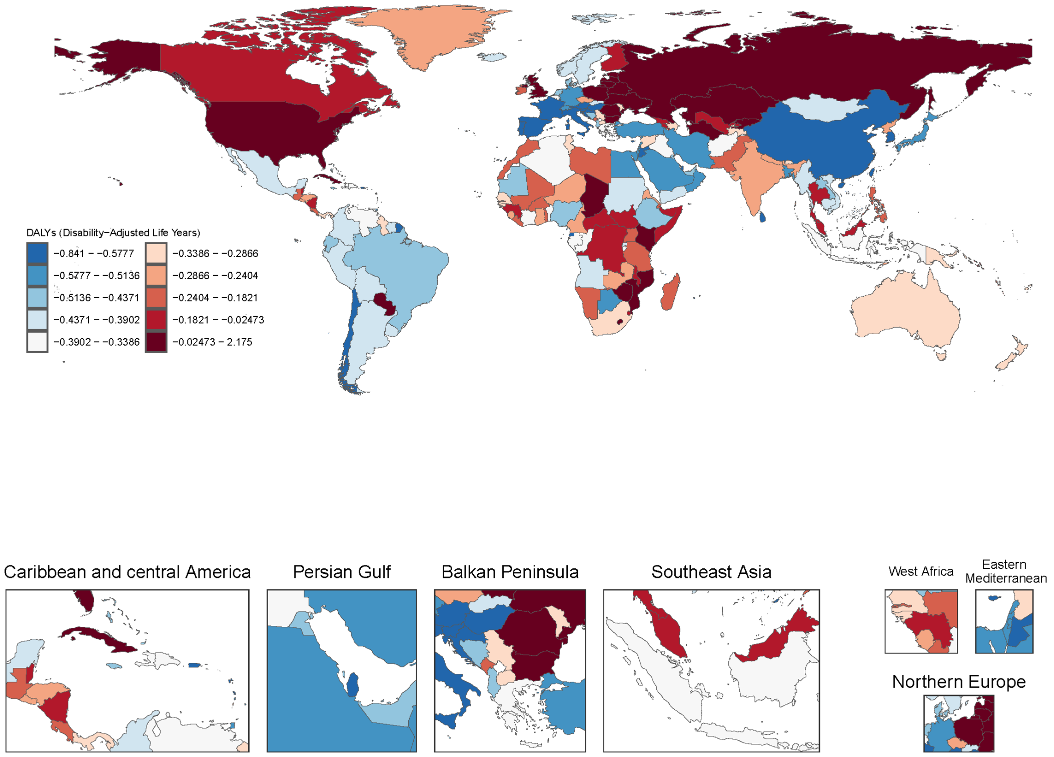

Fig.S6c Global Trends in Age-Standardized Percentage Change of Cirrhosis due to hepatitis C DALYS, 1990–2021

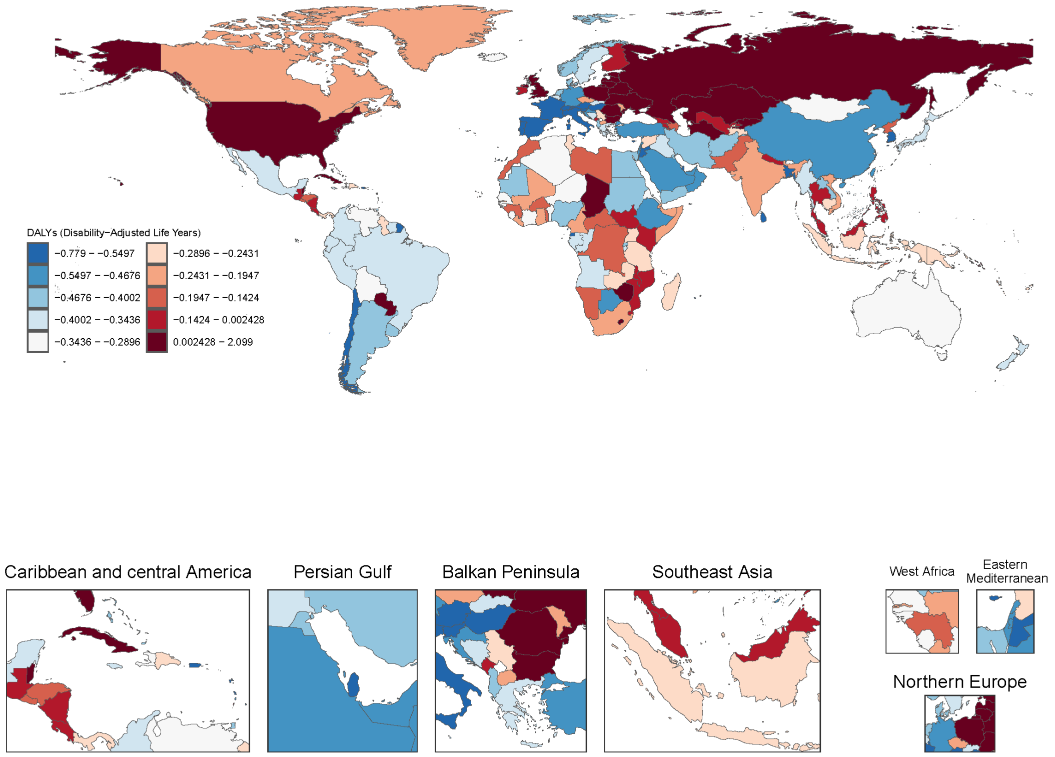

Fig.S6d Global Trends in Age-Standardized Percentage Change of Cirrhosis due to alcohol DALYS, 1990–2021

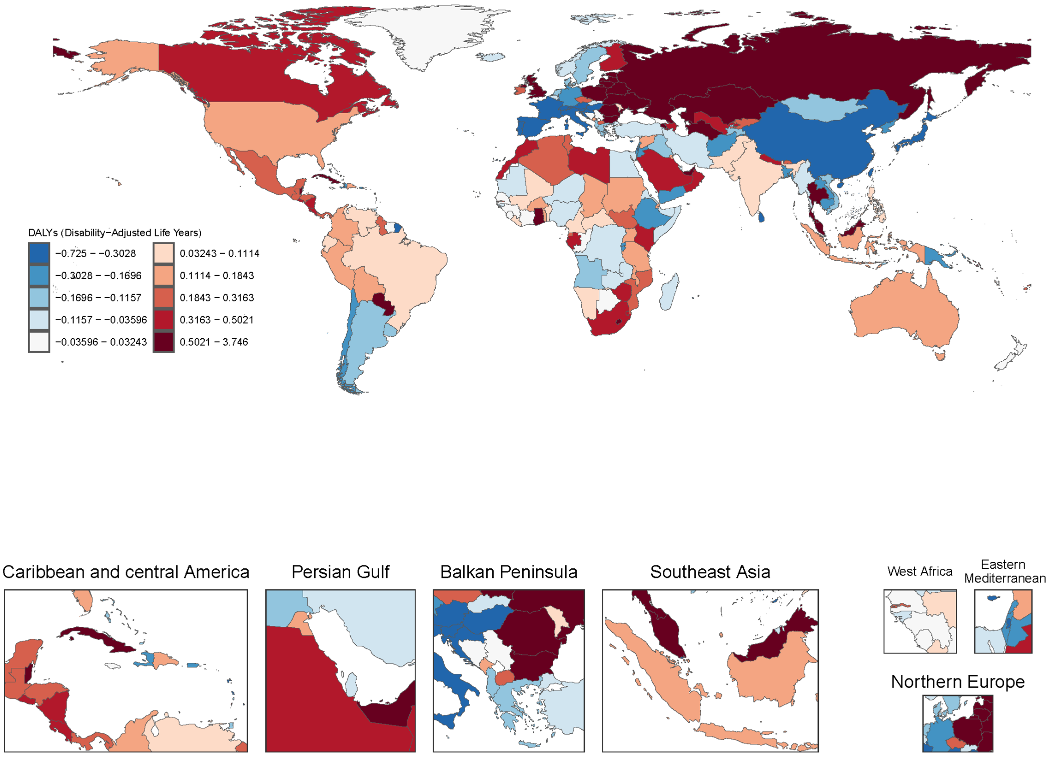

Fig.S6e Global Trends in Age-Standardized Percentage Change of Cirrhosis due to NAFLD DALYS, 1990–2021

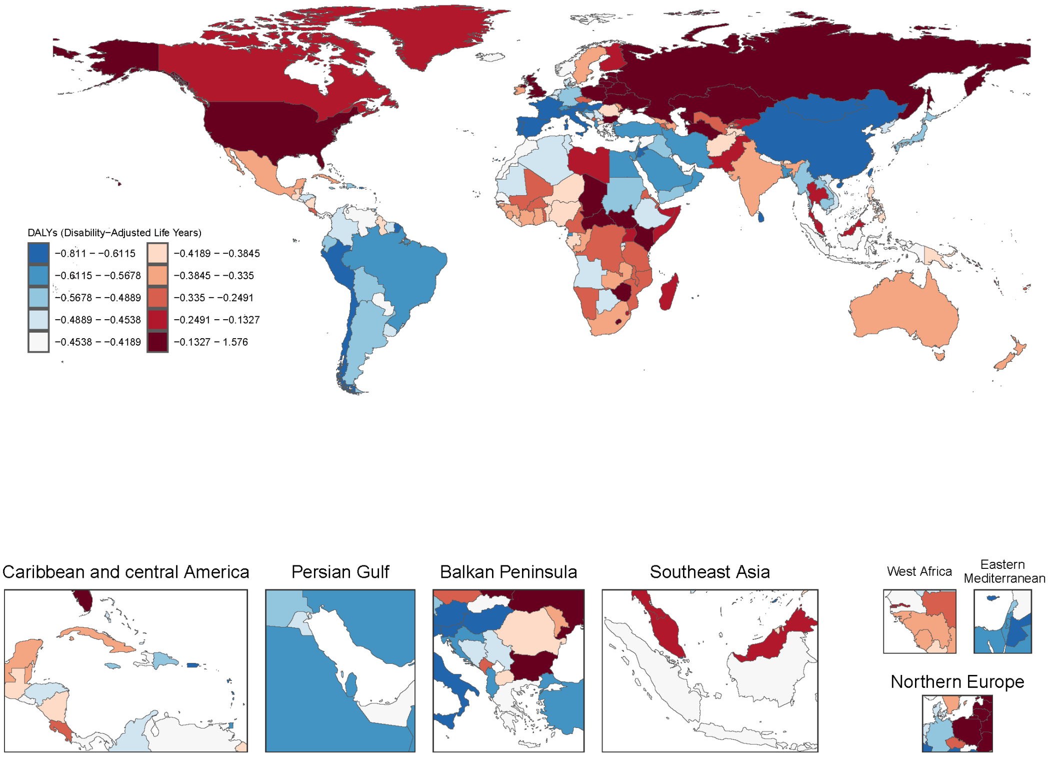

Fig.S6f Global Trends in Age-Standardized Percentage Change of Cirrhosis due to other causes DALYS, 1990–2021

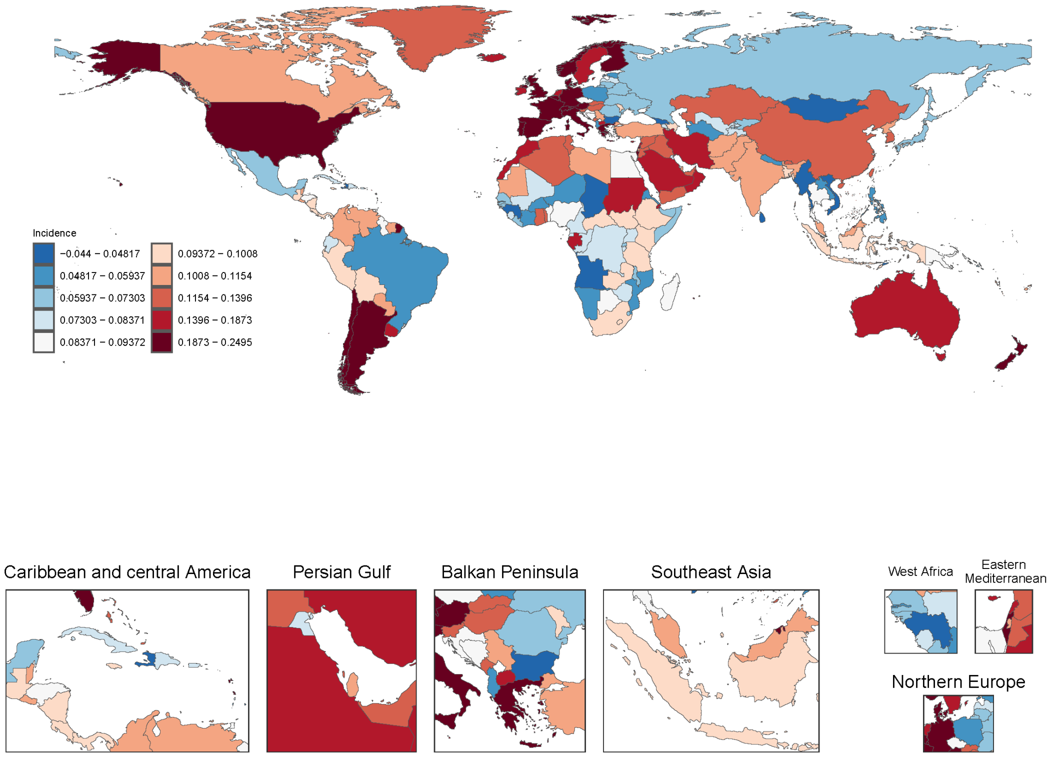

Fig.S7a Global Trends in Age-Standardized Percentage Change of Cirrhosis Incidence, 1990–2021

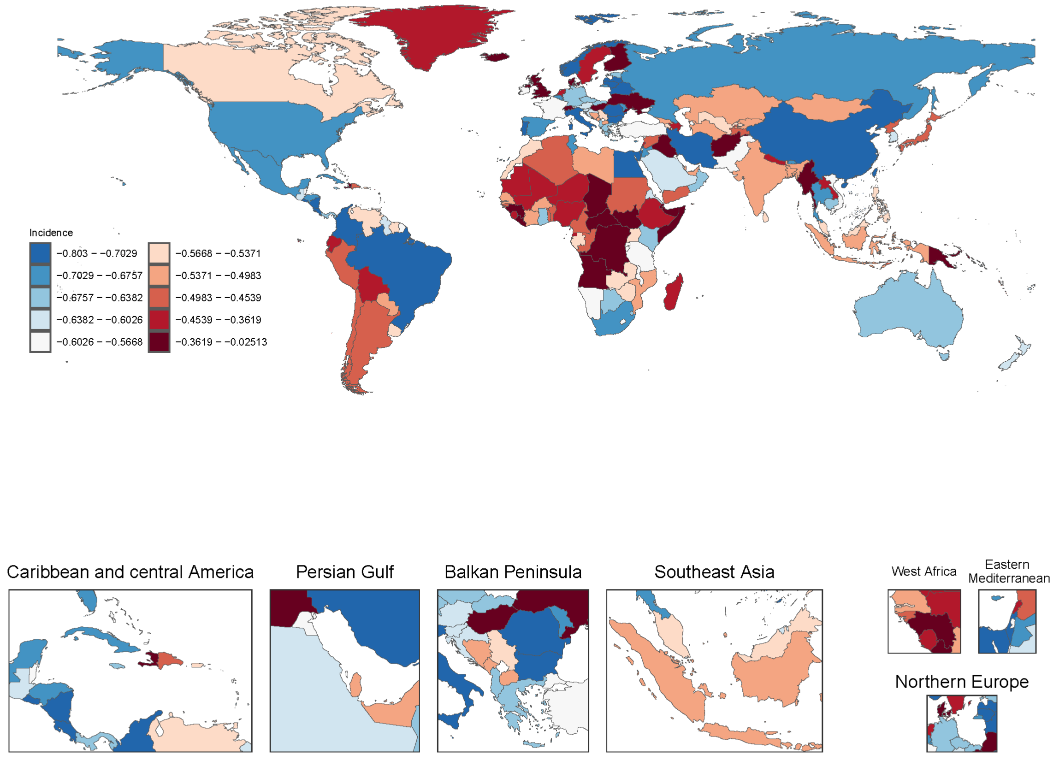

Fig.S7b Global Trends in Age-Standardized Percentage Change of Cirrhosis due to hepatitis B Incidence, 1990–2021

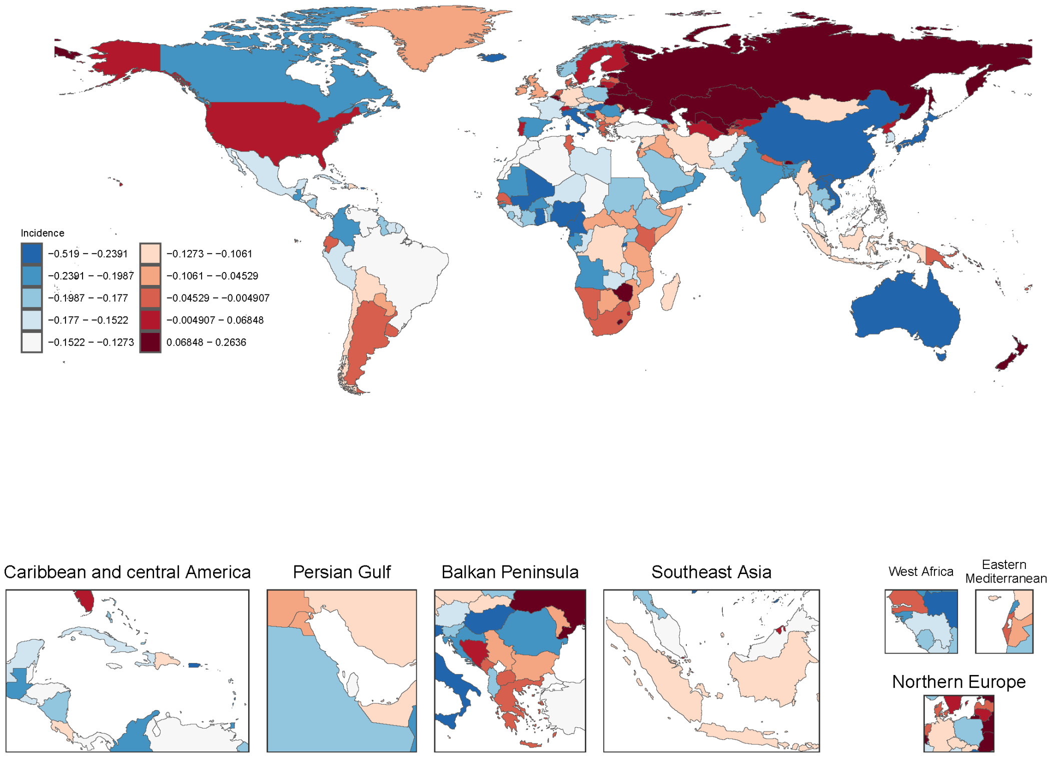

Fig.S7c Global Trends in Age-Standardized Percentage Change of Cirrhosis due to hepatitis C Incidence, 1990–2021

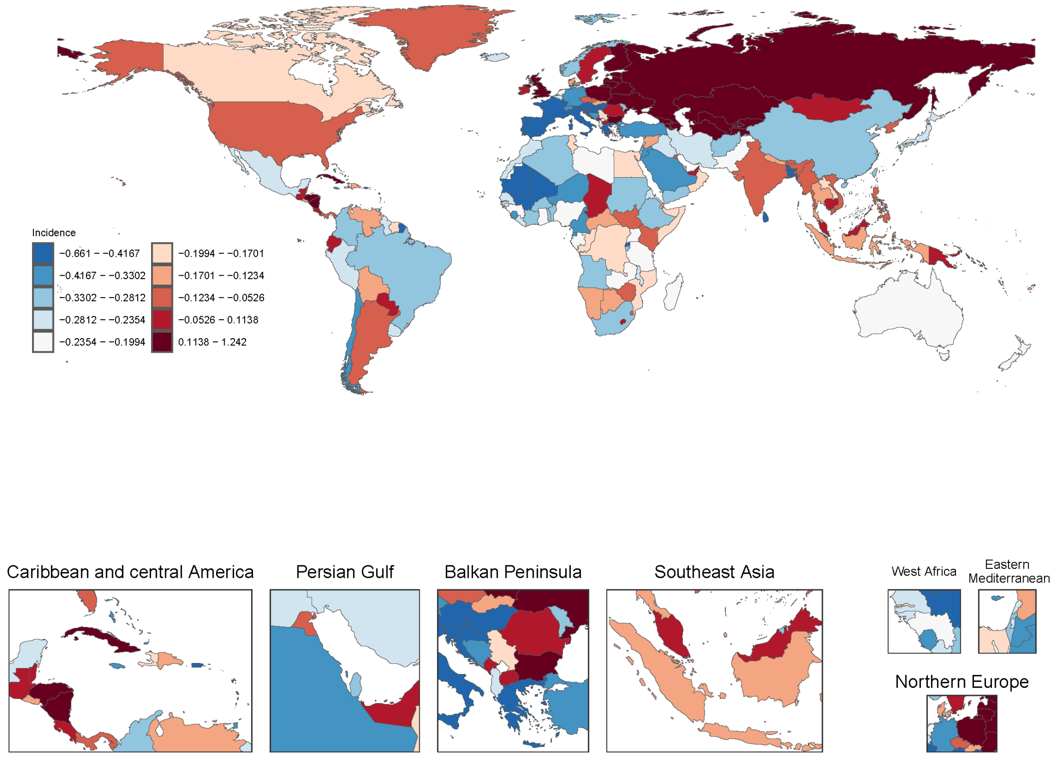

Fig.S7d Global Trends in Age-Standardized Percentage Change of Cirrhosis due to alcohol Incidence, 1990–2021

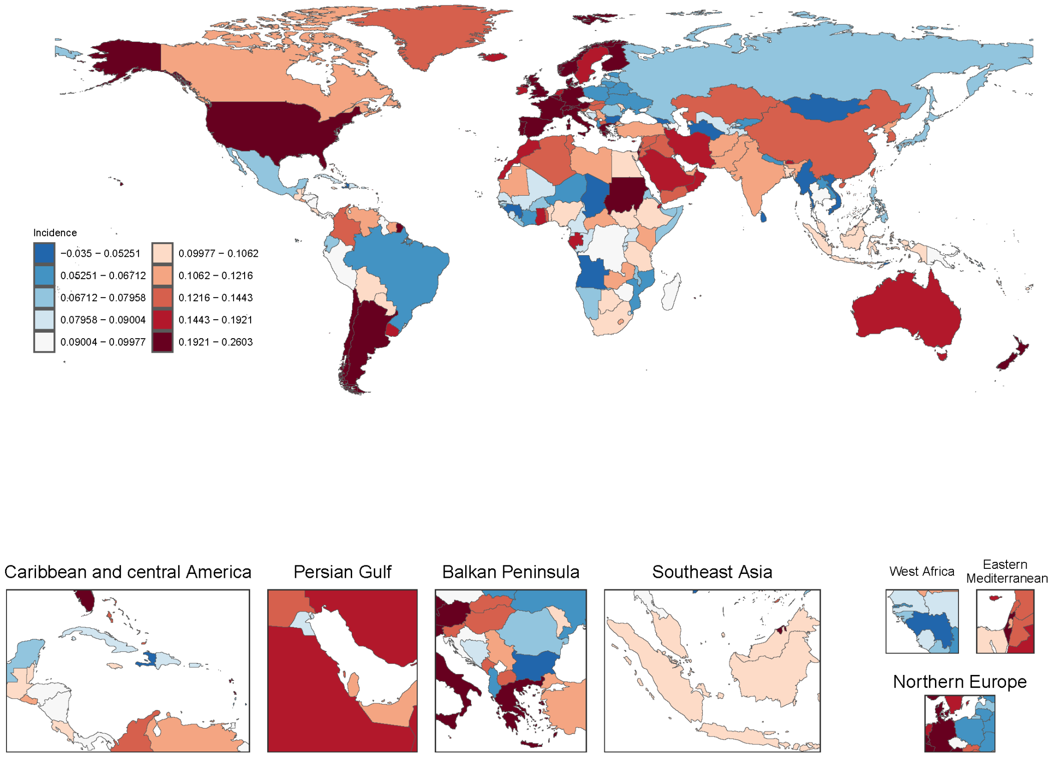

Fig.S7e Global Trends in Age-Standardized Percentage Change of Cirrhosis due to NAFLD Incidence, 1990–2021

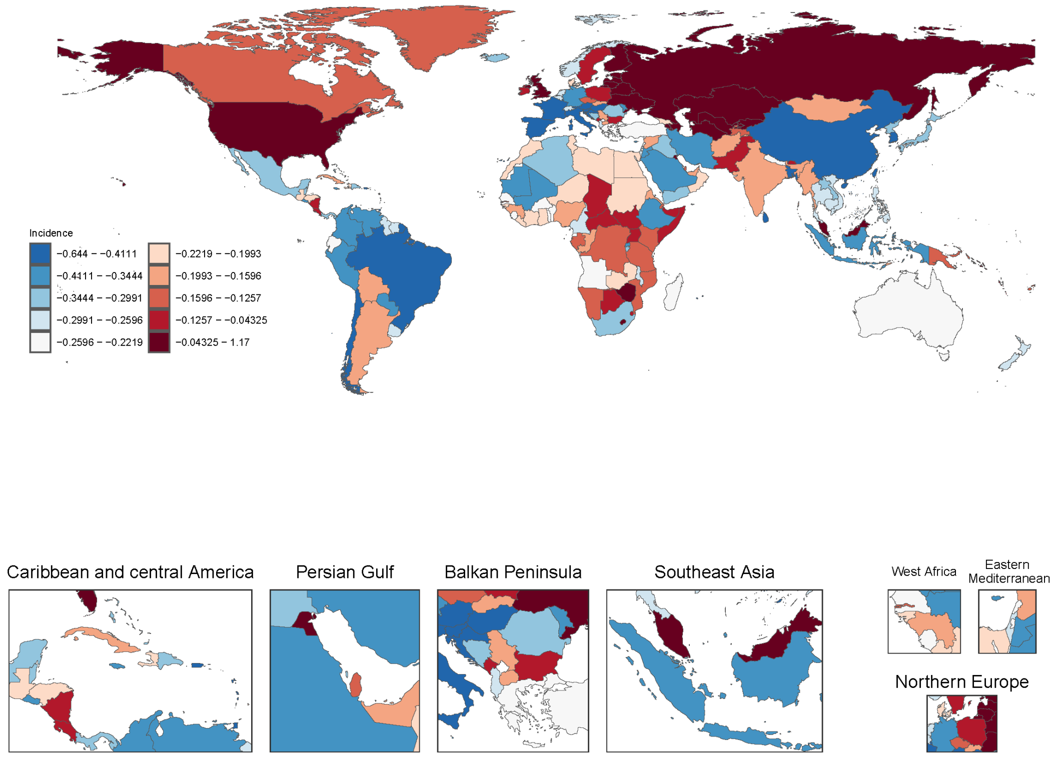

Fig.S7f Global Trends in Age-Standardized Percentage Change of Cirrhosis due to other causes Incidence, 1990–2021

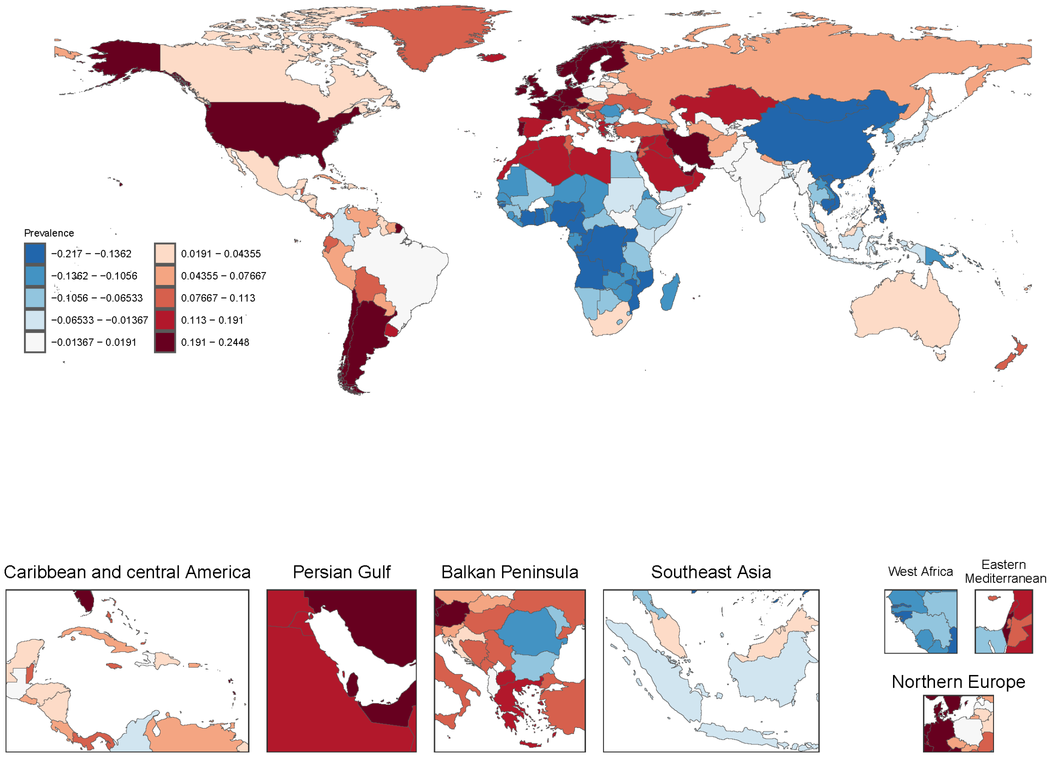

Fig.S8a Global Trends in Age-Standardized Percentage Change of Cirrhosis Prevalence, 1990–2021

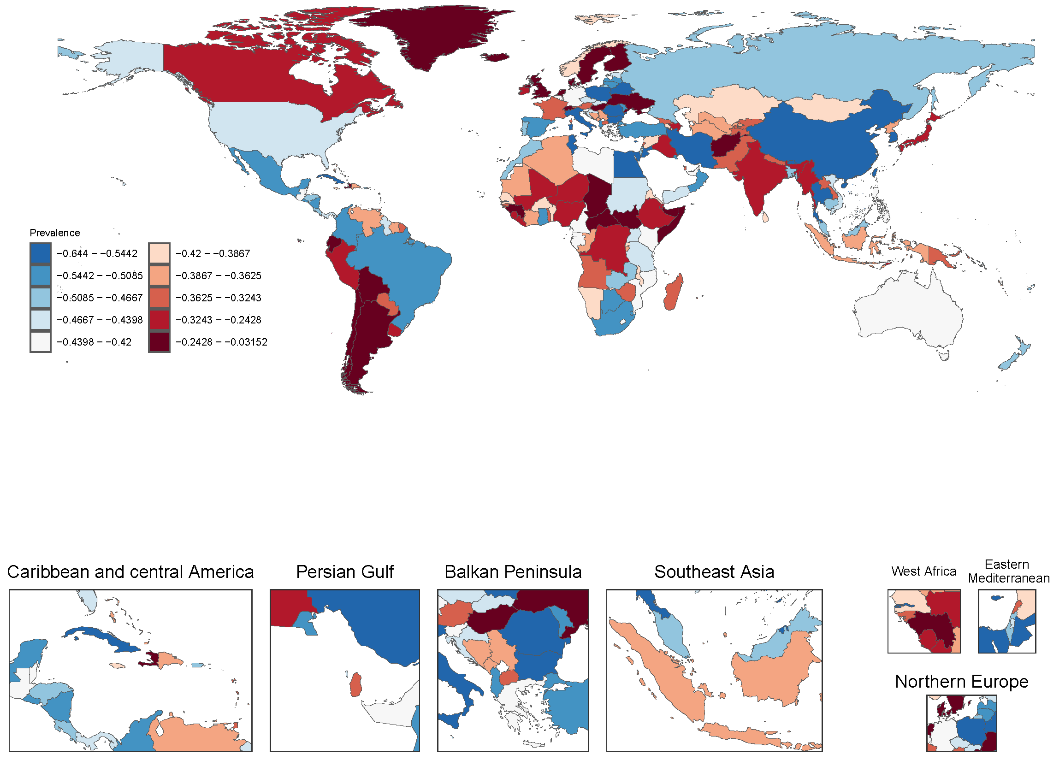

Fig.S8b Global Trends in Age-Standardized Percentage Change of Cirrhosis due to hepatitis B Prevalence, 1990–2021

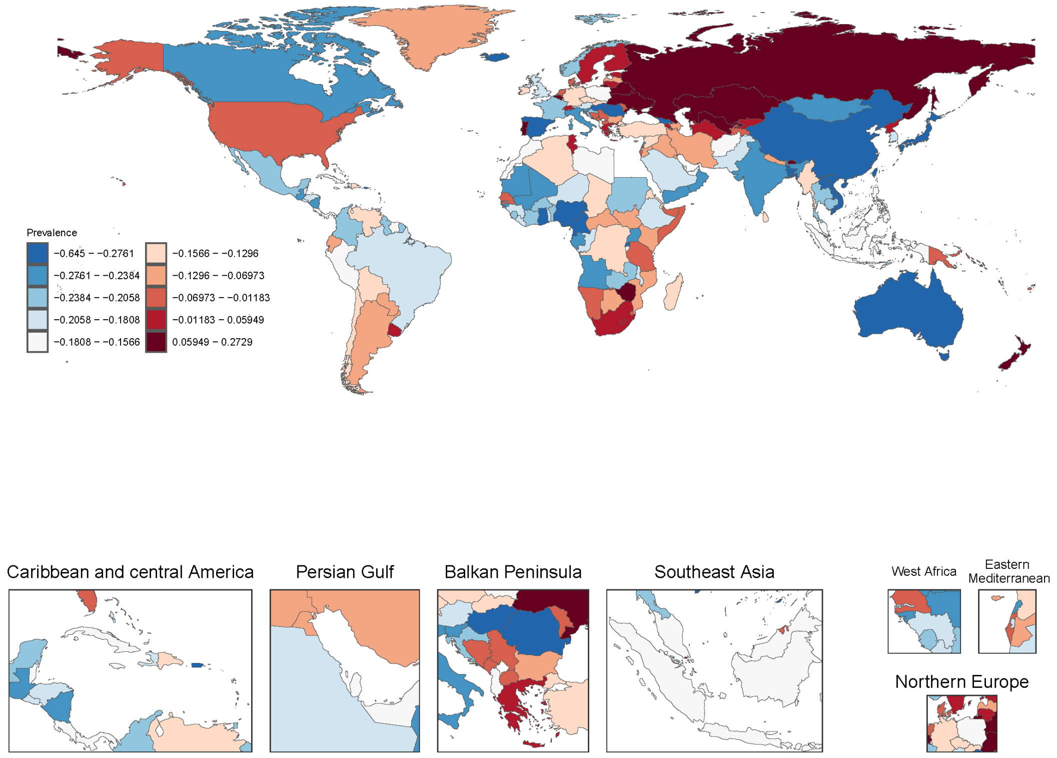

Fig.S8c Global Trends in Age-Standardized Percentage Change of Cirrhosis due to hepatitis C Prevalence, 1990–2021

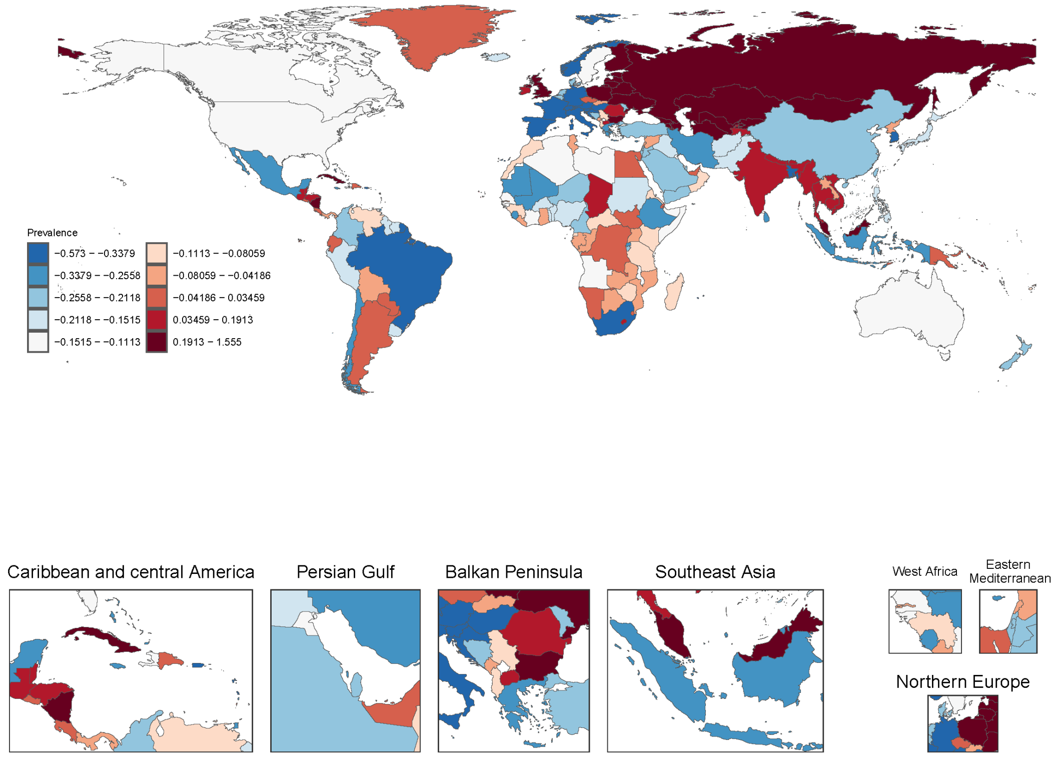

Fig.S8d Global Trends in Age-Standardized Percentage Change of Cirrhosis due to alcohol Prevalence, 1990–2021

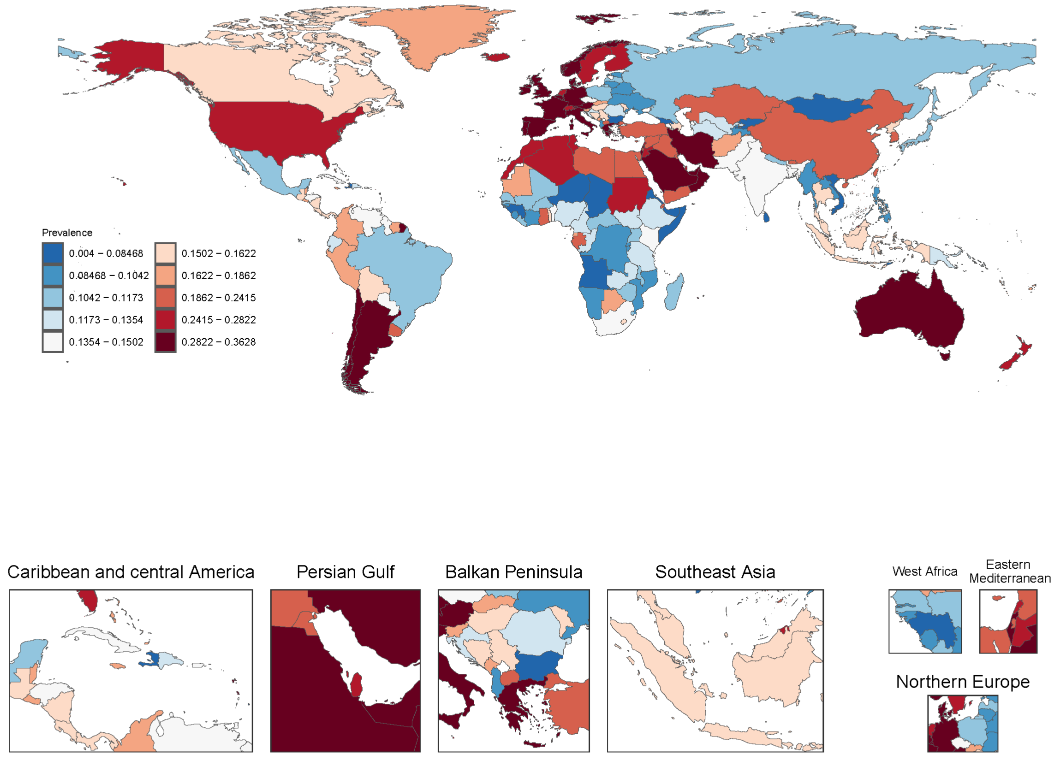

Fig.S8e Global Trends in Age-Standardized Percentage Change of Cirrhosis due to NAFLD Prevalence, 1990–2021

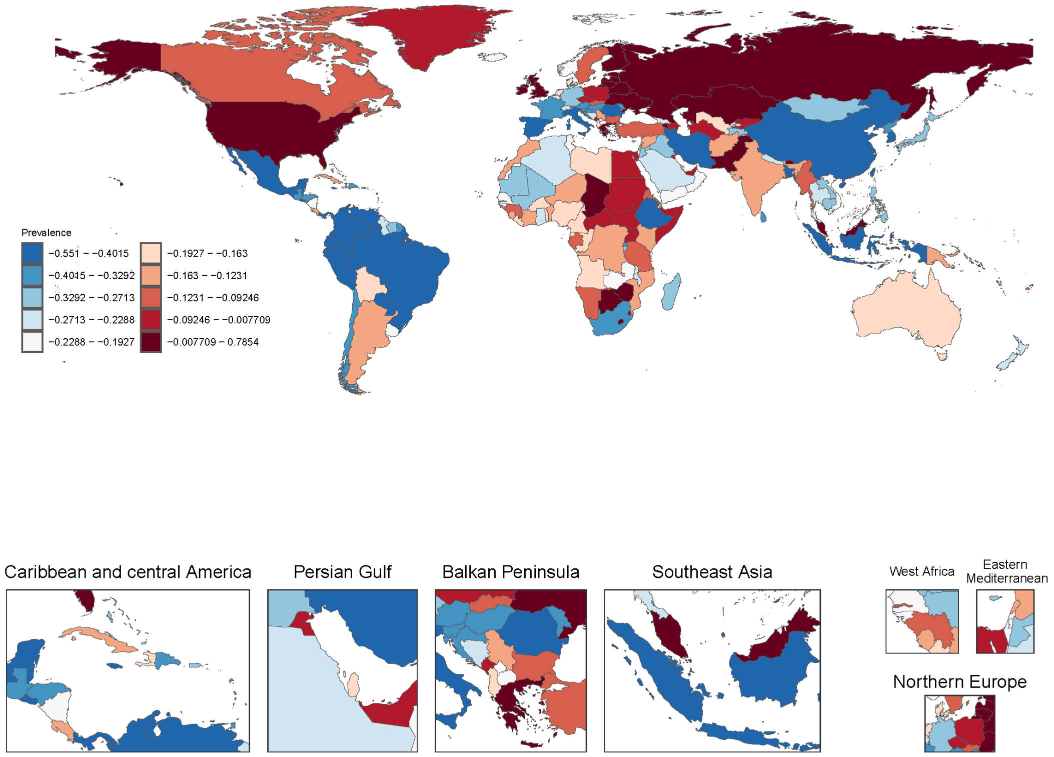

Fig.S8f Global Trends in Age-Standardized Percentage Change of Cirrhosis due to other causes Prevalence, 1990–2021

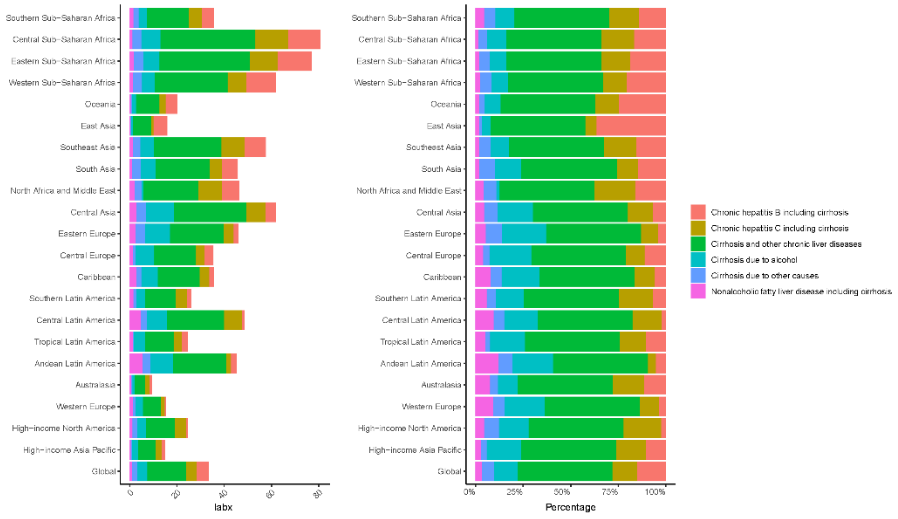

Fig. S9a Regional Deaths and Proportional Causes of Cirrhosis in Both Sexes, 2021

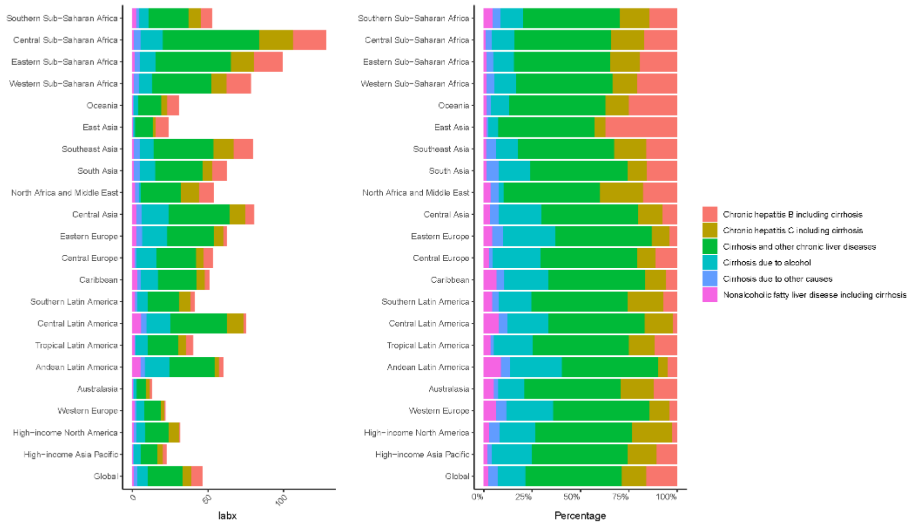

Fig. S9b Regional Deaths and Proportional Causes of Cirrhosis in Male, 2021

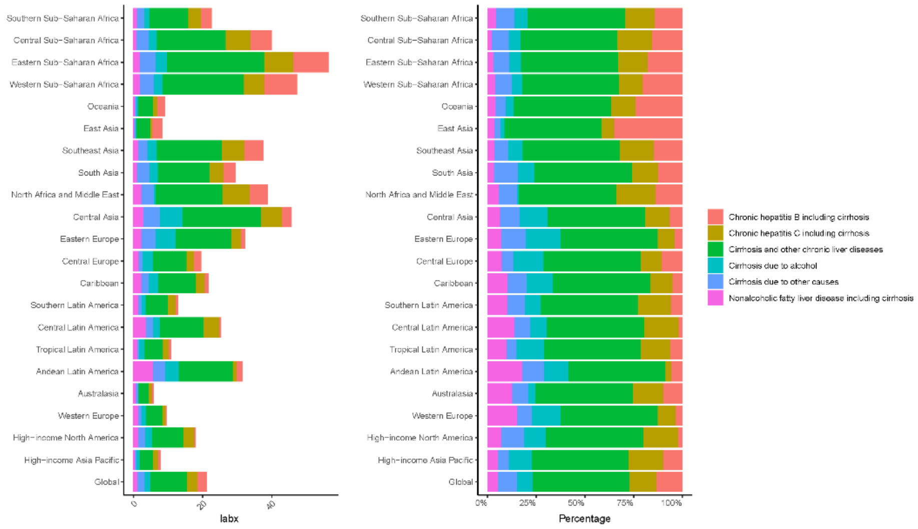

Fig. S9c Regional Deaths and Proportional Causes of Cirrhosis in Female, 2021

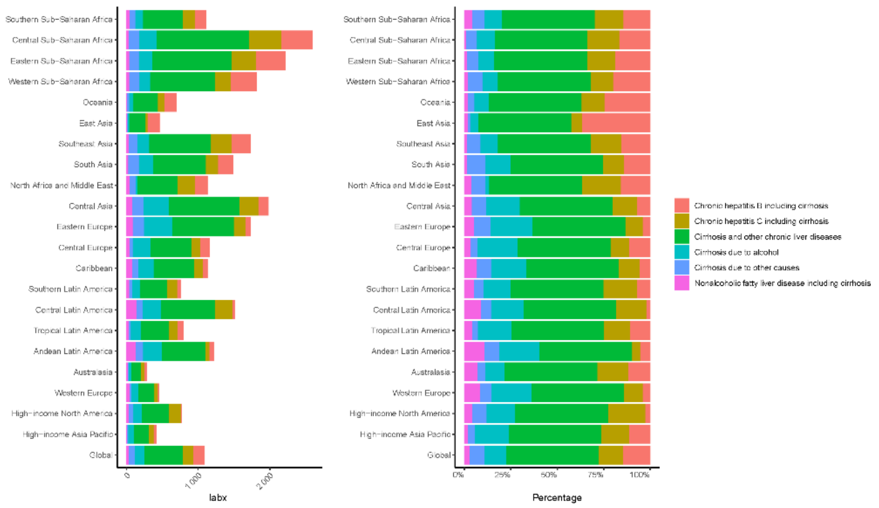

Fig. S10a Regional DALYs and Proportional Causes of Cirrhosis in Both Sexes, 2021

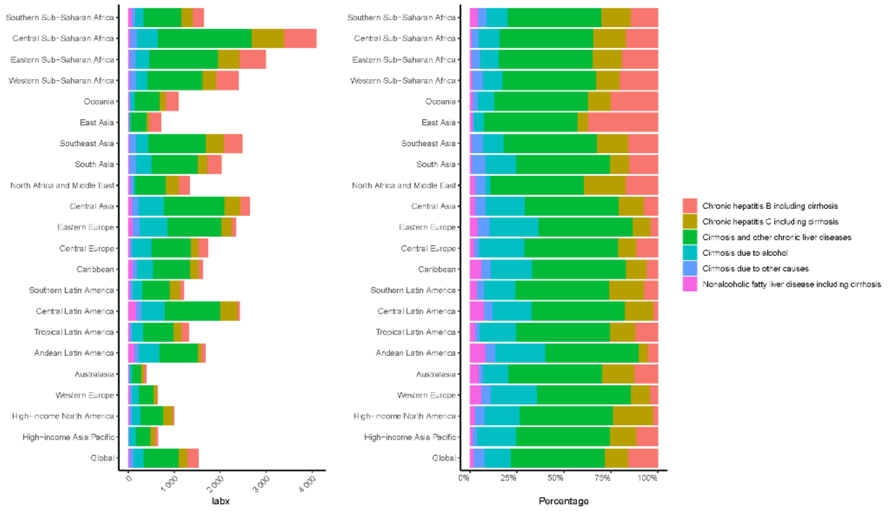

Fig. S10b Regional DALYs and Proportional Causes of Cirrhosis in Male, 2021

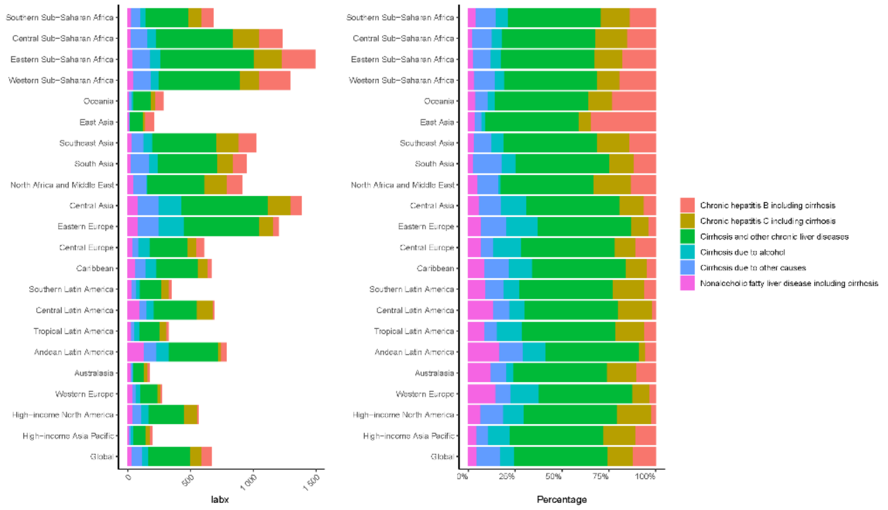

Fig. S10c Regional DALYs and Proportional Causes of Cirrhosis in Female, 2021

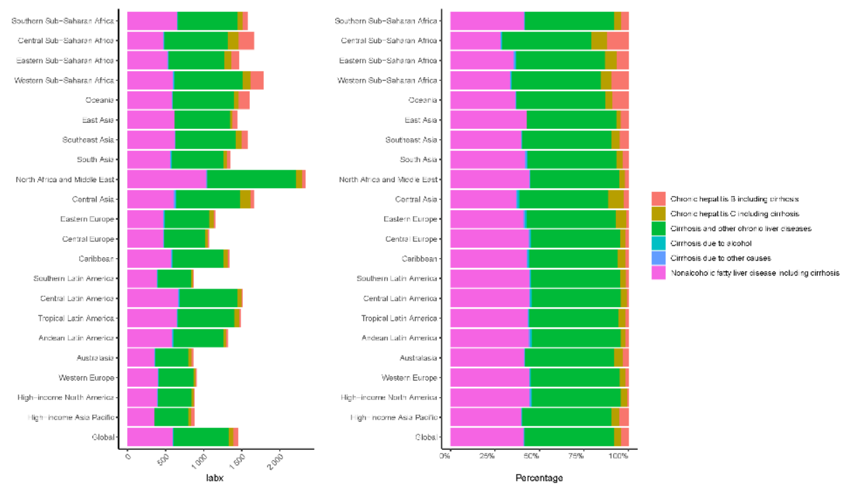

Fig. S11a Regional Incidence and Proportional Causes of Cirrhosis in Both Sexes, 2021

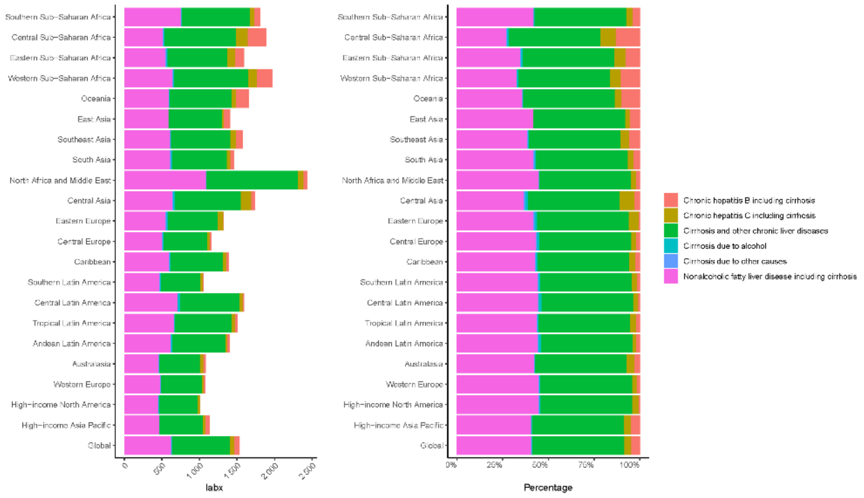

Fig. S11b Regional Incidence and Proportional Causes of Cirrhosis in Male, 2021

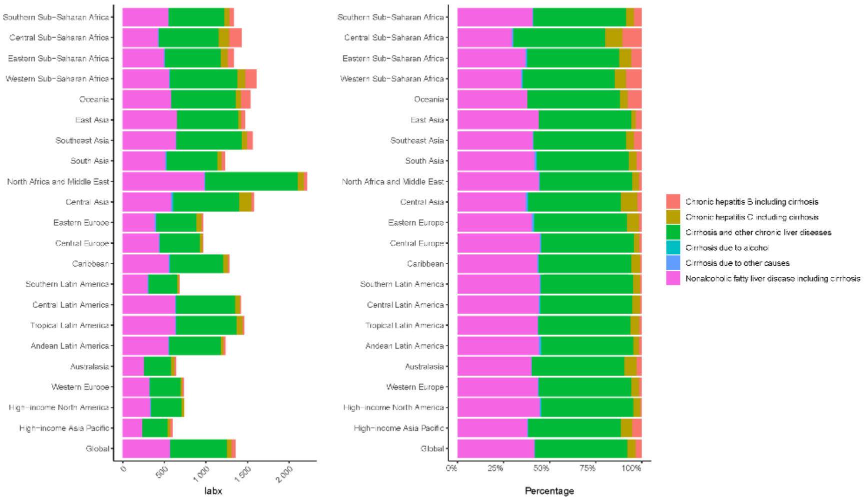

Fig. S11c Regional Incidence and Proportional Causes of Cirrhosis in Female, 2021

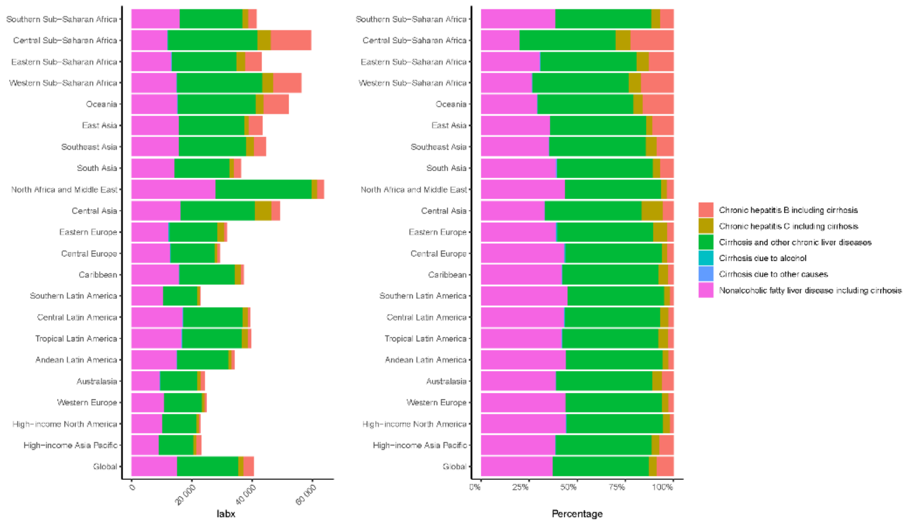

Fig. S12a Regional Prevalence and Proportional Causes of Cirrhosis in Both Sexes, 2021

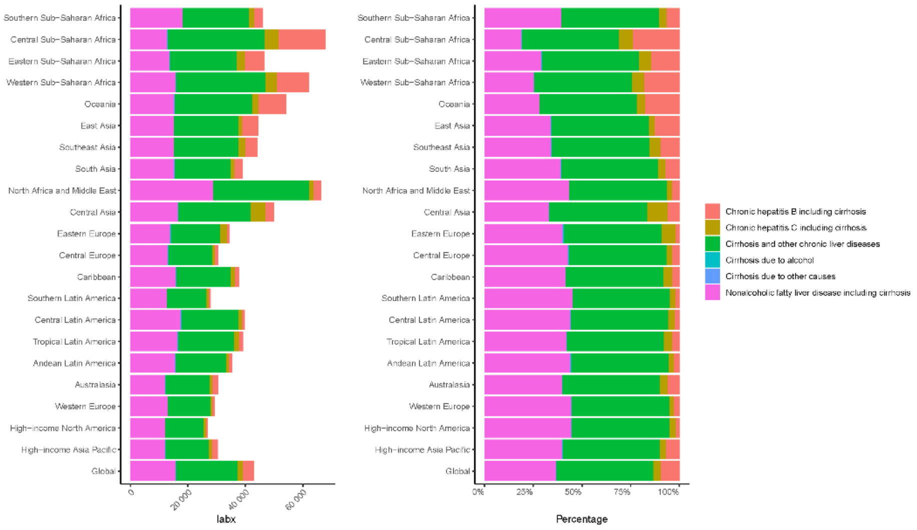

Fig. S12b Regional Prevalence and Proportional Causes of Cirrhosis in Male, 2021

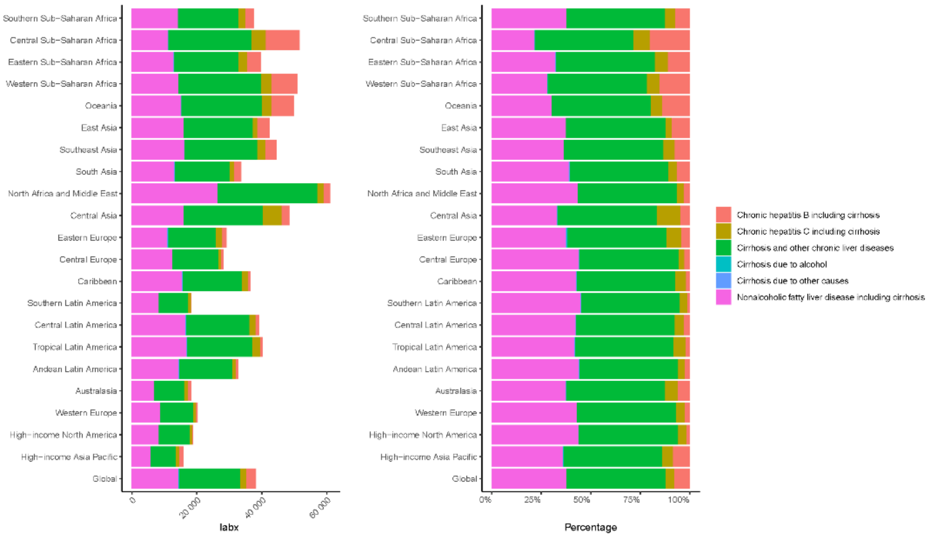

Fig. S12c Regional Prevalence and Proportional Causes of Cirrhosis in Female, 2021

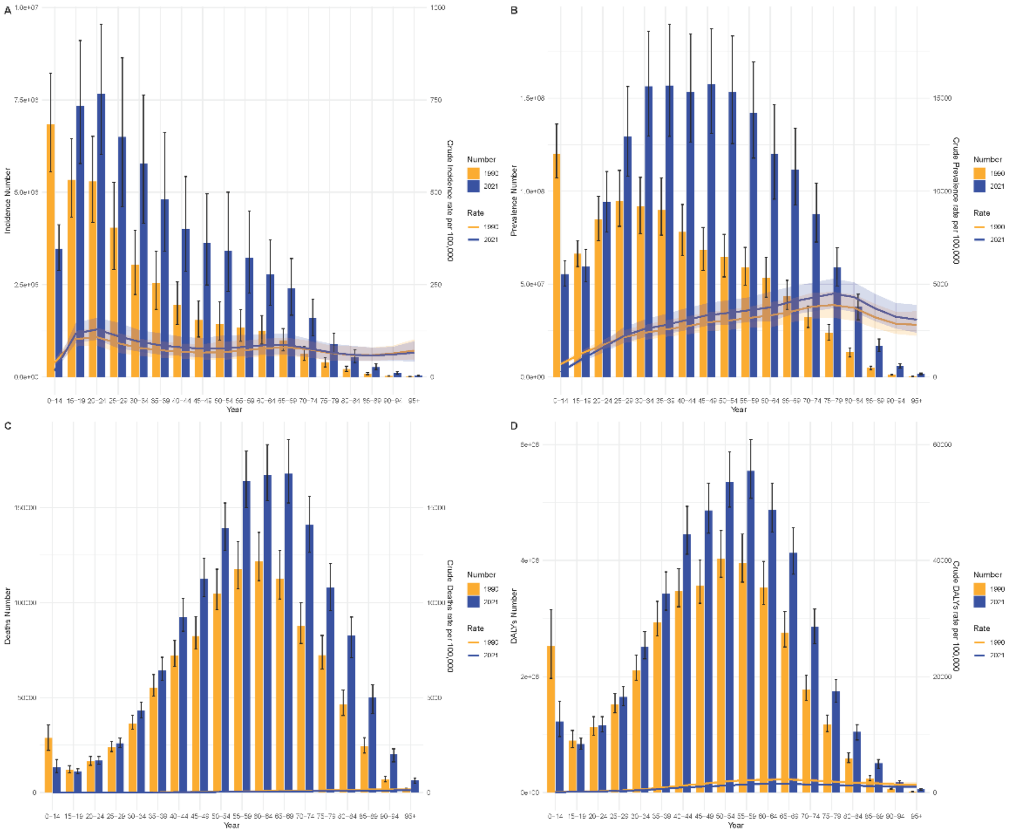

Fig.S13a Global Age-specific incidence, prevalence, deaths, and DALYs of Cirrhosis, 1990 and 2021

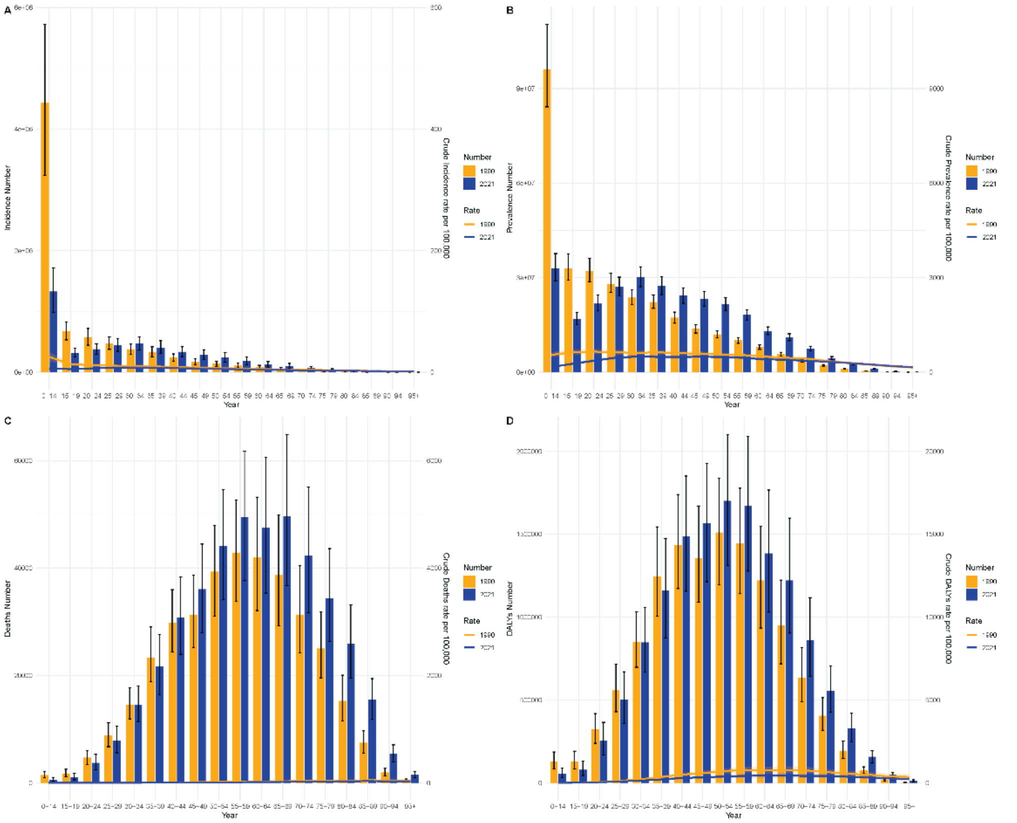

Fig.S13b Global Age-specific incidence, prevalence, deaths, and DALYs of Cirrhosis

due to hepatitis B, 1990 and 2021

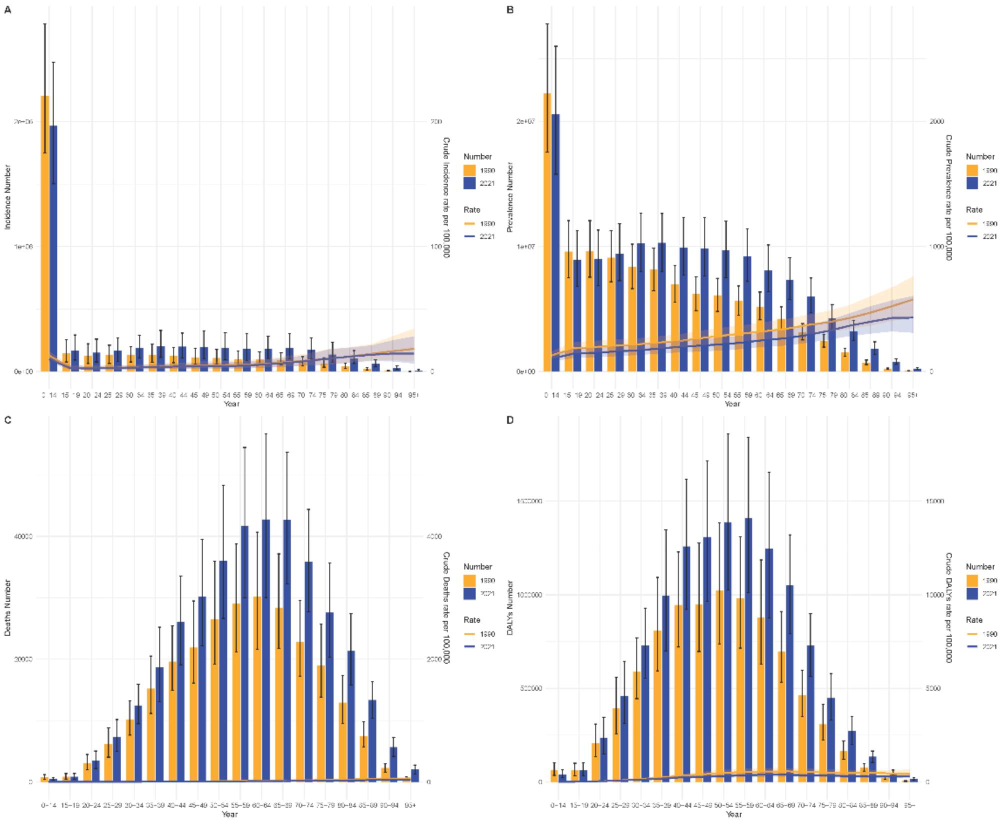

Fig.S13c Global Age-specific incidence, prevalence, deaths, and DALYs of Cirrhosis due to hepatitis C, 1990 and 2021

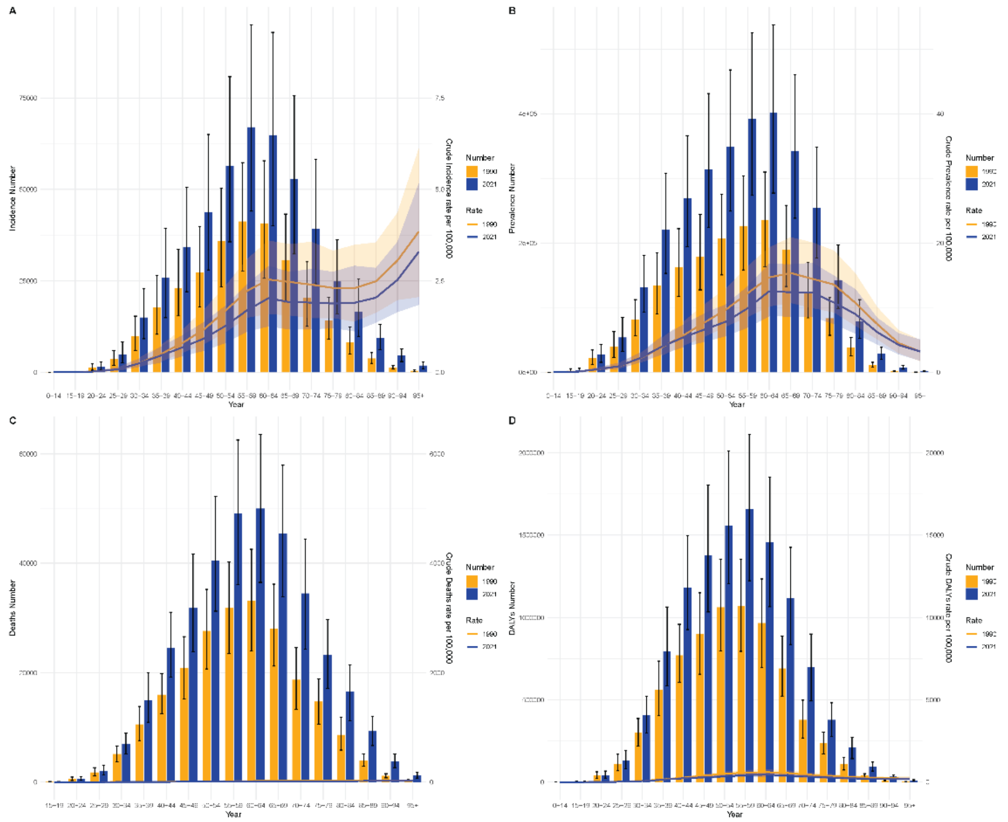

Fig.S13d Global Age-specific incidence, prevalence, deaths, and DALYs of Cirrhosis due to alcohol, 1990 and 2021

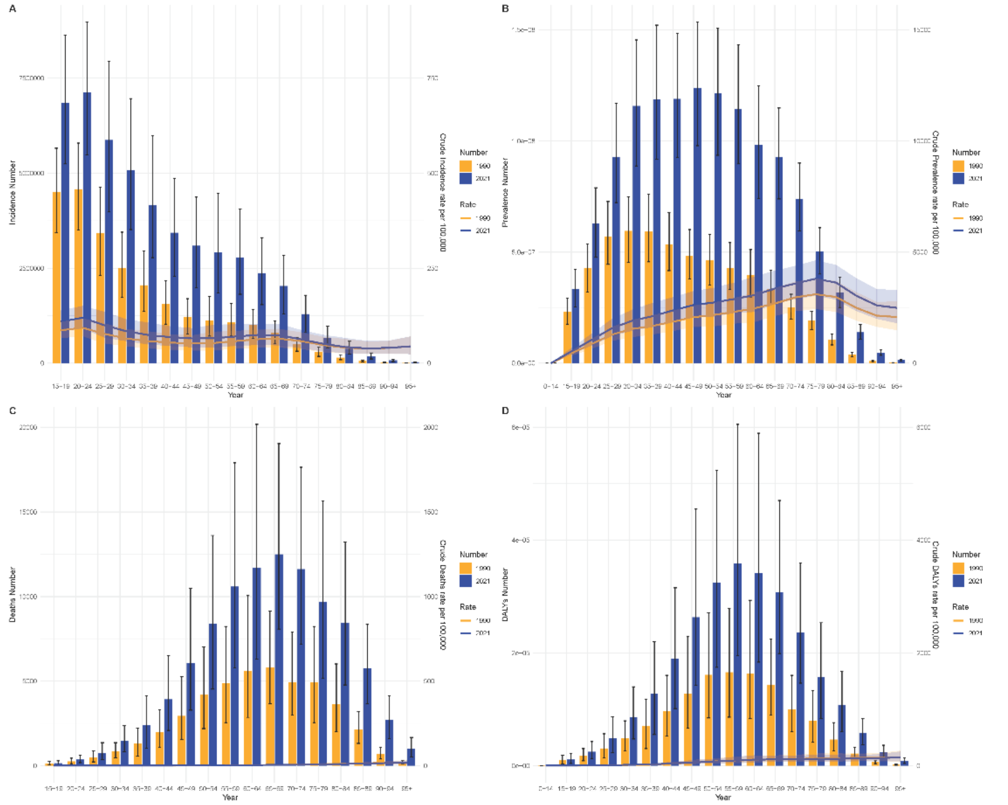

Fig.S13e Global Age-specific incidence, prevalence, deaths, and DALYs of Cirrhosis due to NAFLD, 1990 and 2021

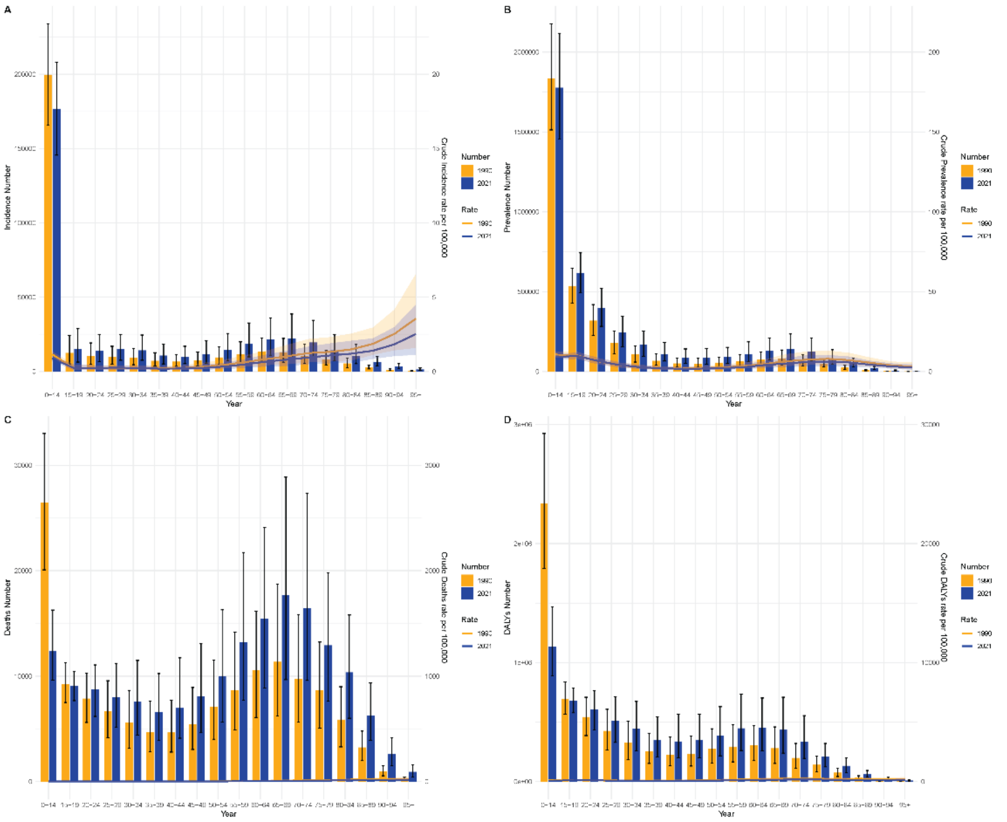

Fig.S13f Global Age-specific incidence, prevalence, deaths, and DALYs of Cirrhosis due to other causes, 1990 and 2021

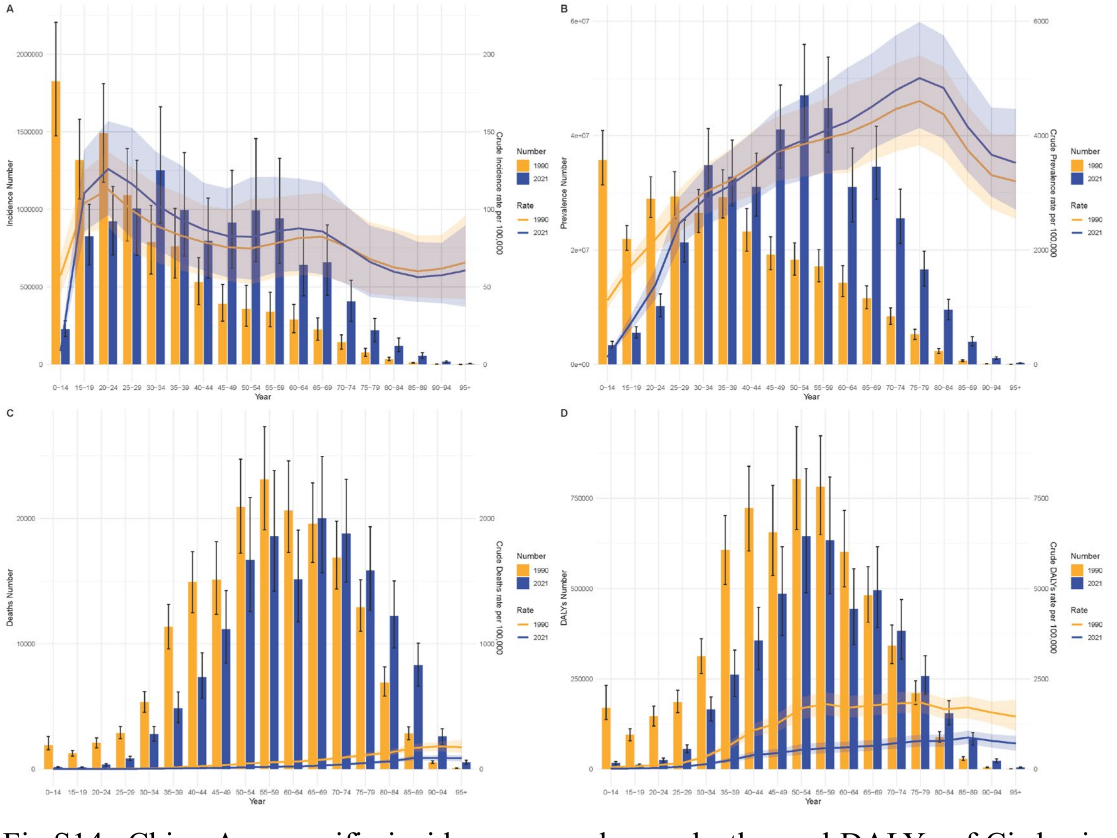

Fig.S14a China Age-specific incidence, prevalence, deaths, and DALYs of Cirrhosis, 1990 and 2021

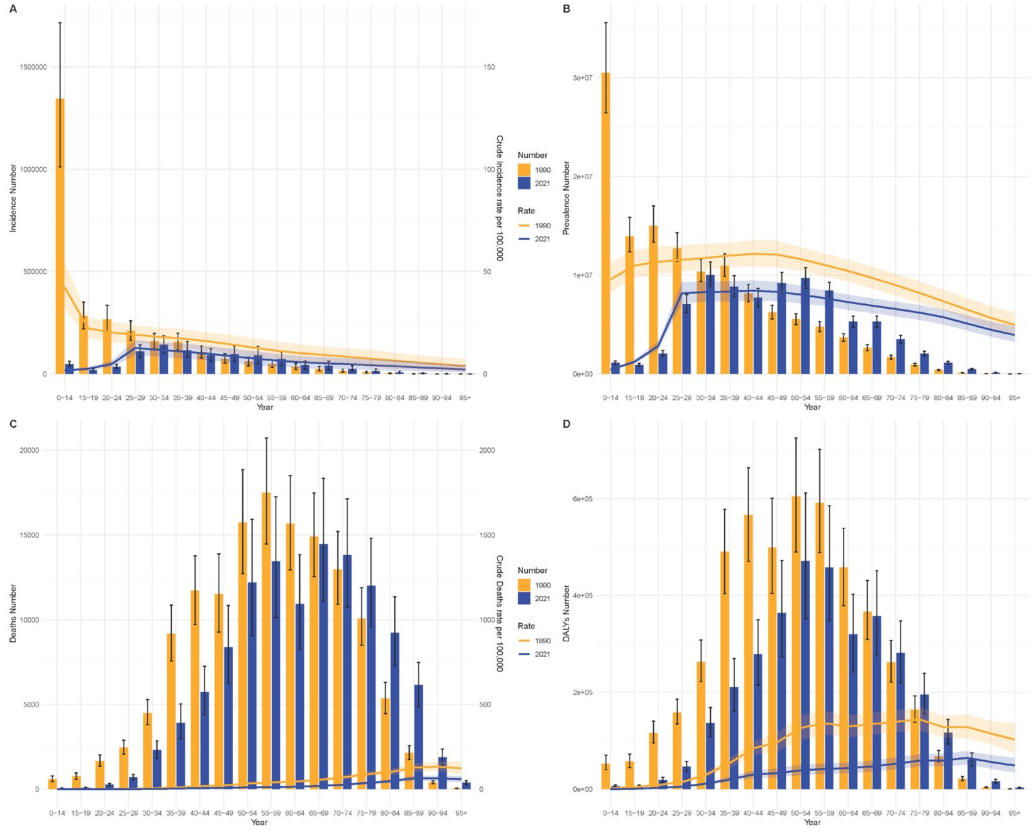

Fig.S14b China Age-specific incidence, prevalence, deaths, and DALYs of Cirrhosis due to hepatitis B, 1990 and 2021

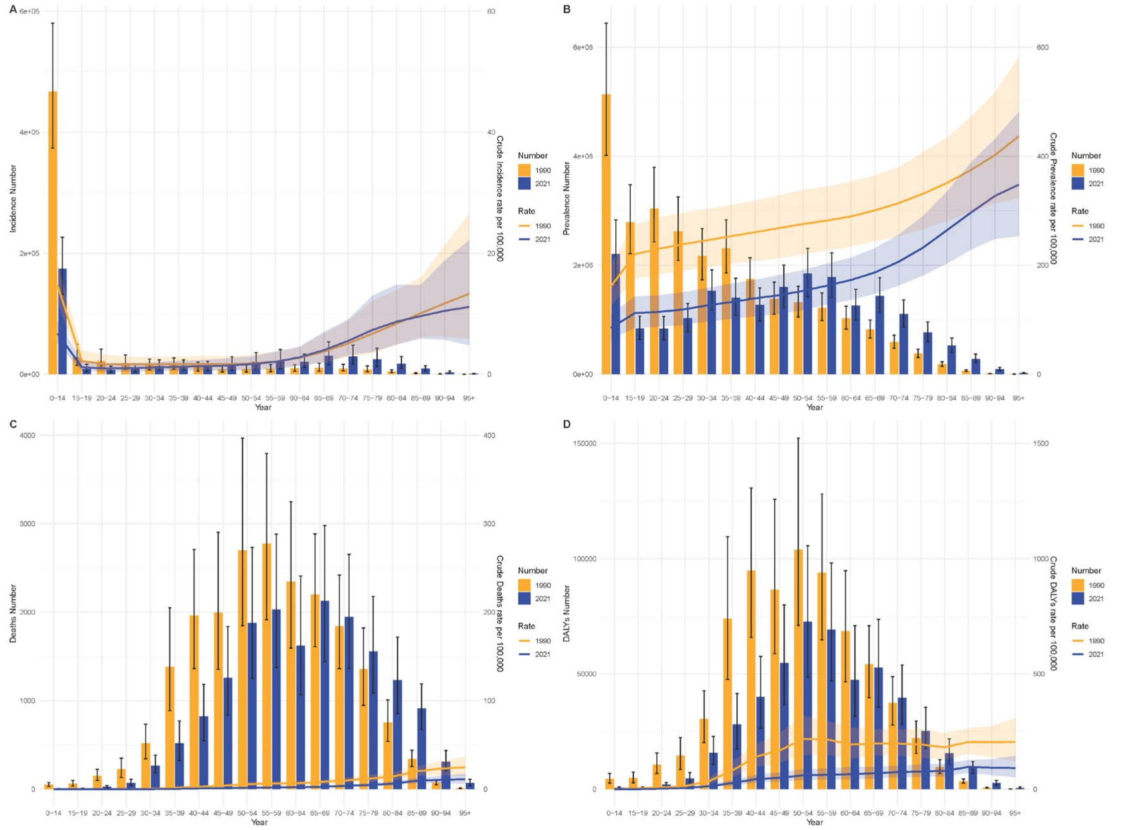

Fig.S14c China Age-specific incidence, prevalence, deaths, and DALYs of Cirrhosis

due to hepatitis C, 1990 and 2021

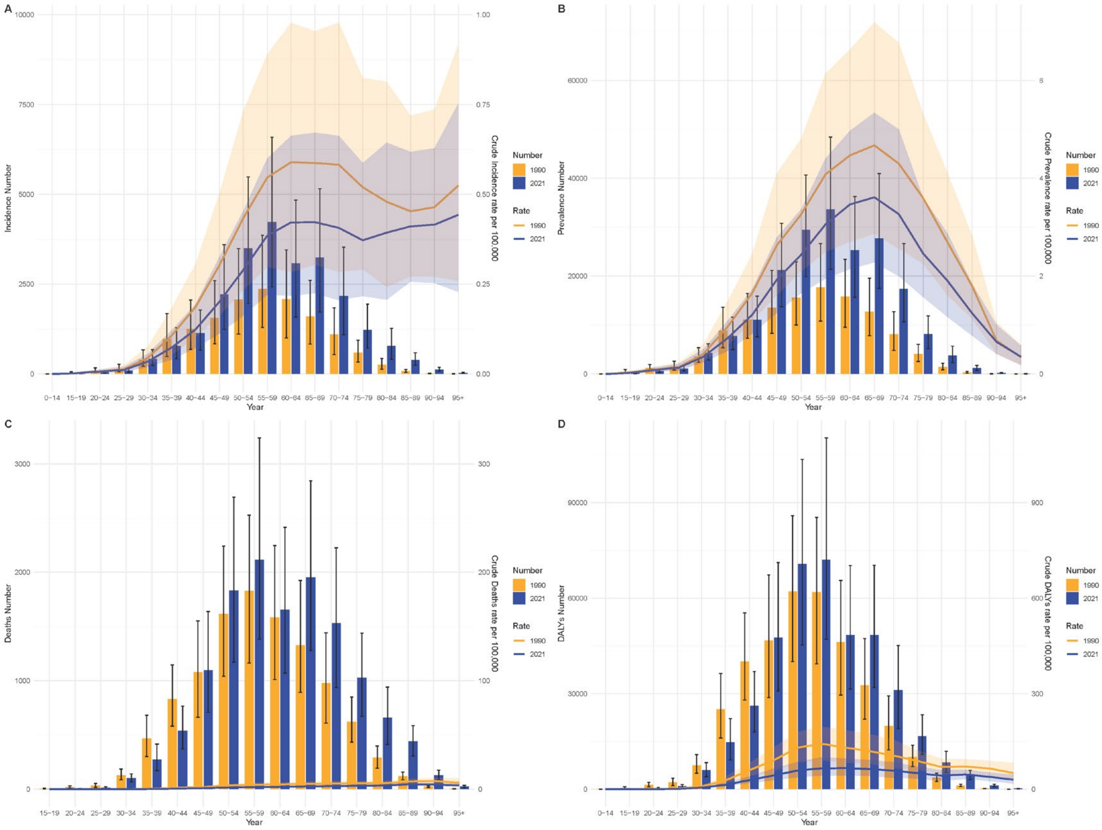

Fig.S14d China Age-specific incidence, prevalence, deaths, and DALYs of Cirrhosis due to alcohol, 1990 and 2021

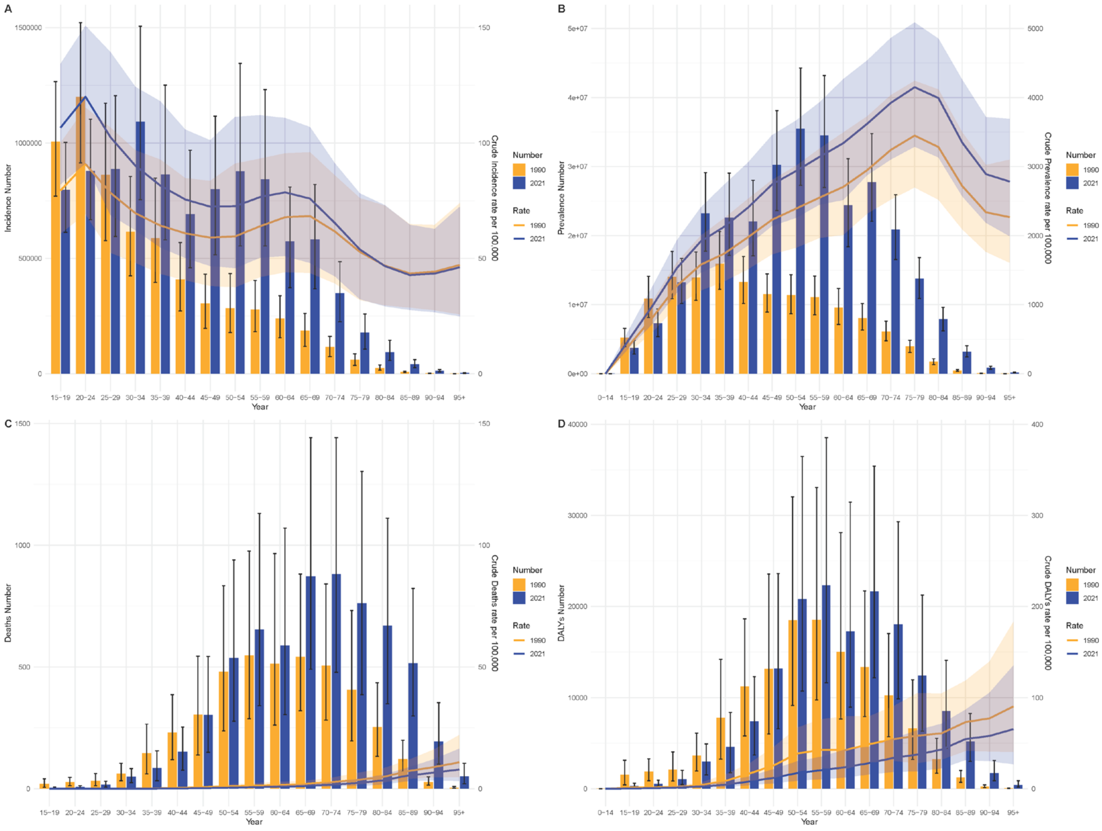

Fig.S14e China Age-specific incidence, prevalence, deaths, and DALYs of Cirrhosis

due to NAFLD, 1990 and 2021

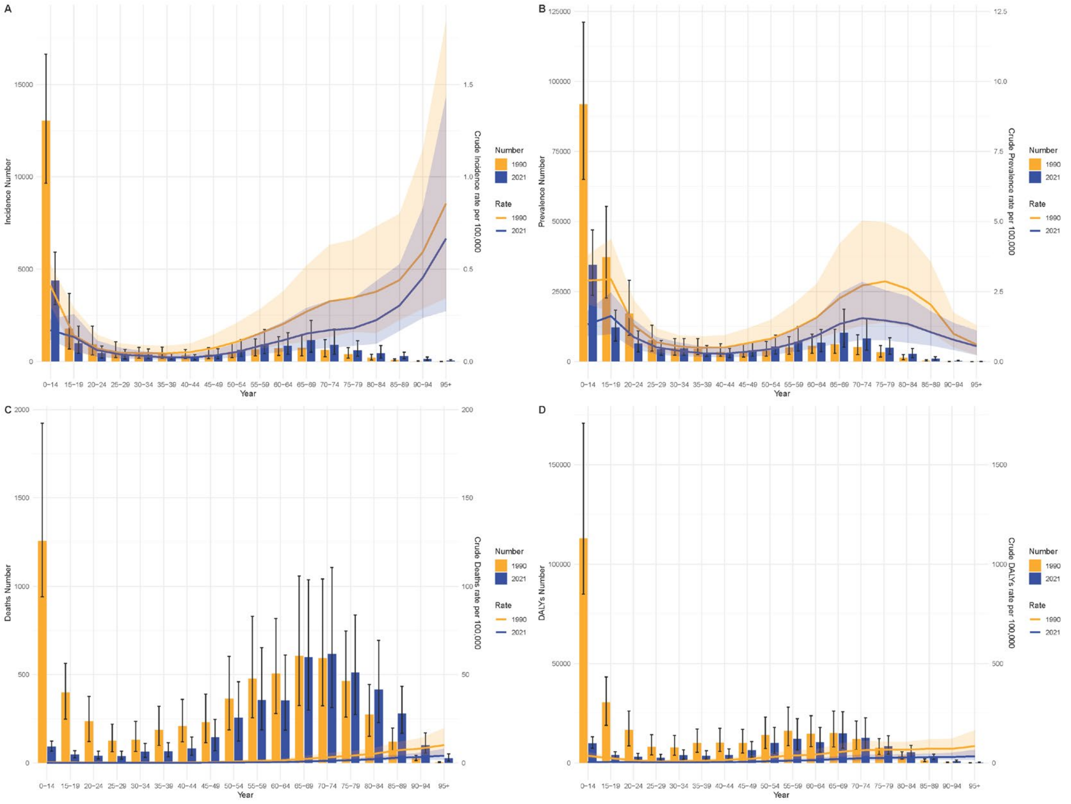

Fig.S14f China Age-specific incidence, prevalence, deaths, and DALYs of Cirrhosis due to other causes, 1990 and 2021

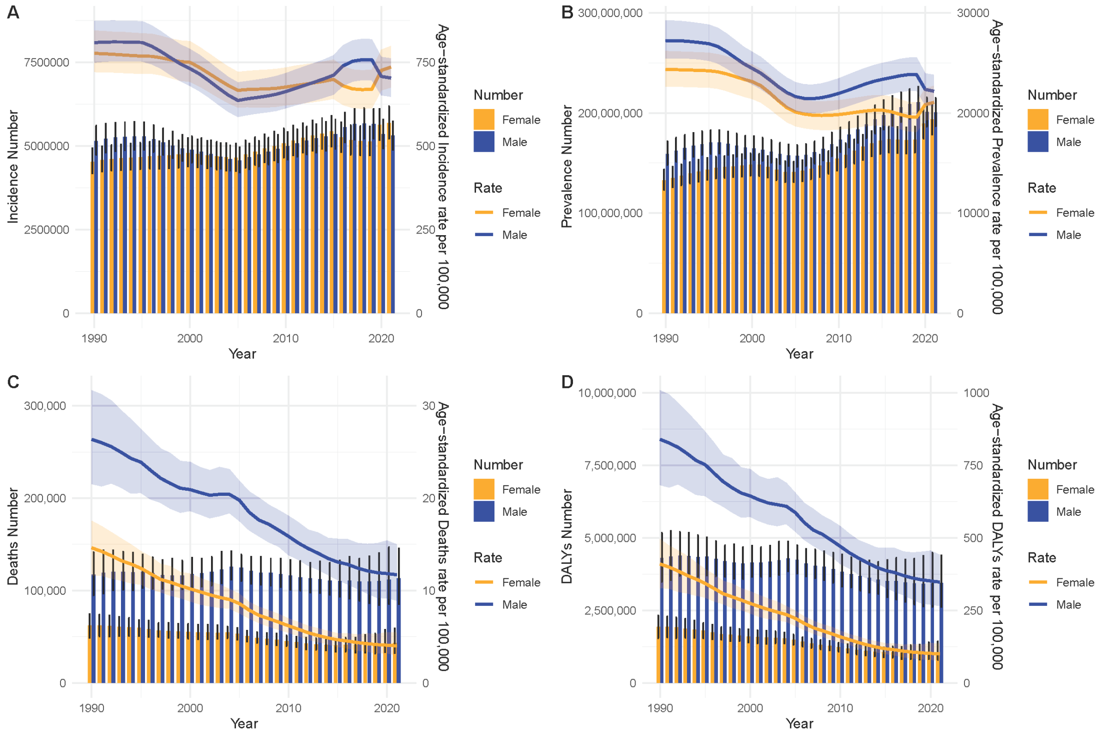

Fig.S15a Trends in incidence, prevalence, deaths, and DALYs of cirrhosis by sex in China, 1990–2021.

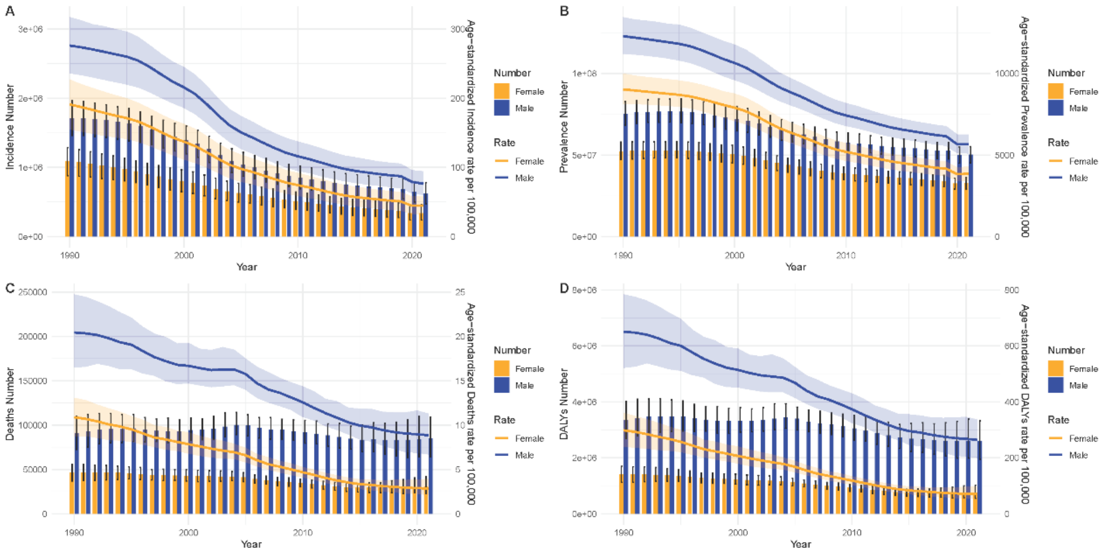

Fig.S15b Trends in incidence, prevalence, deaths, and DALYs of cirrhosis due to hepatitis B by sex in China, 1990–2021.

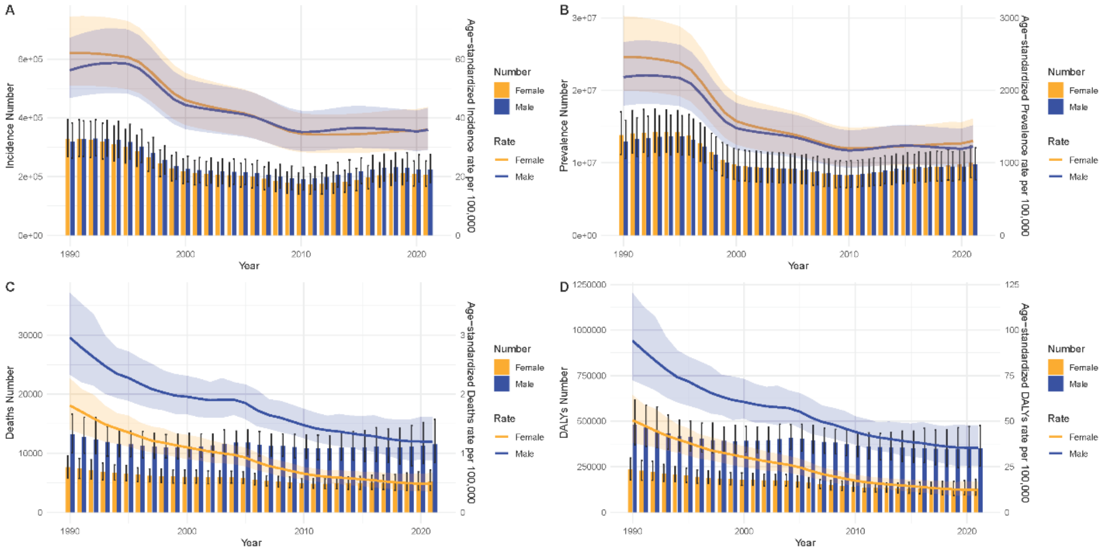

Fig.S15c Trends in incidence, prevalence, deaths, and DALYs of cirrhosis due to hepatitis C by sex in China, 1990–2021.

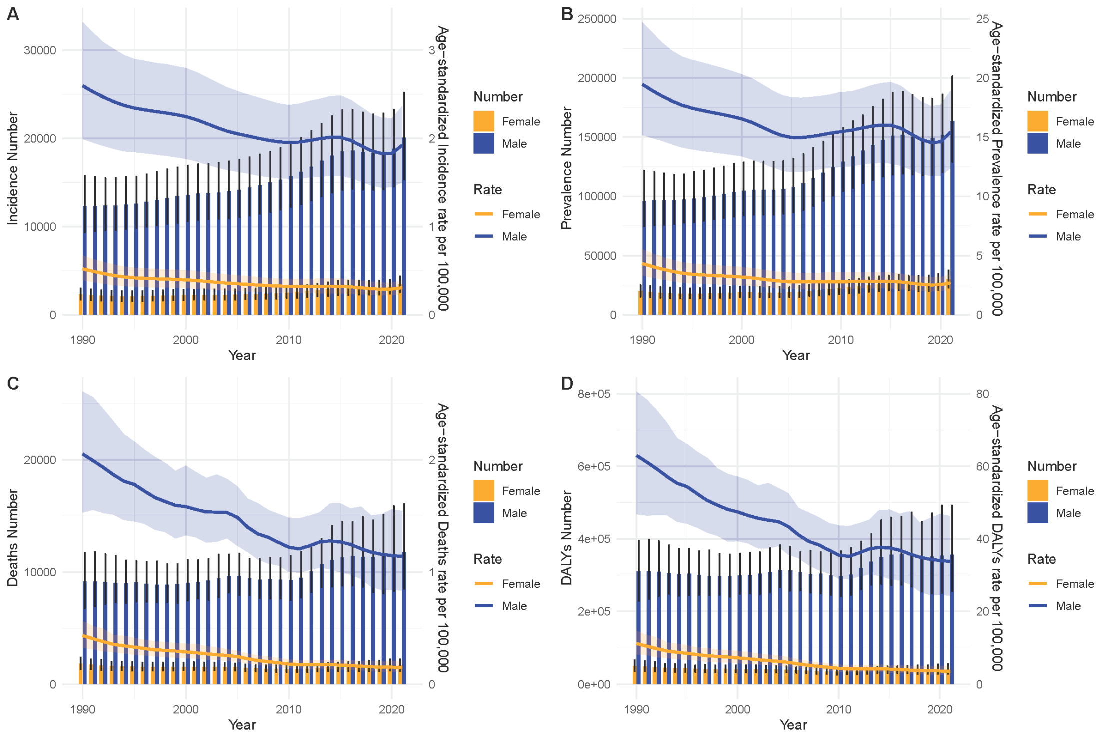

Fig.S15d Trends in incidence, prevalence, deaths, and DALYs of cirrhosis due to alcohol by sex in China, 1990–2021.

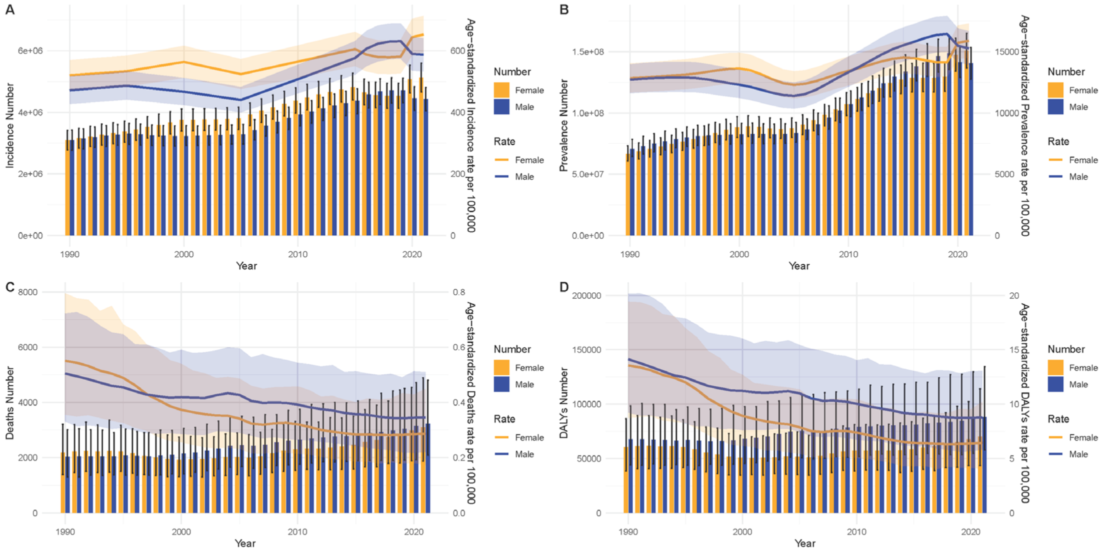

Fig.S15e Trends in incidence, prevalence, deaths, and DALYs of cirrhosis due to NAFLD by sex in China, 1990–2021.

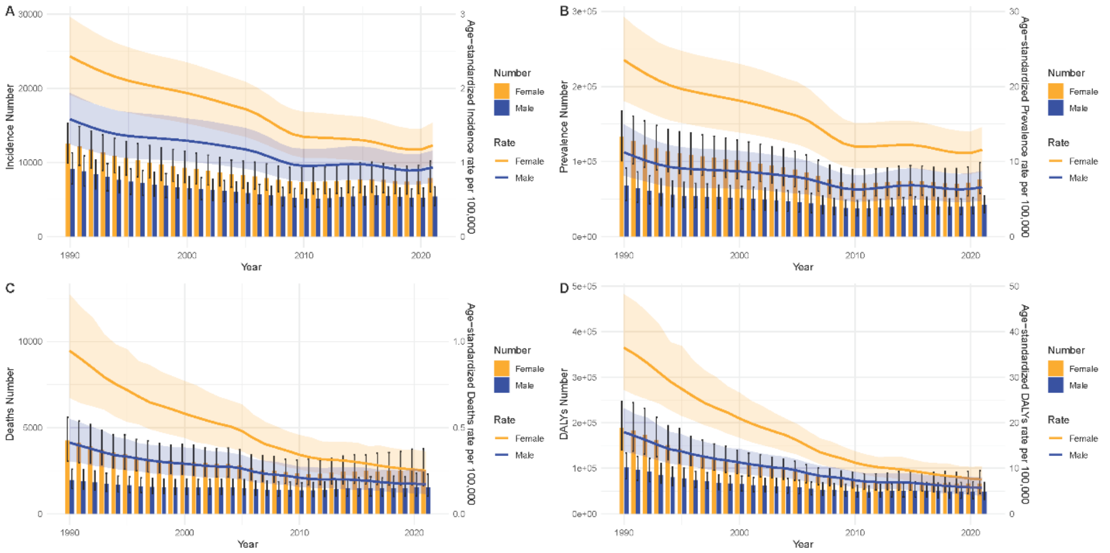

Fig.S15f Trends in incidence, prevalence, deaths, and DALYs of cirrhosis due to other causes by sex in China, 1990–2021.

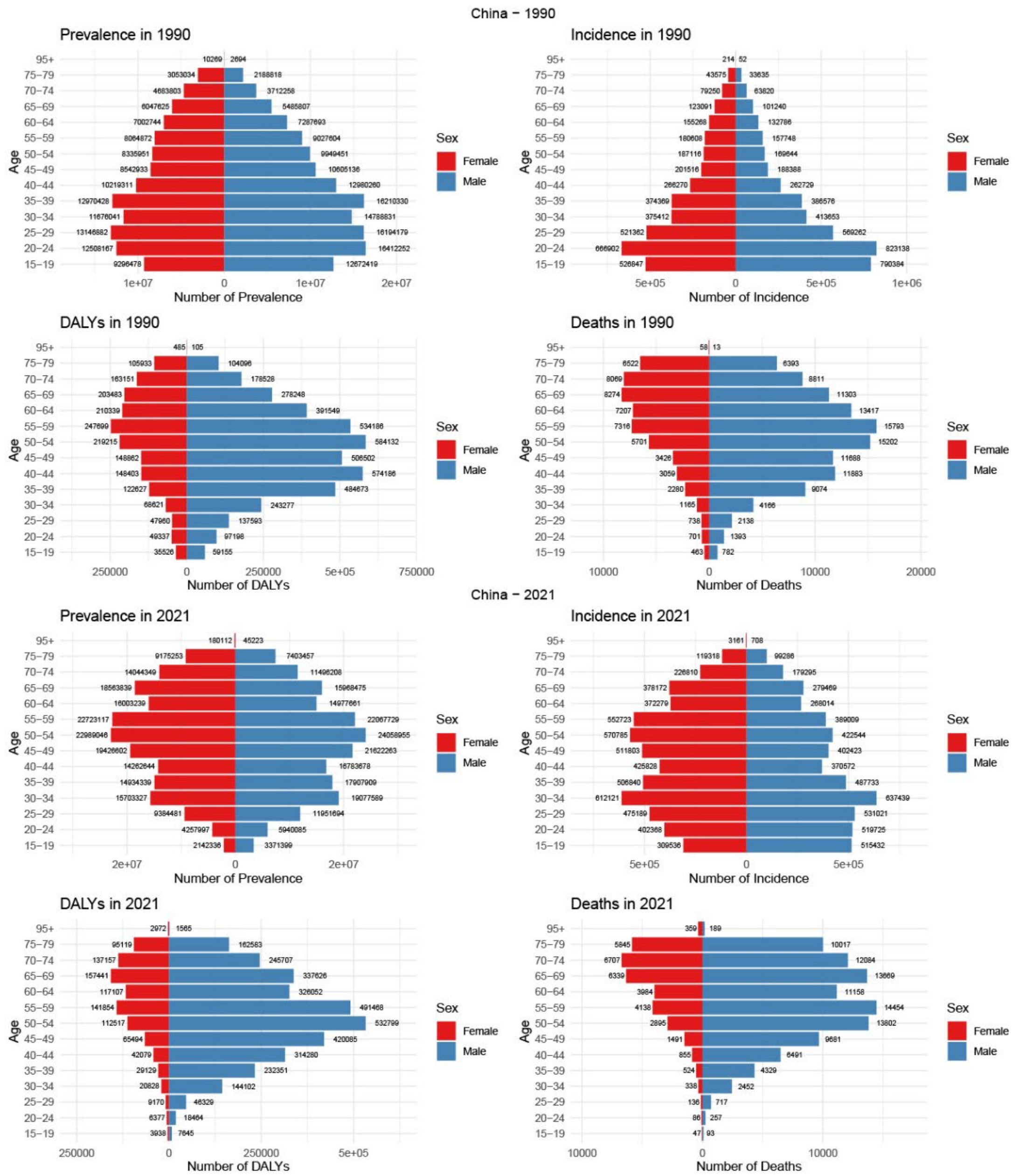

Fig.S16a Age–Sex Distribution of Cirrhosis Burden in China, 1990 and 2021.

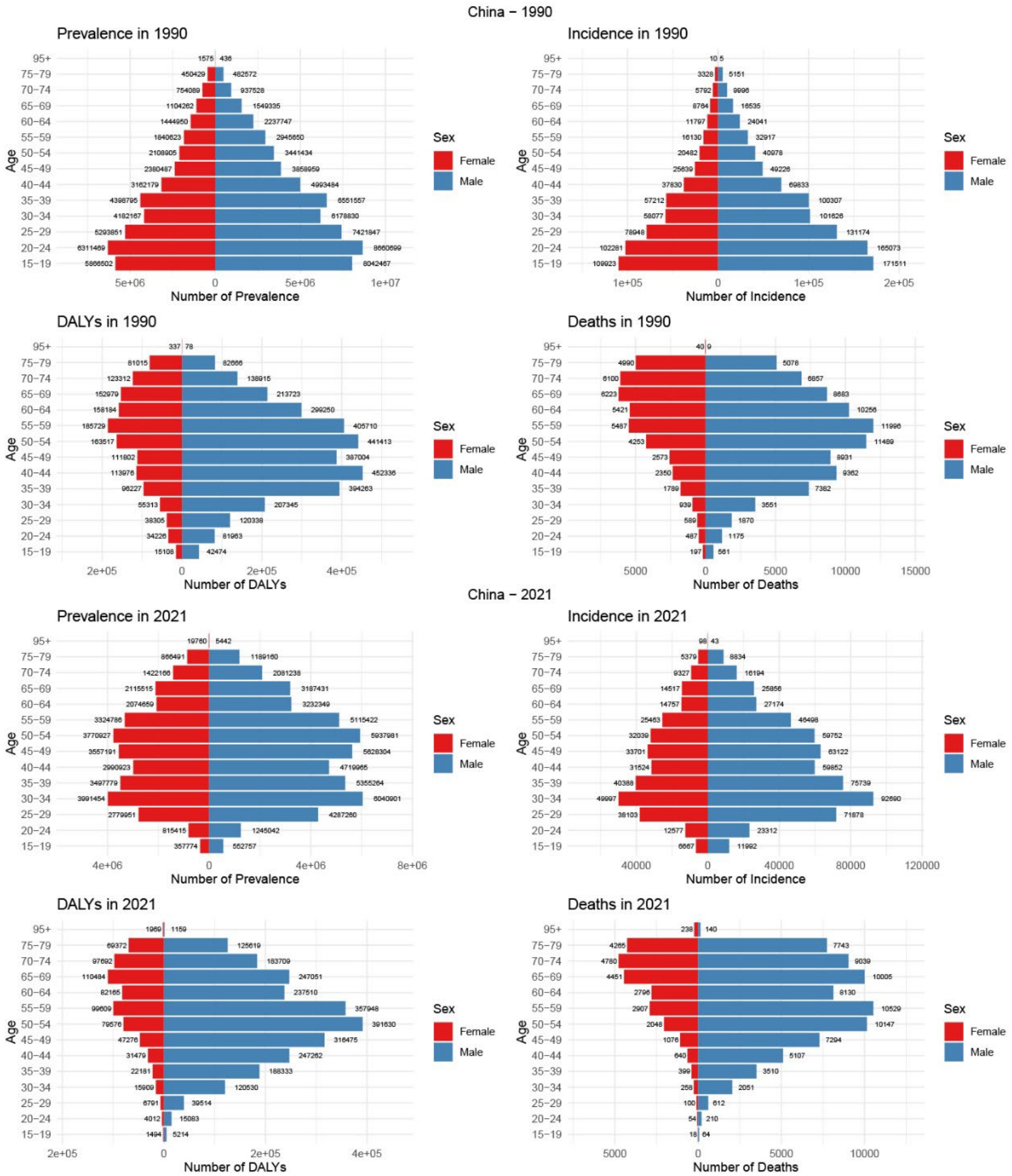

Fig.S16b Age–Sex Distribution of Cirrhosis due to hepatitis B Burden in China, 1990 and 2021.

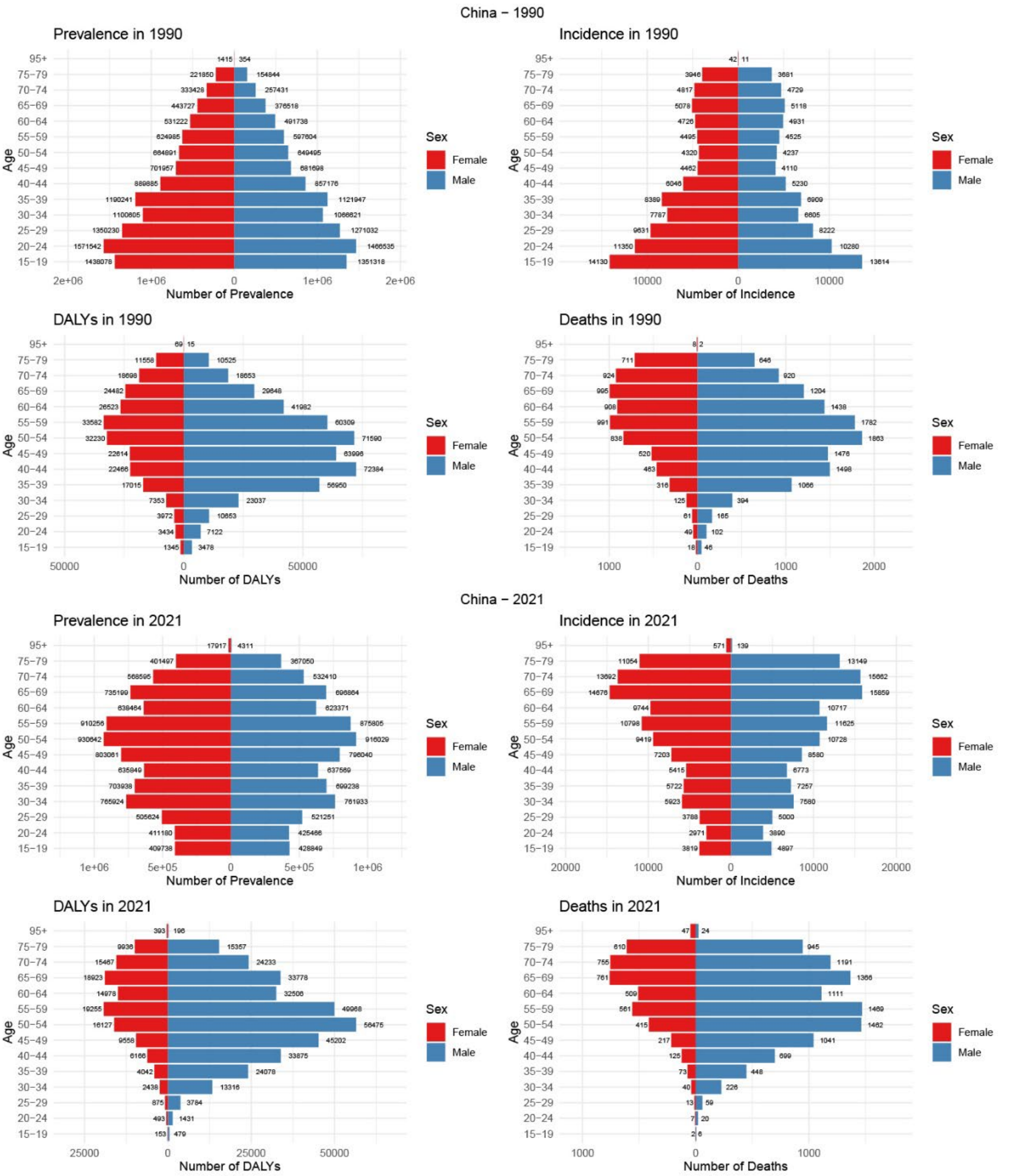

Fig.S16c Age–Sex Distribution of Cirrhosis due to hepatitis C Burden in China, 1990 and 2021.

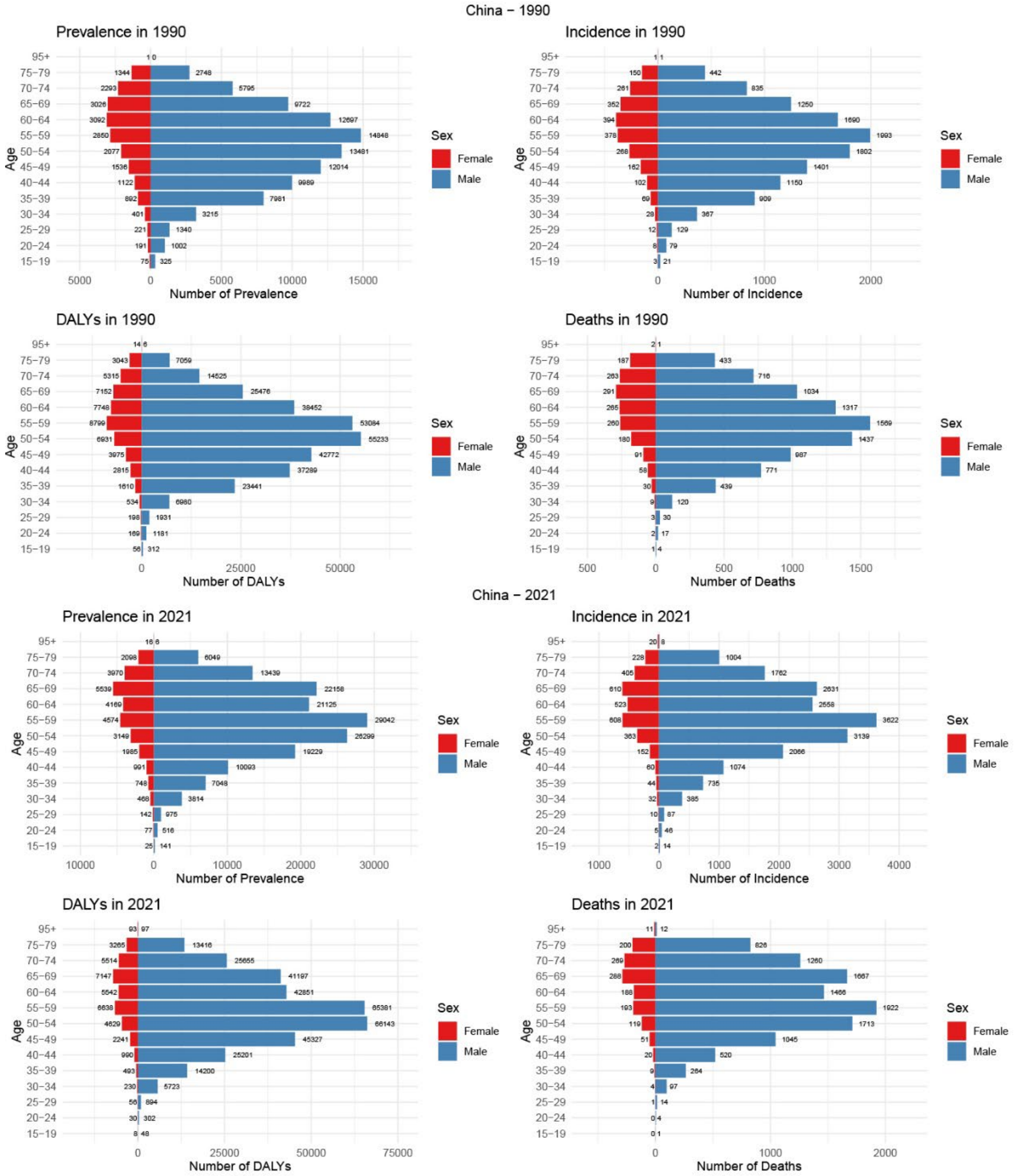

Fig.S16d Age–Sex Distribution of Cirrhosis due to alcohol Burden in China, 1990 and 2021.

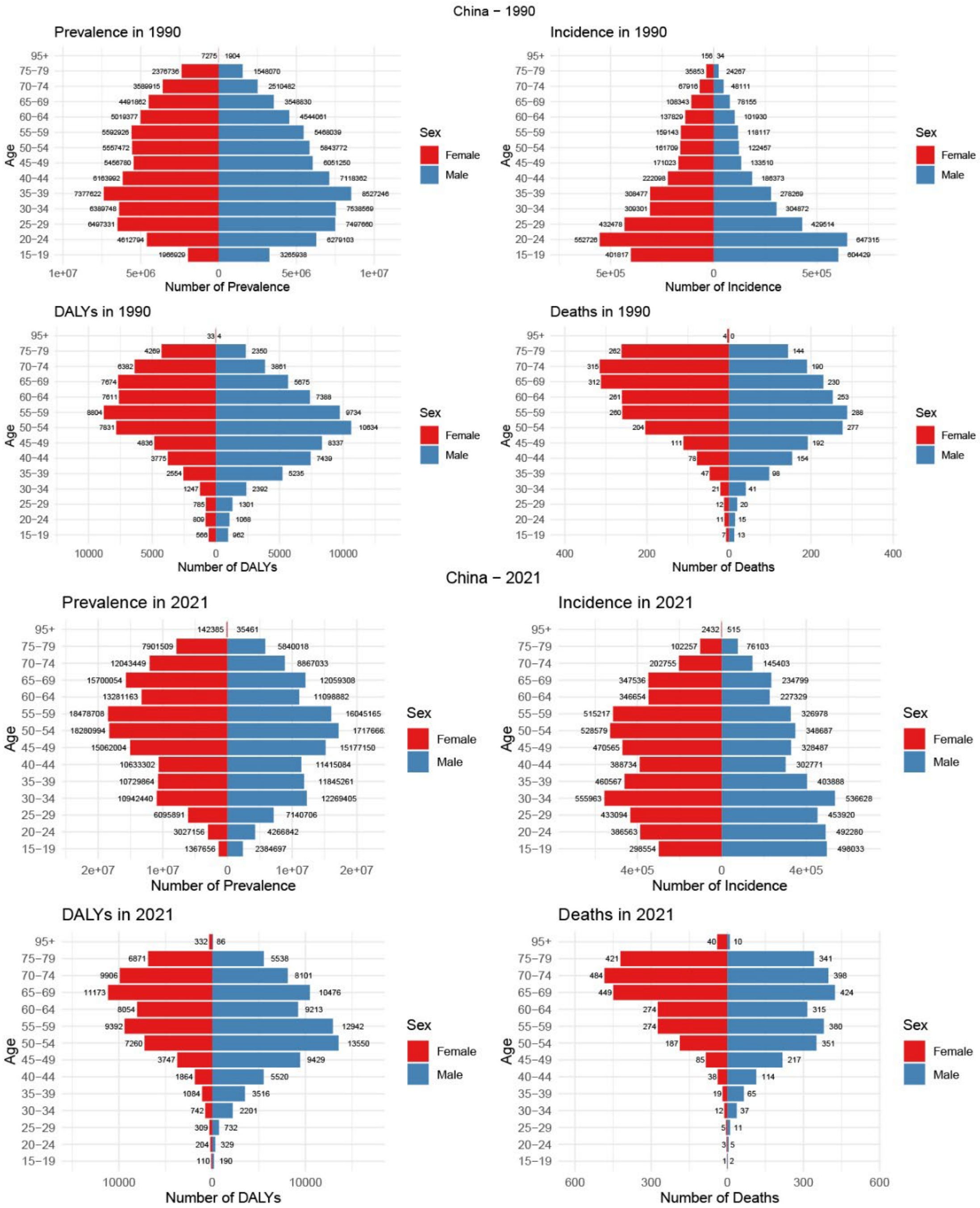

Fig.S16e Age–Sex Distribution of Cirrhosis due to NAFLD Burden in China, 1990 and 2021.

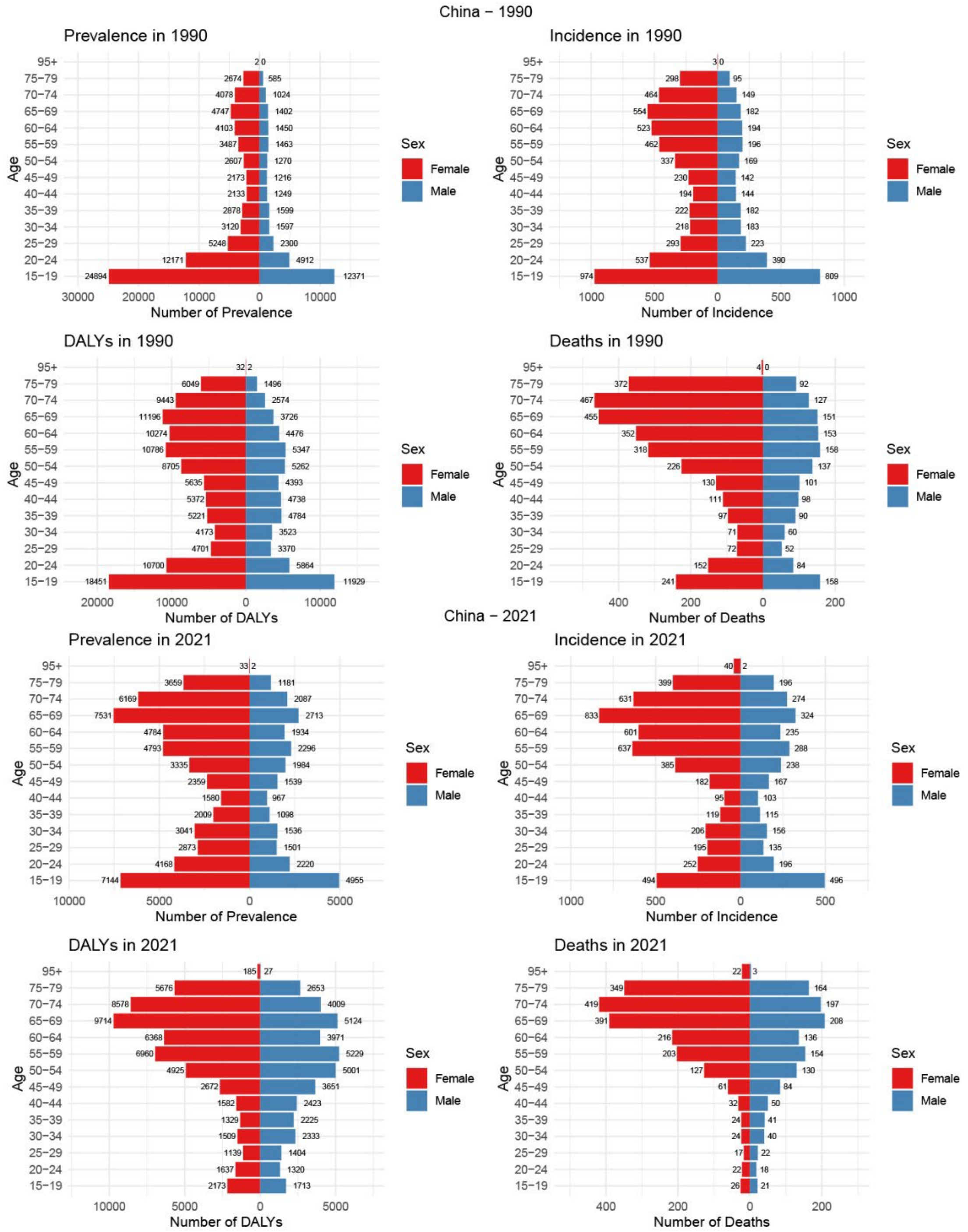

Fig.S16f Age–Sex Distribution of Cirrhosis due to other causes Burden in China, 1990 and 2021.

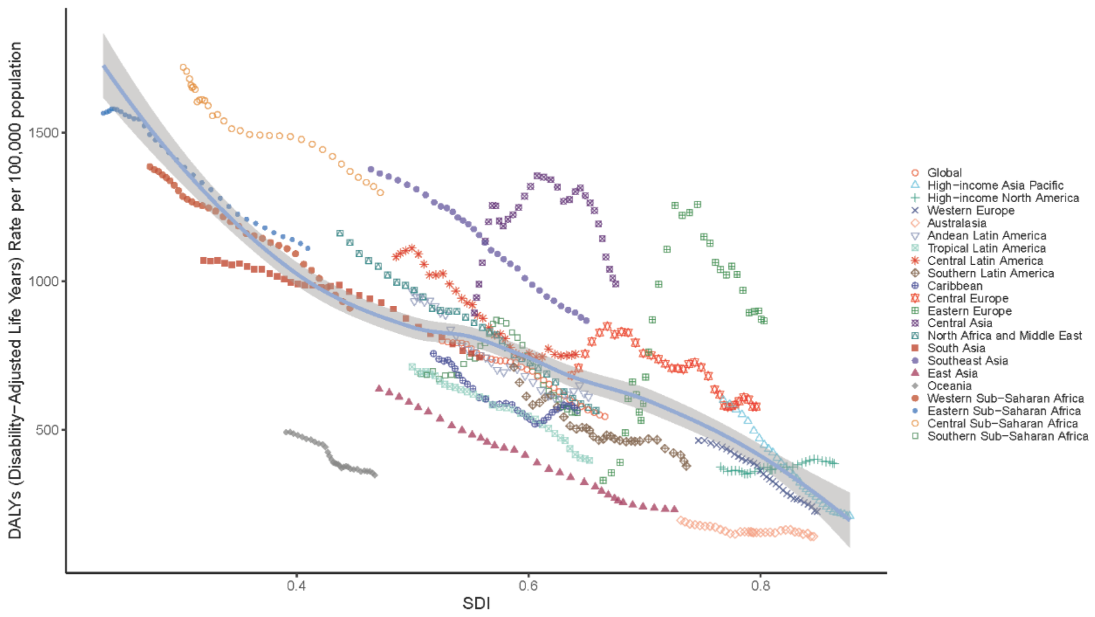

Fig.S17a Age-standardized DALYS rates of cirrhosis, globally and for 21 GBD regions, by SDI, 1990–2021

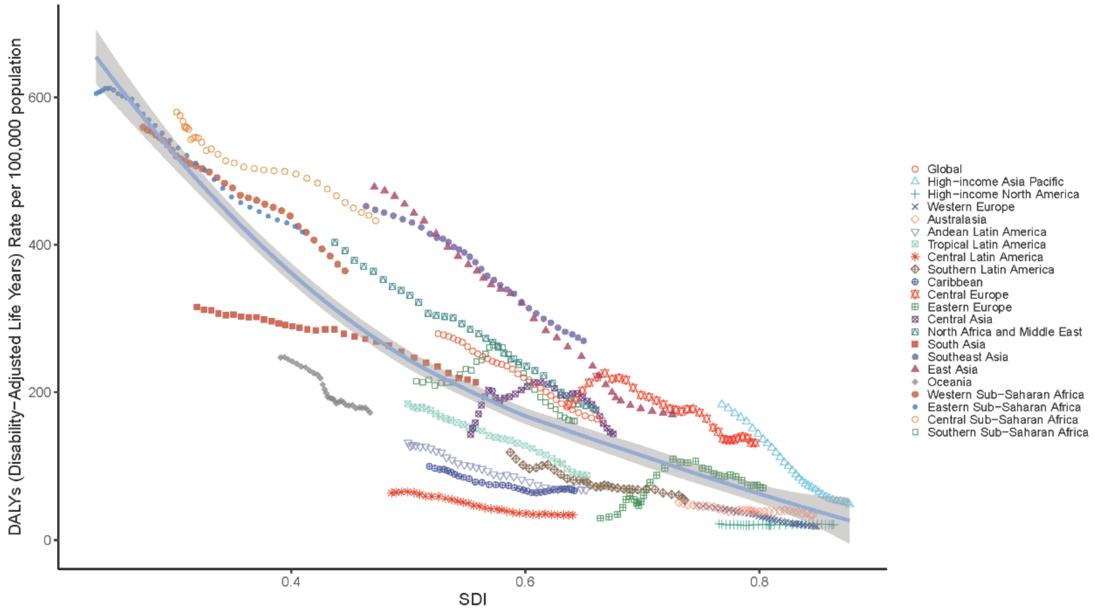

Fig.S17b Age-standardized DALYS rates of cirrhosis due to hepatitis B, globally and for 21 GBD regions, by SDI, 1990–2021

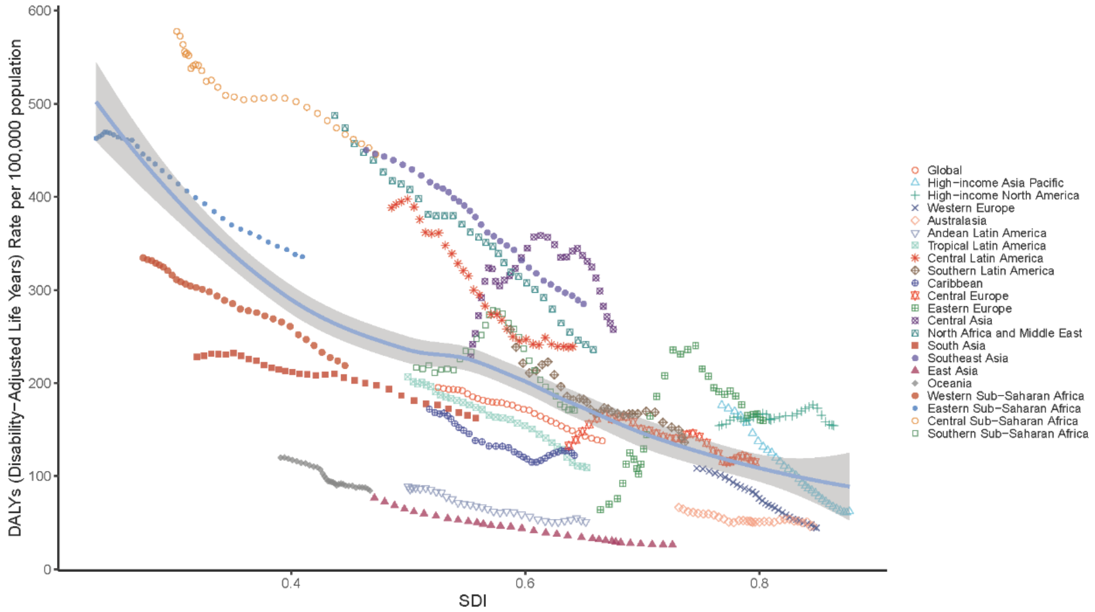

Fig.S17c Age-standardized DALYS rates of cirrhosis due to hepatitis C, globally and for 21 GBD regions, by SDI, 1990–2021

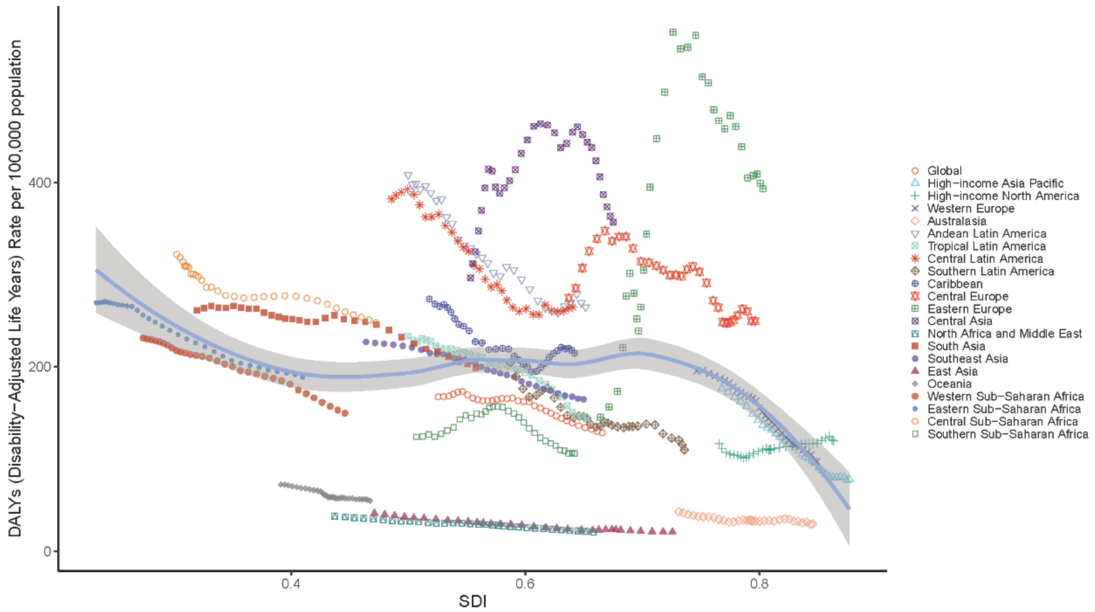

Fig.S17d Age-standardized DALYS rates of cirrhosis due to alcohol, globally and for 21 GBD regions, by SDI, 1990–2021

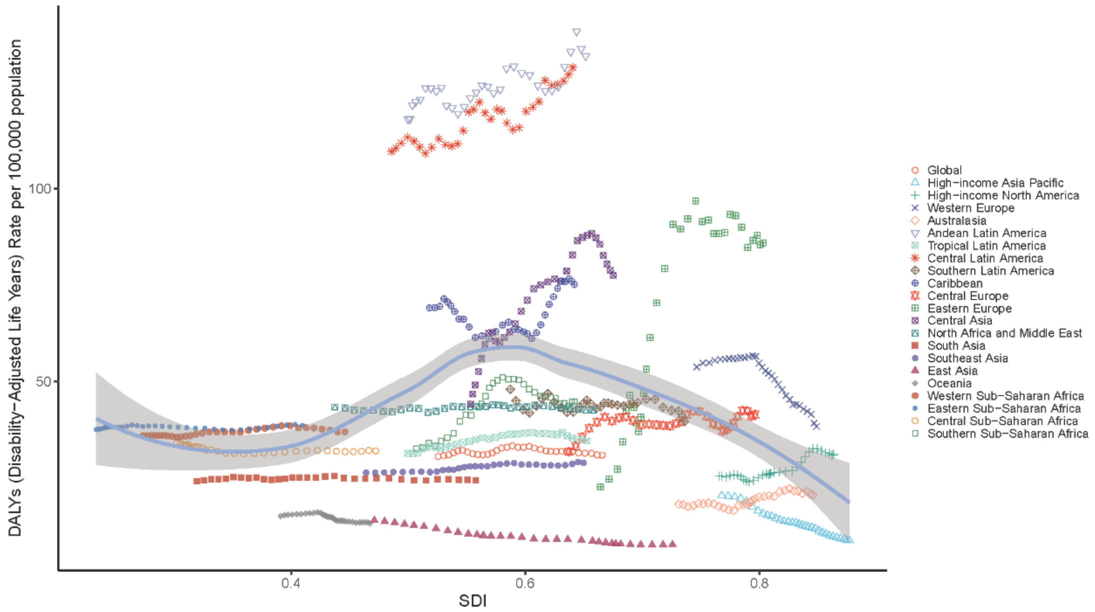

Fig.S17e Age-standardized DALYS rates of cirrhosis due to NAFLD, globally and for 21 GBD regions, by SDI, 1990–2021

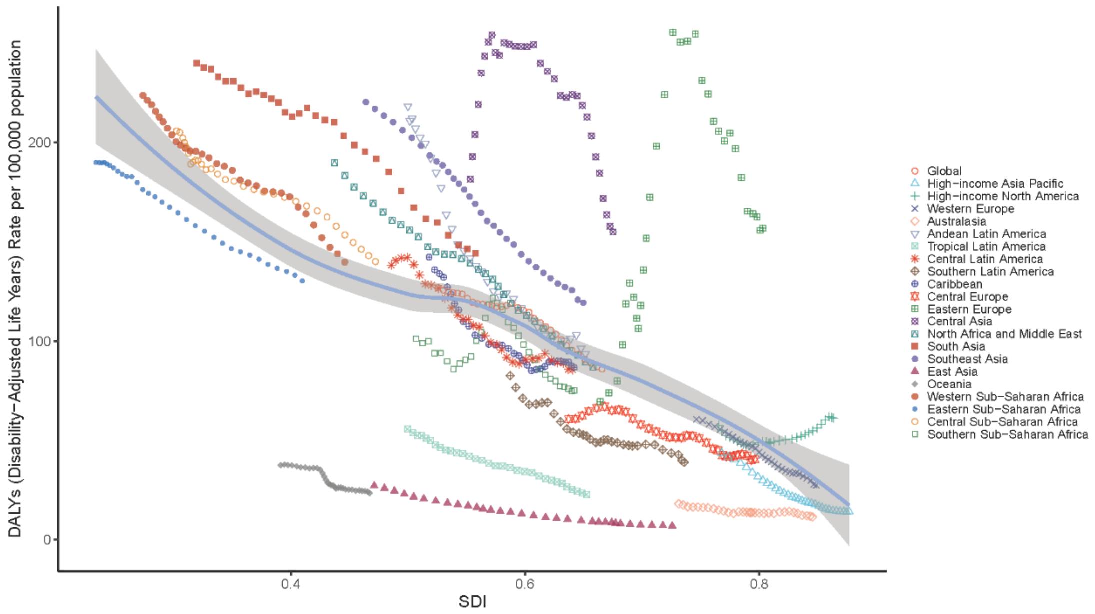

Fig.S17f Age-standardized DALYS rates of cirrhosis due to other causes, globally and for 21 GBD regions, by SDI, 1990–2021

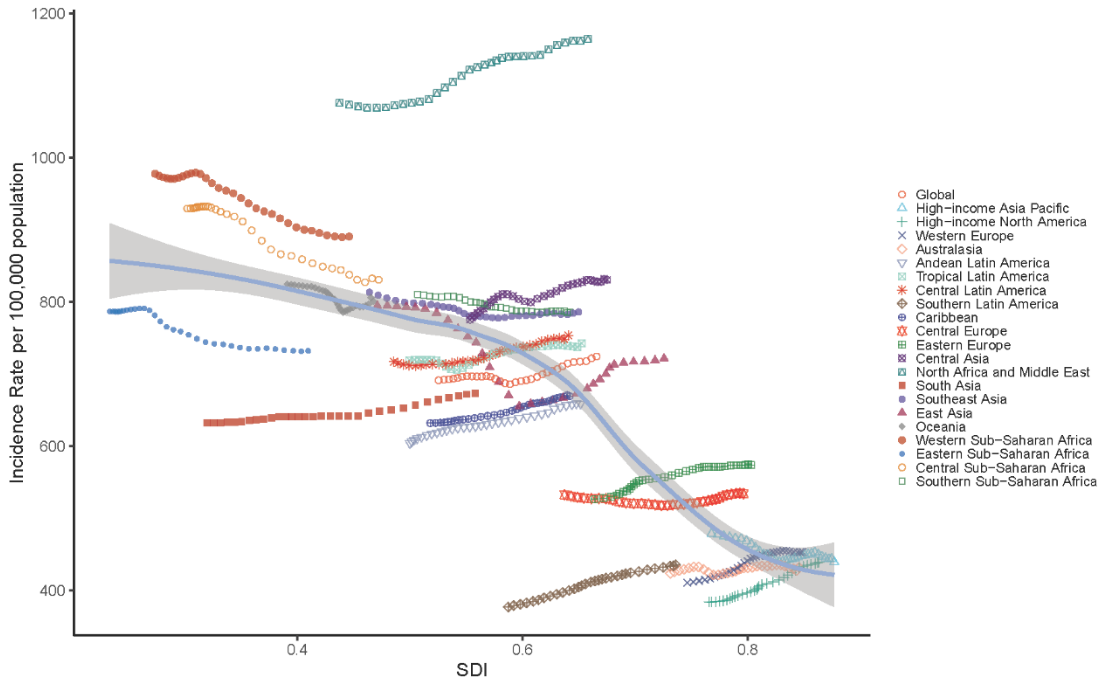

Fig.S18a Age-standardized Incidence rates of cirrhosis, globally and for 21 GBD regions, by SDI, 1990–2021

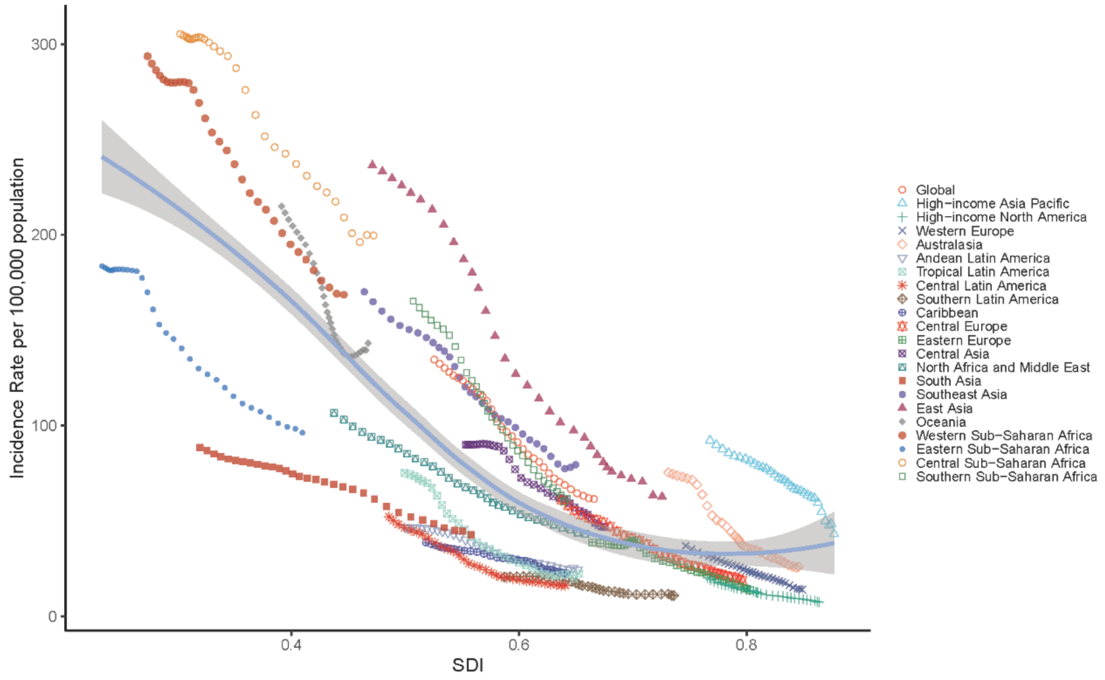

Fig.S18b Age-standardized Incidence rates of cirrhosis due to hepatitis B, globally and for 21 GBD regions, by SDI, 1990–2021

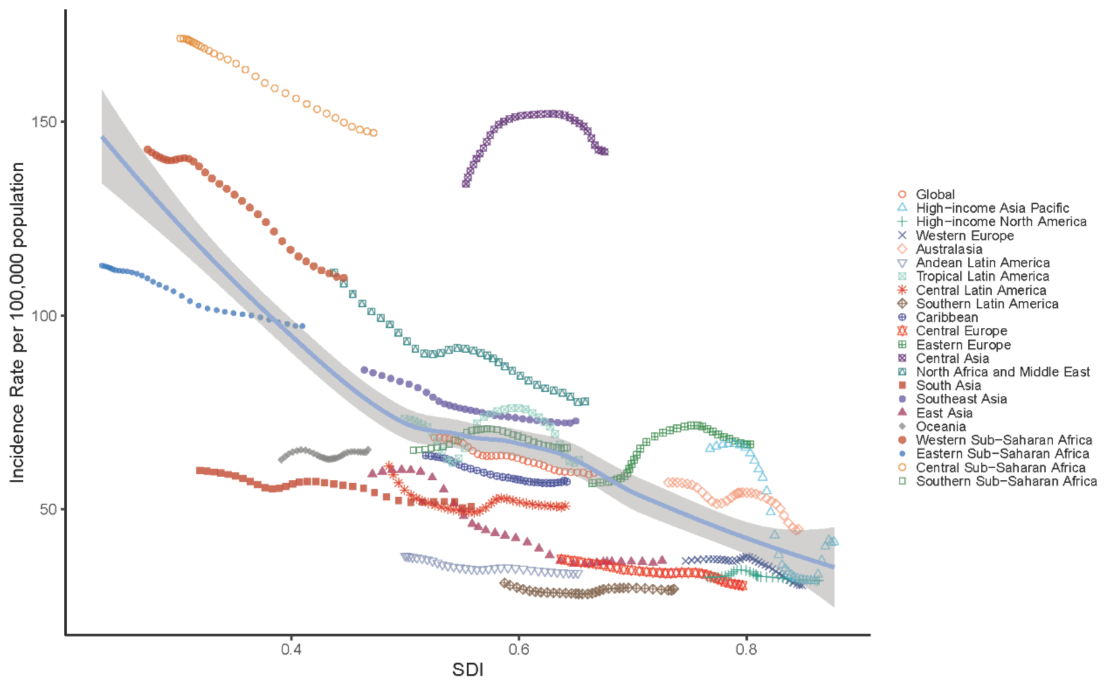

Fig.S18c Age-standardized Incidence rates of cirrhosis due to hepatitis C, globally and for 21 GBD regions, by SDI, 1990–2021

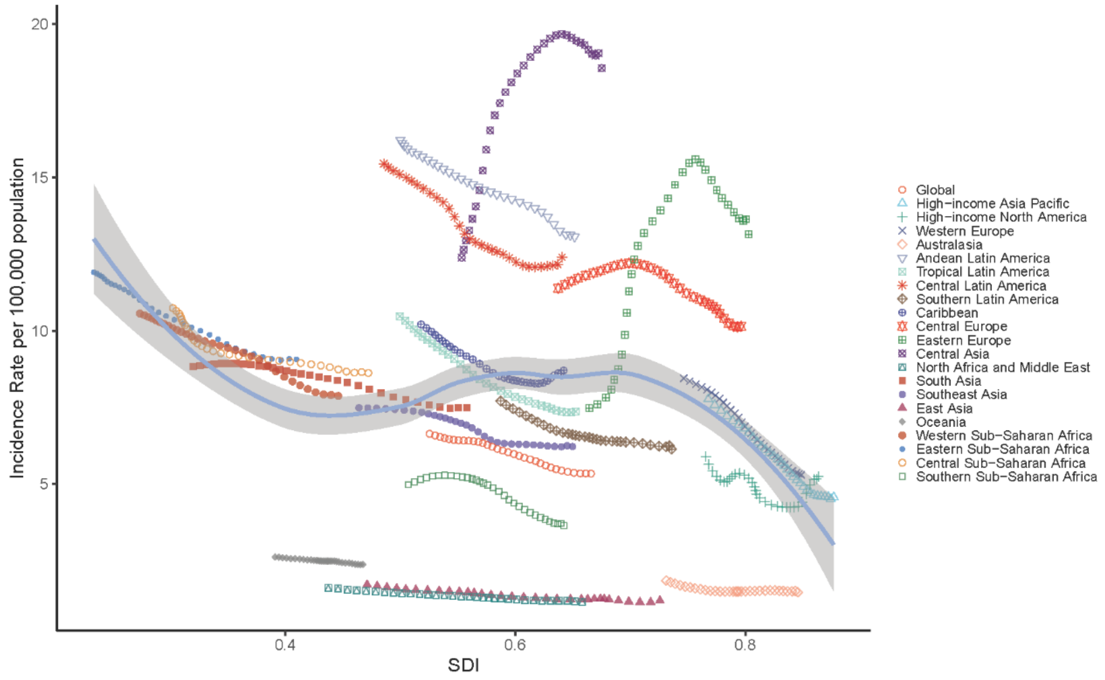

Fig.S18d Age-standardized Incidence rates of cirrhosis due to alcohol, globally and for 21 GBD regions, by SDI, 1990–2021

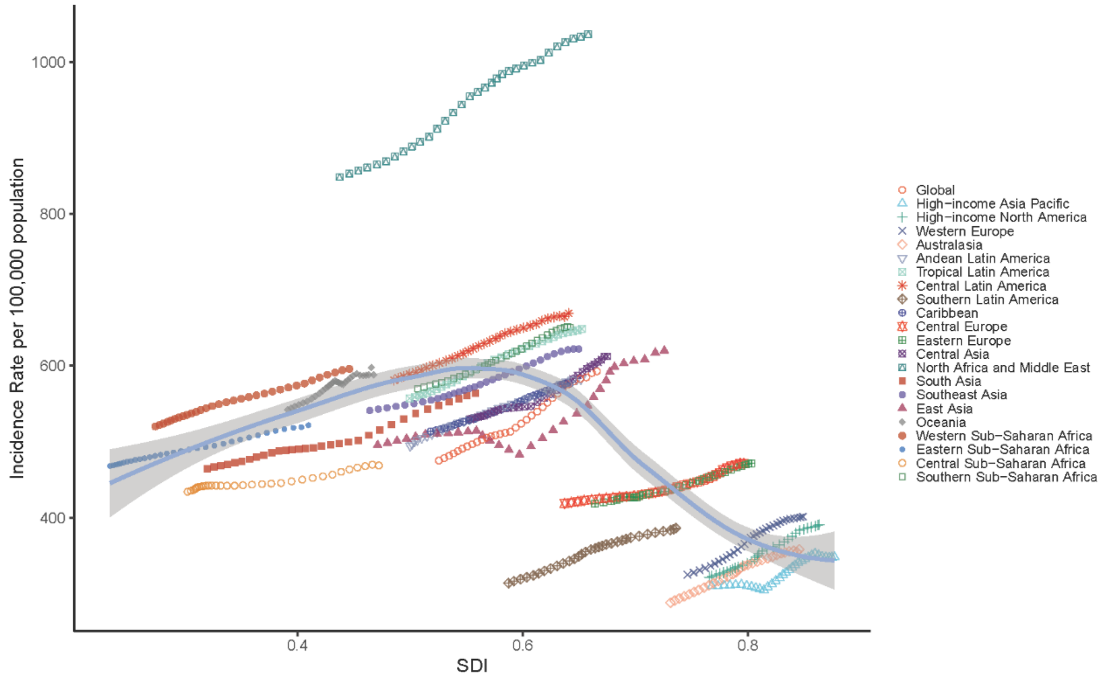

Fig.S18e Age-standardized Incidence rates of cirrhosis due to NAFLD, globally and for 21 GBD regions, by SDI, 1990–2021

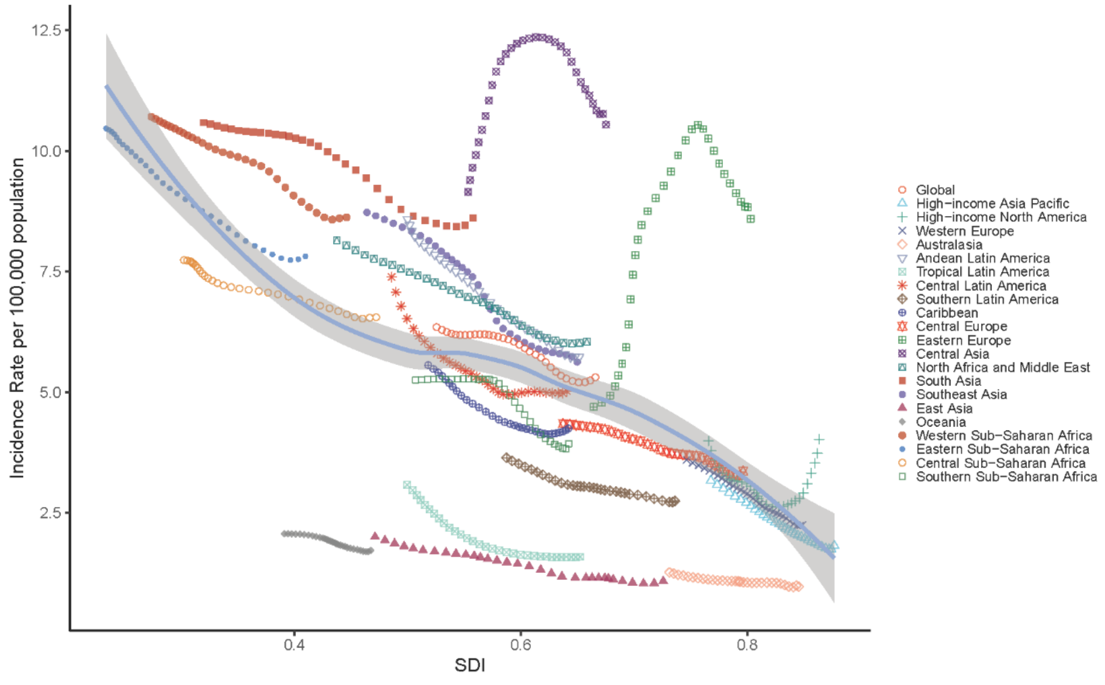

Fig.S18f Age-standardized Incidence rates of cirrhosis due to other causes, globally and for 21 GBD regions, by SDI, 1990–2021

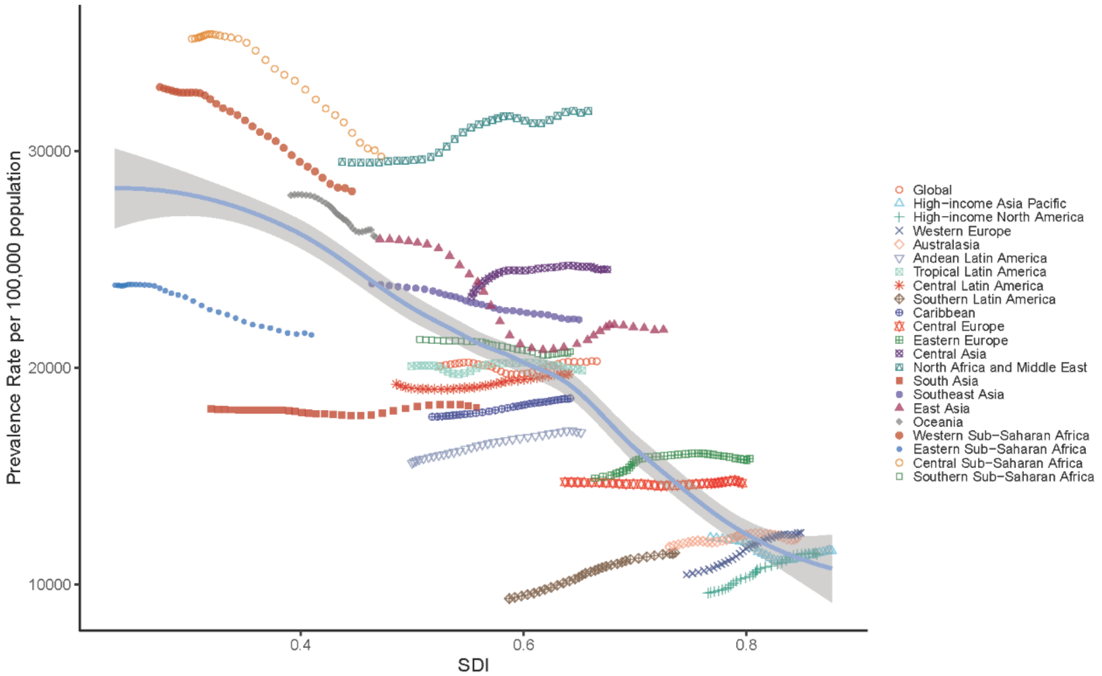

Fig.S19a Age-standardized Prevalence rates of cirrhosis, globally and for 21 GBD regions, by SDI, 1990–2021

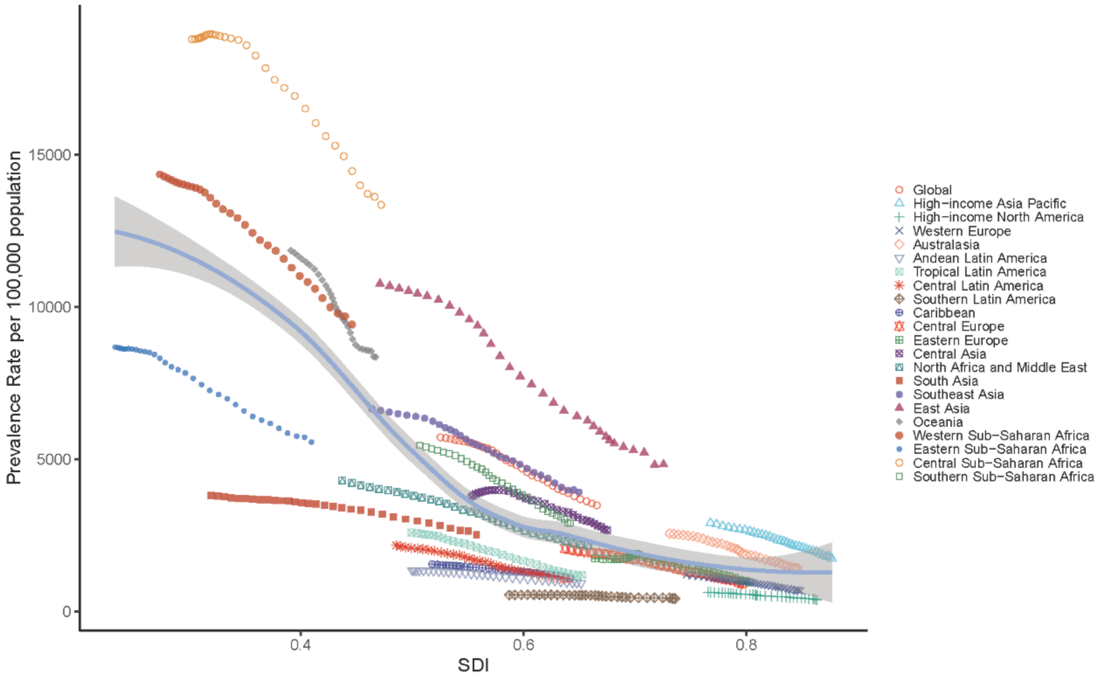

Fig.S19b Age-standardized Prevalence rates of cirrhosis due to hepatitis B, globally and for 21 GBD regions, by SDI, 1990–2021

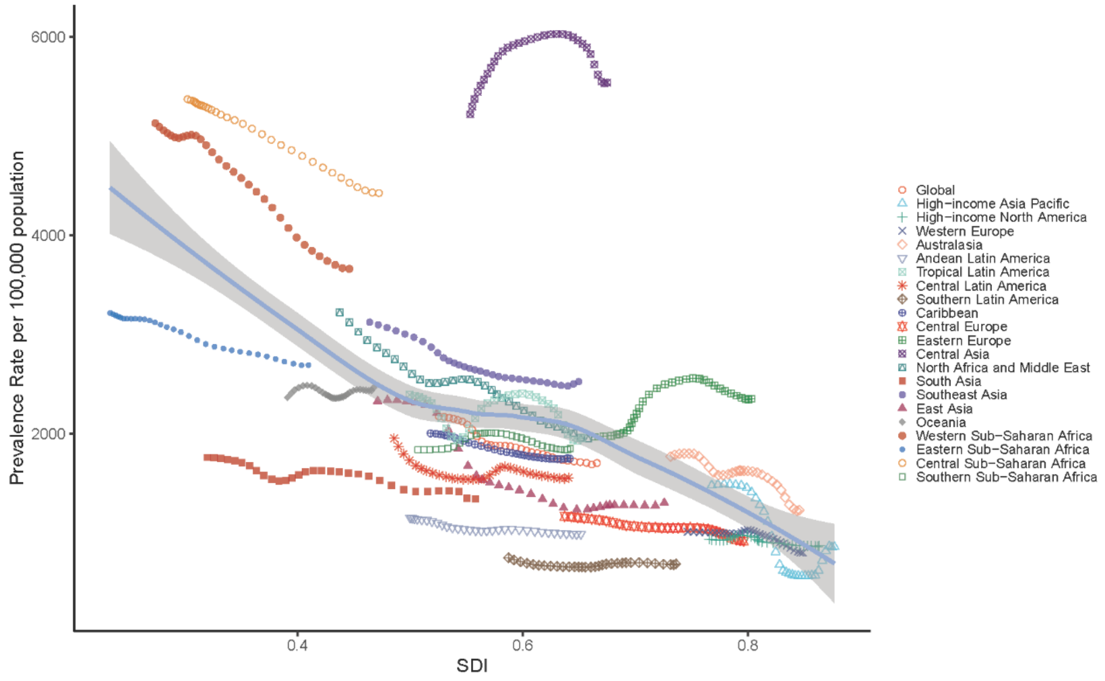

Fig.S19c Age-standardized Prevalence rates of cirrhosis due to hepatitis C, globally and for 21 GBD regions, by SDI, 1990–2021

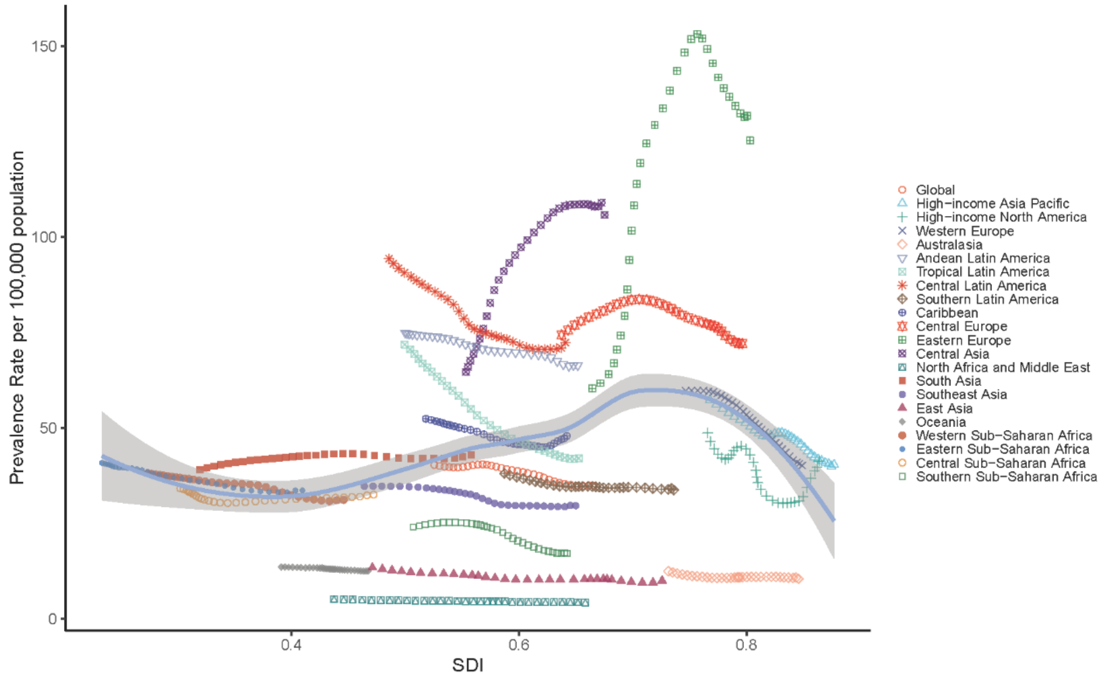

Fig.S19d Age-standardized Prevalence rates of cirrhosis due to alcohol, globally and for 21 GBD regions, by SDI, 1990–2021

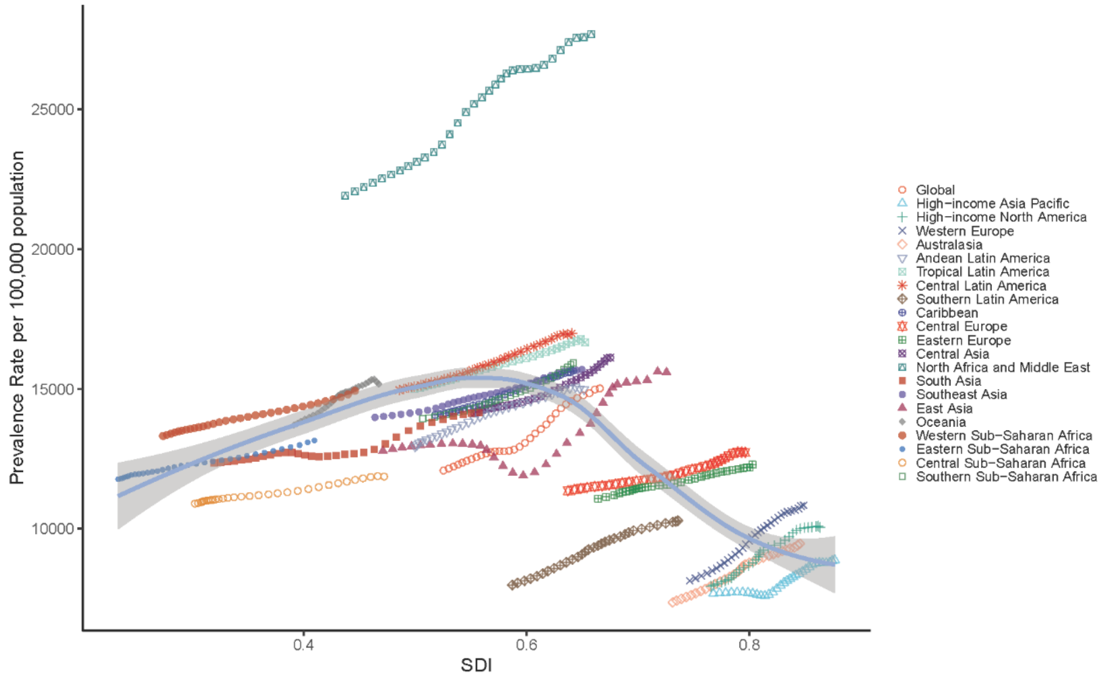

Fig.S19e Age-standardized Prevalence rates of cirrhosis due to NAFLD, globally and for 21 GBD regions, by SDI, 1990–2021

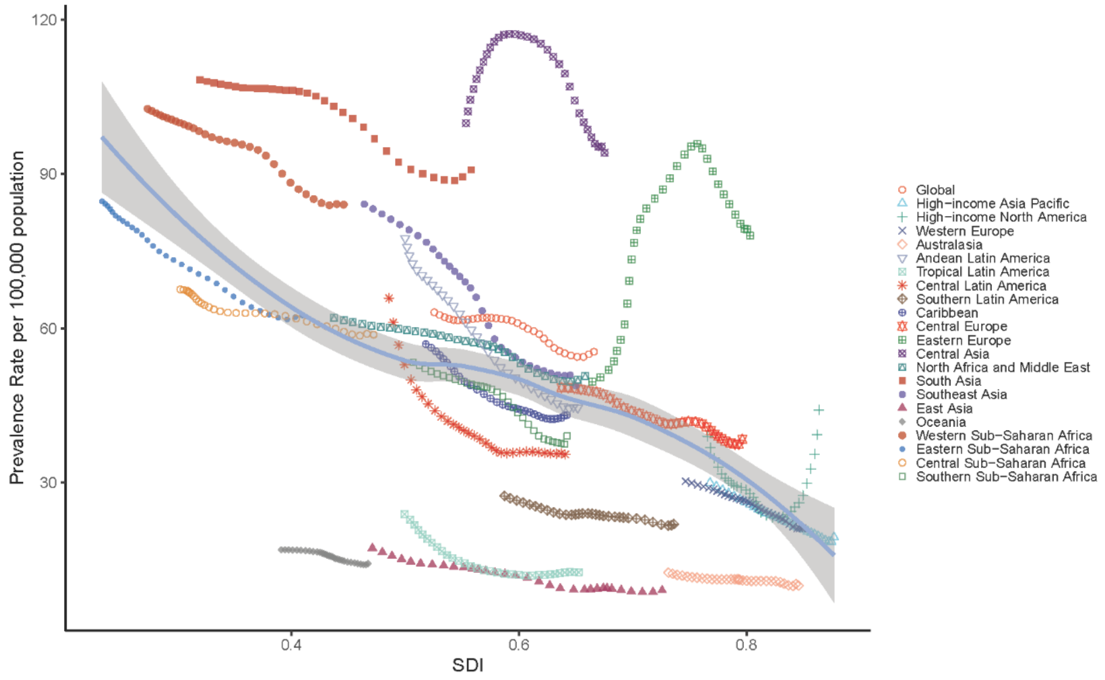

Fig.S19f Age-standardized Prevalence rates of cirrhosis due to other causes, globally and for 21 GBD regions, by SDI, 1990–2021

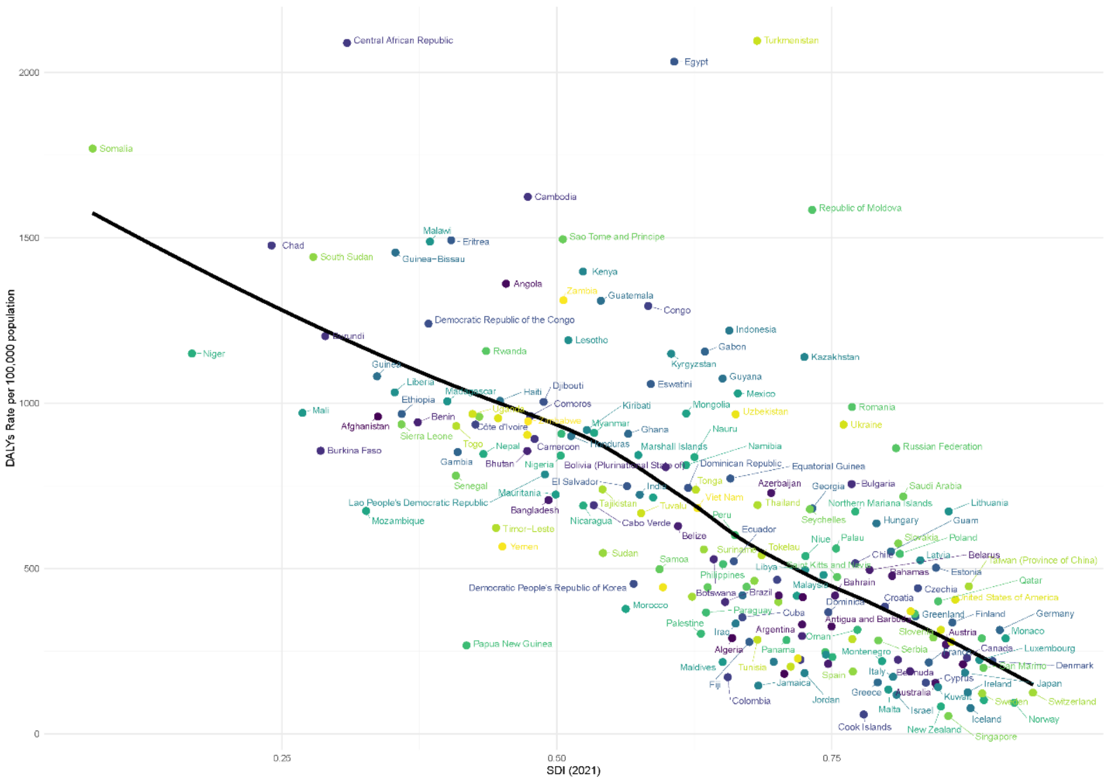

Fig.S20a Age-standardized DALYS rates of cirrhosis, globally and for 204 counties, by SDI, 1990–2021

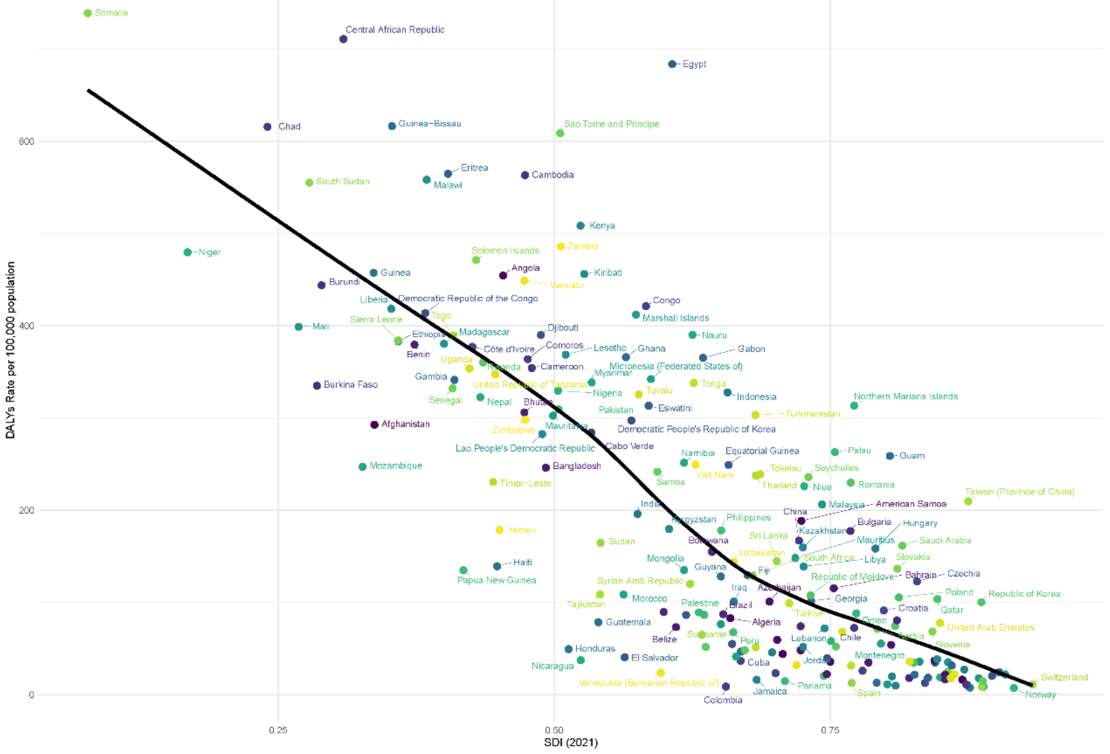

Fig.S20b Age-standardized DALYS rates of cirrhosis due to hepatitis B, globally and for 204 counties, by SDI, 1990–2021

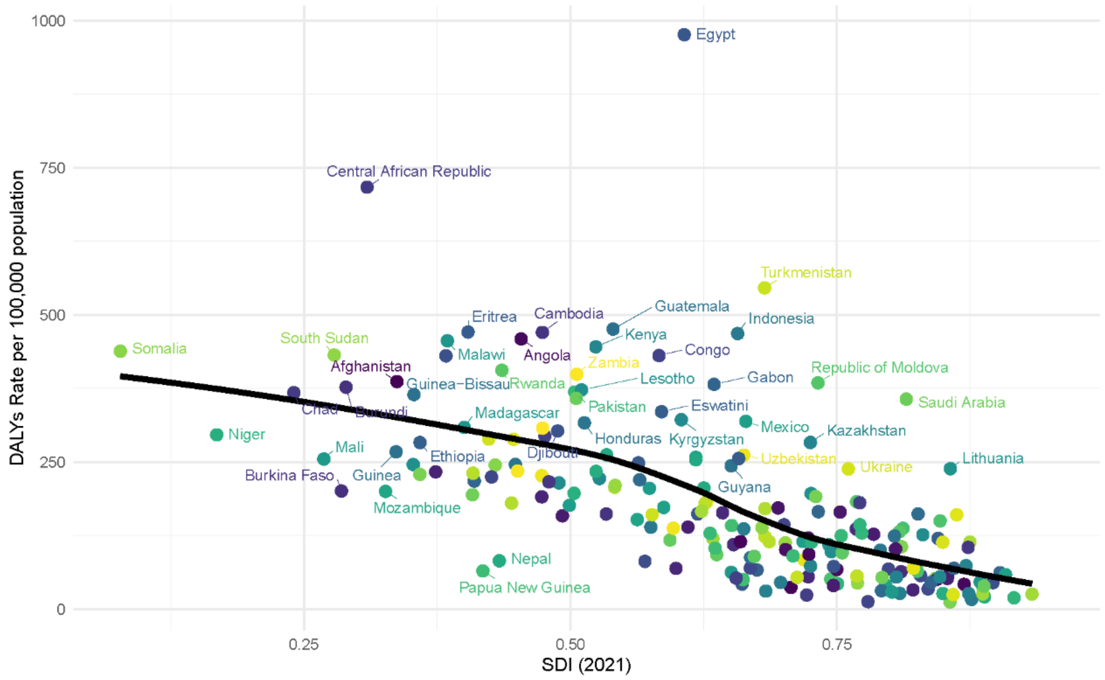

Fig.S20c Age-standardized DALYS rates of cirrhosis due to hepatitis C, globally and for 204 counties, by SDI, 1990–2021

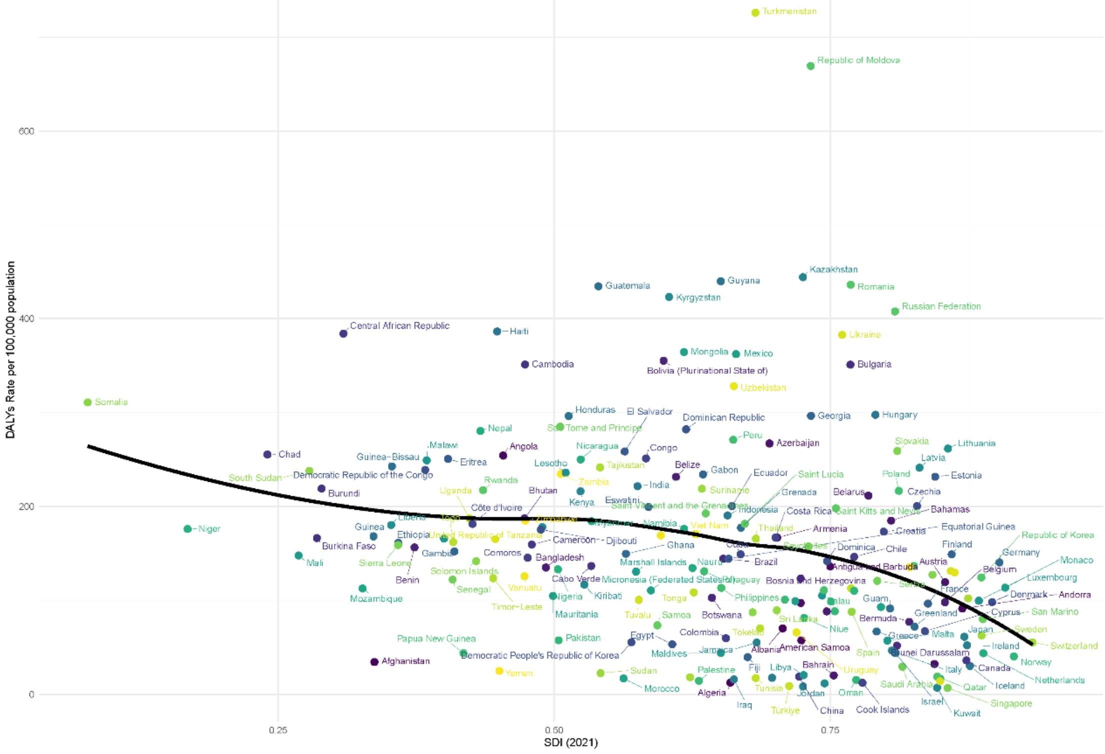

Fig.S20d Age-standardized DALYS rates of cirrhosis due to alcohol, globally and for 204 counties, by SDI, 1990–2021

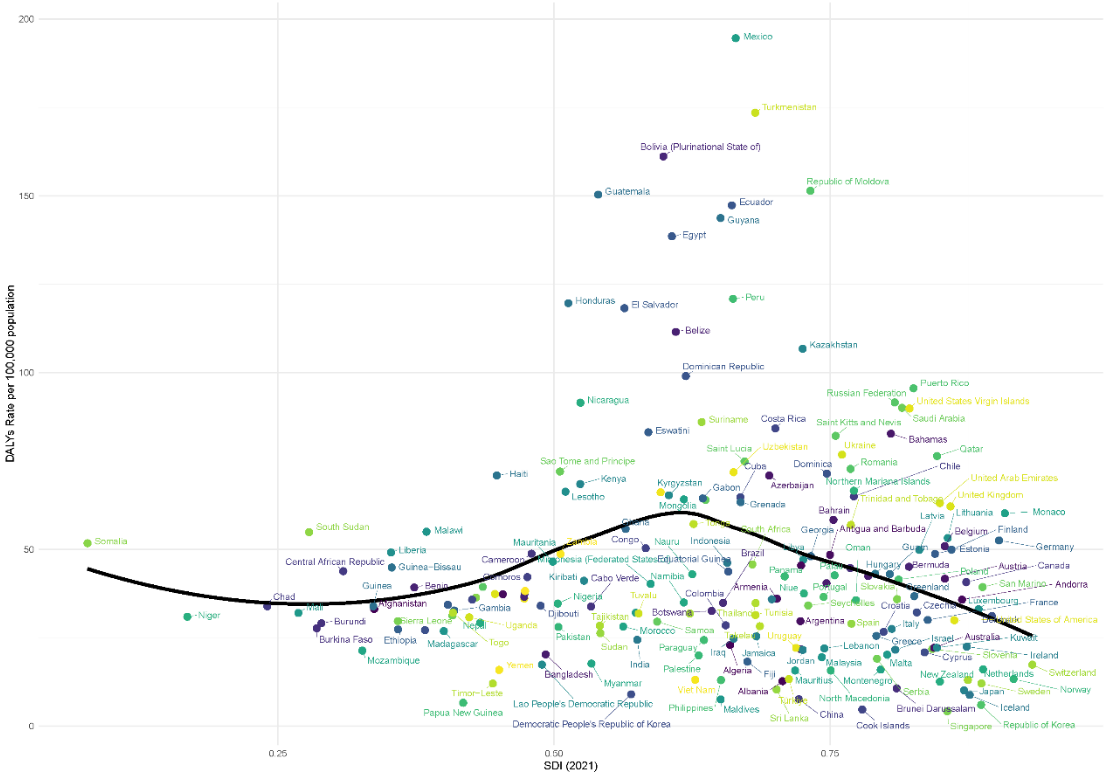

Fig.S20e Age-standardized DALYS rates of cirrhosis due to NAFLD, globally and for 204 counties, by SDI, 1990–2021

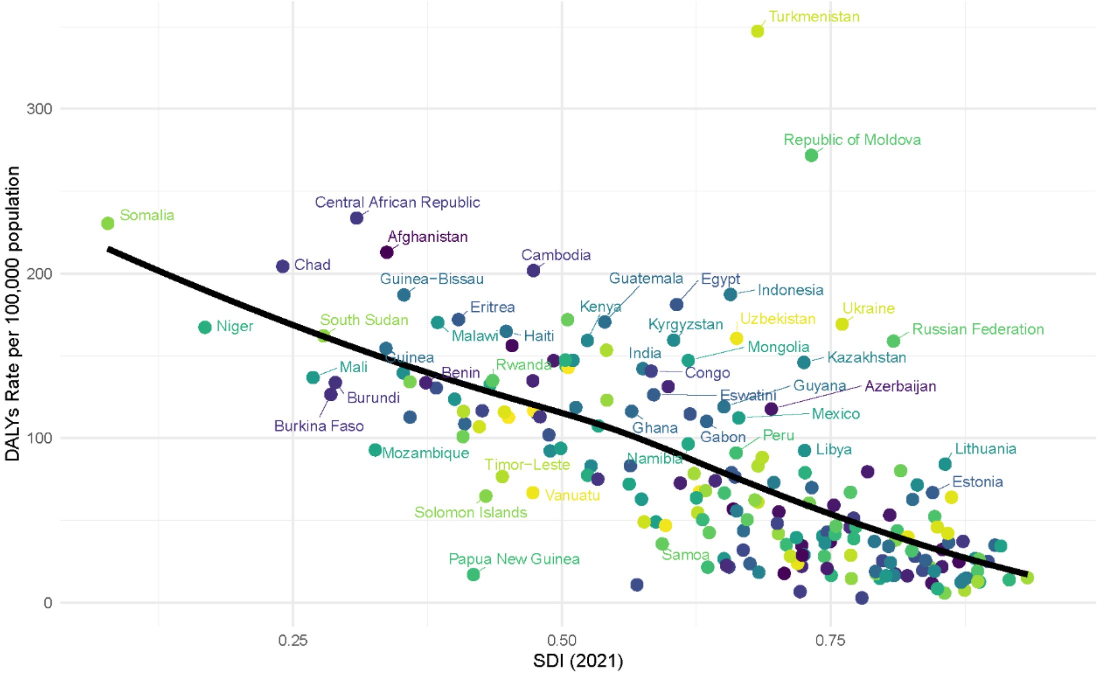

Fig.S20f Age-standardized DALYS rates of cirrhosis due to other causes, globally and for 204 counties, by SDI, 1990–2021

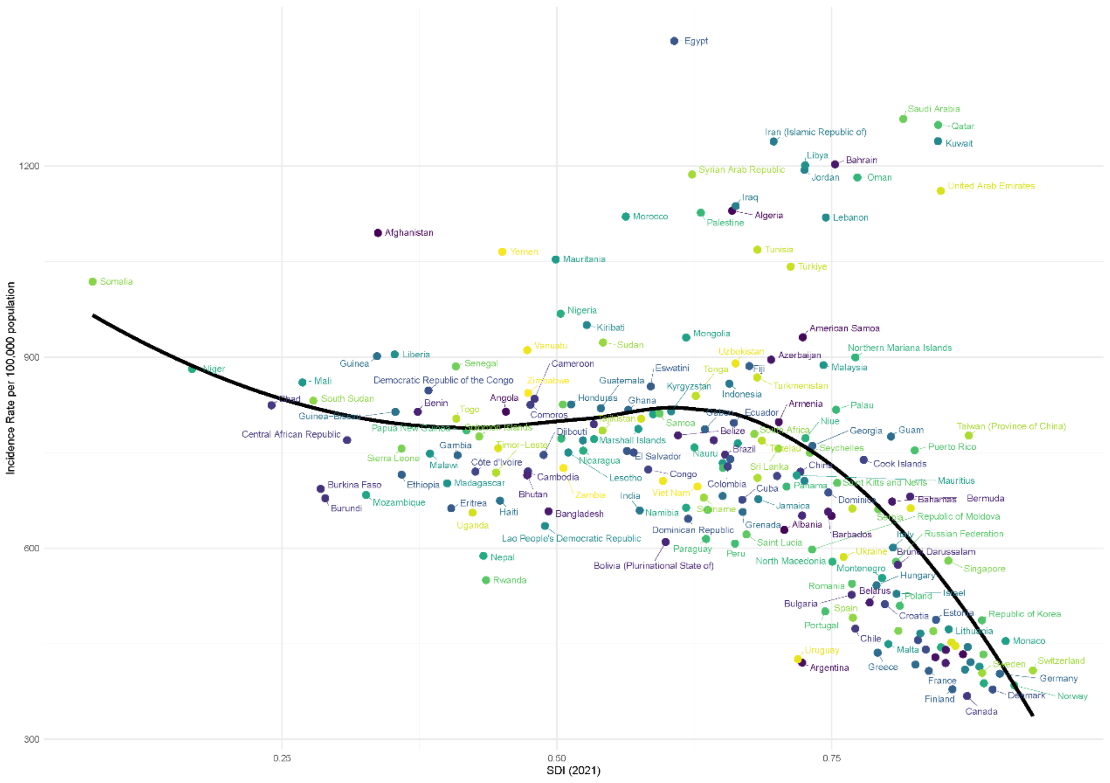

Fig.S21a Age-standardized Incidence rates of cirrhosis, globally and for 204 counties, by SDI, 1990–2021

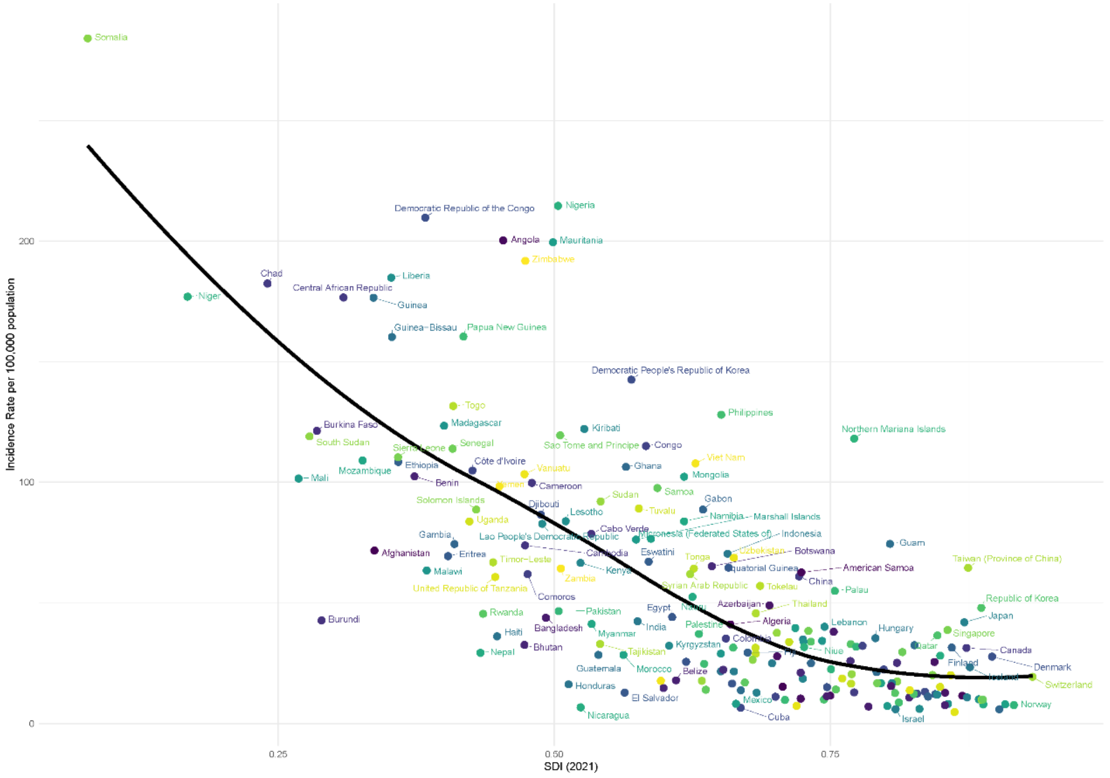

Fig.S21b Age-standardized Incidence rates of cirrhosis due to hepatitis B, globally and for 204 counties, by SDI, 1990–2021

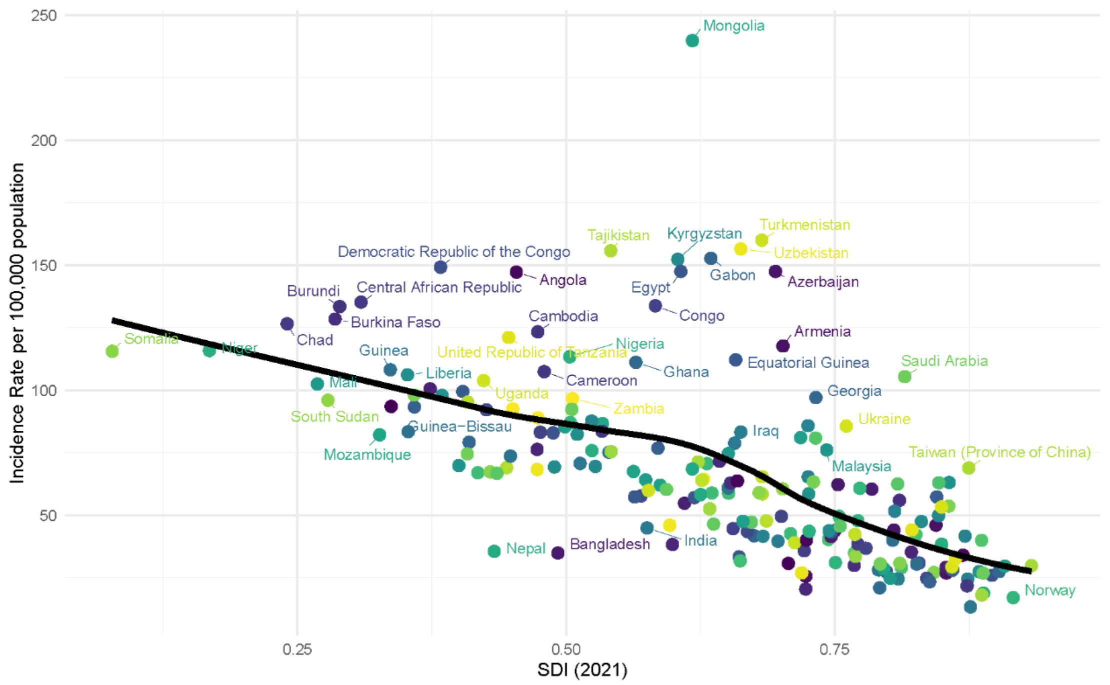

Fig.S21c Age-standardized Incidence rates of cirrhosis due to hepatitis C, globally and for 204 counties, by SDI, 1990–2021

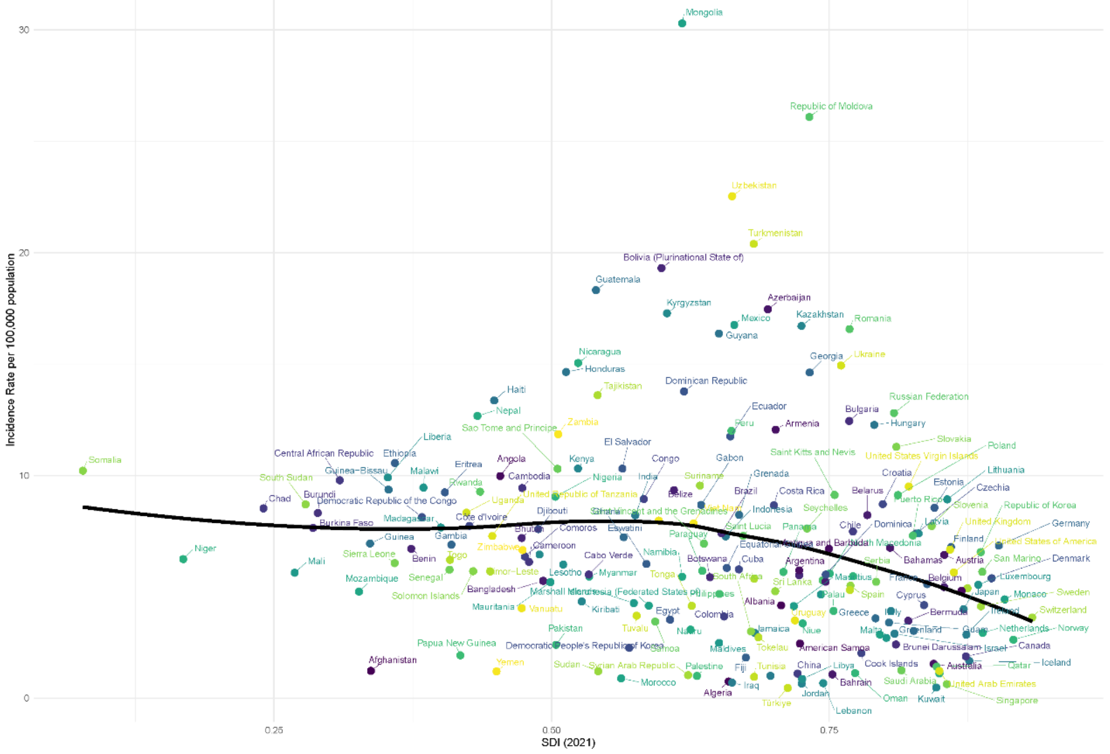

Fig.S21d Age-standardized Incidence rates of cirrhosis due to alcohol, globally and for 204 counties, by SDI, 1990–2021

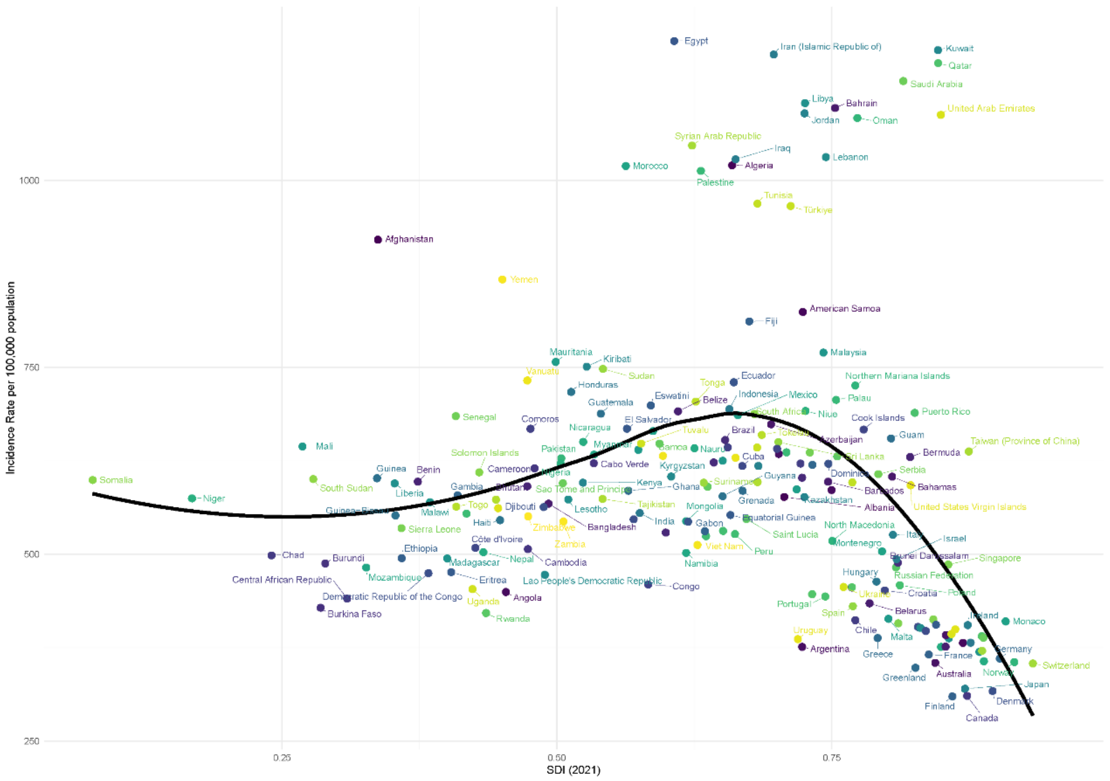

Fig.S21e Age-standardized Incidence rates of cirrhosis due to NAFLD, globally and for 204 counties, by SDI, 1990–2021

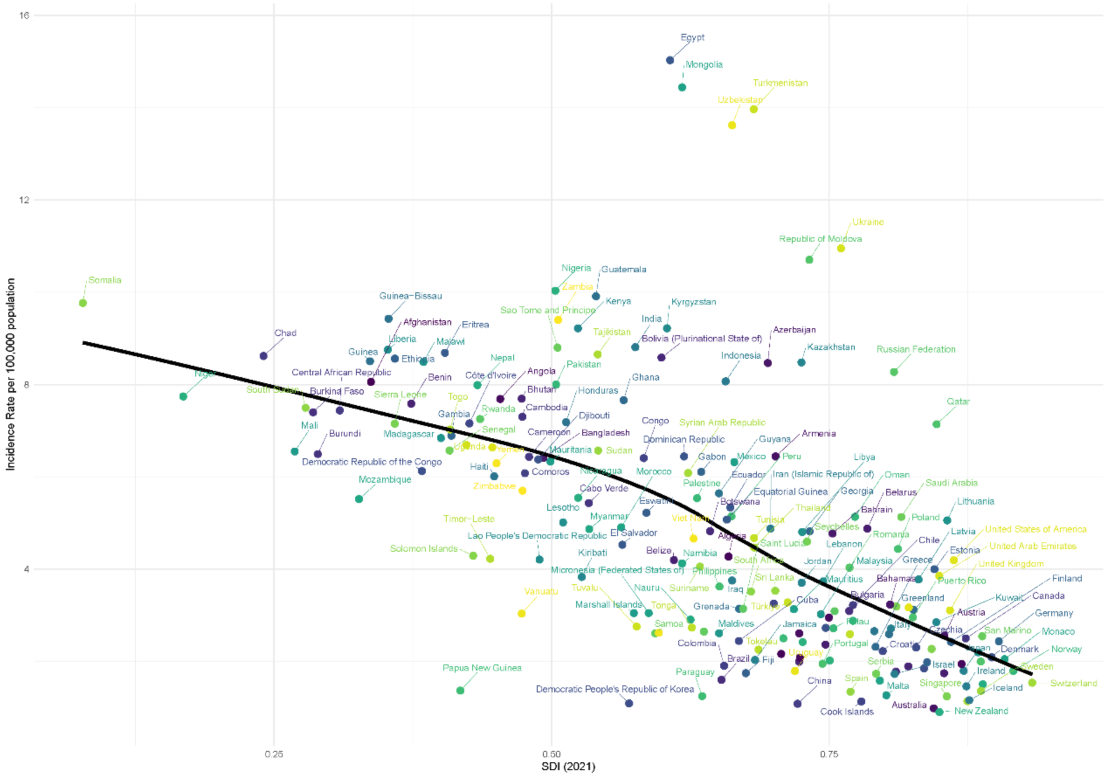

Fig.S21f Age-standardized Incidence rates of cirrhosis due to other causes, globally and for 204 counties, by SDI, 1990–2021

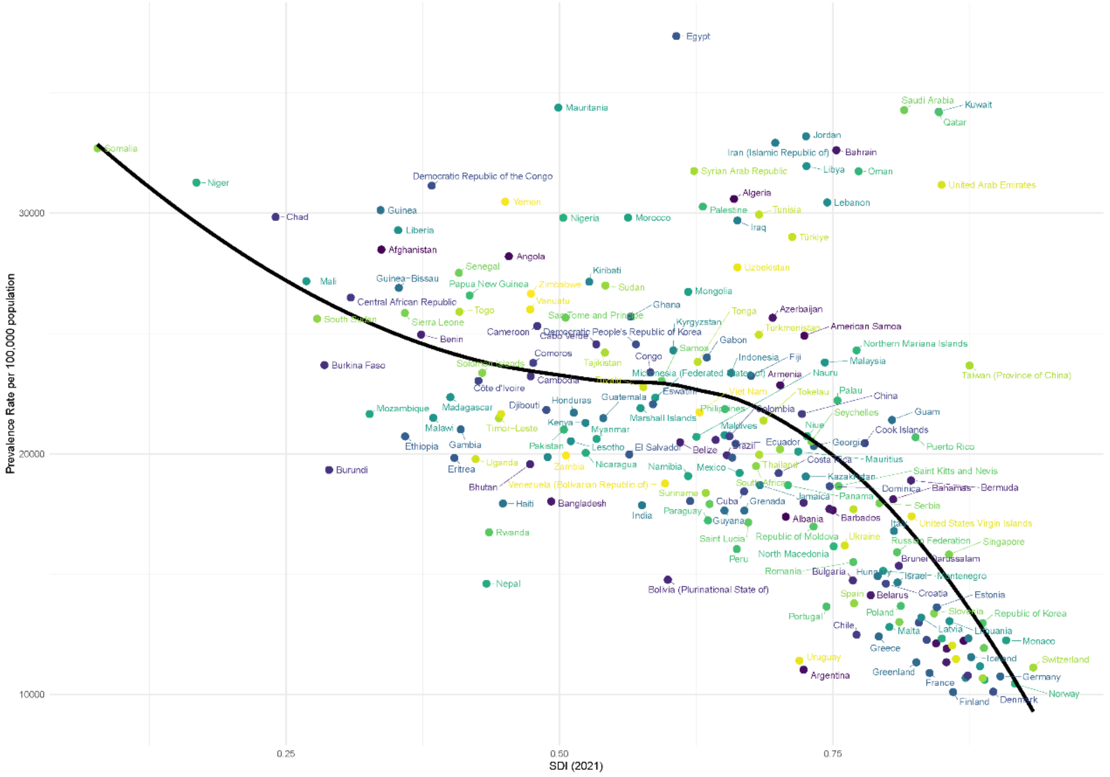

Fig.S22a Age-standardized Prevalence rates of cirrhosis, globally and for 204 counties, by SDI, 1990–2021

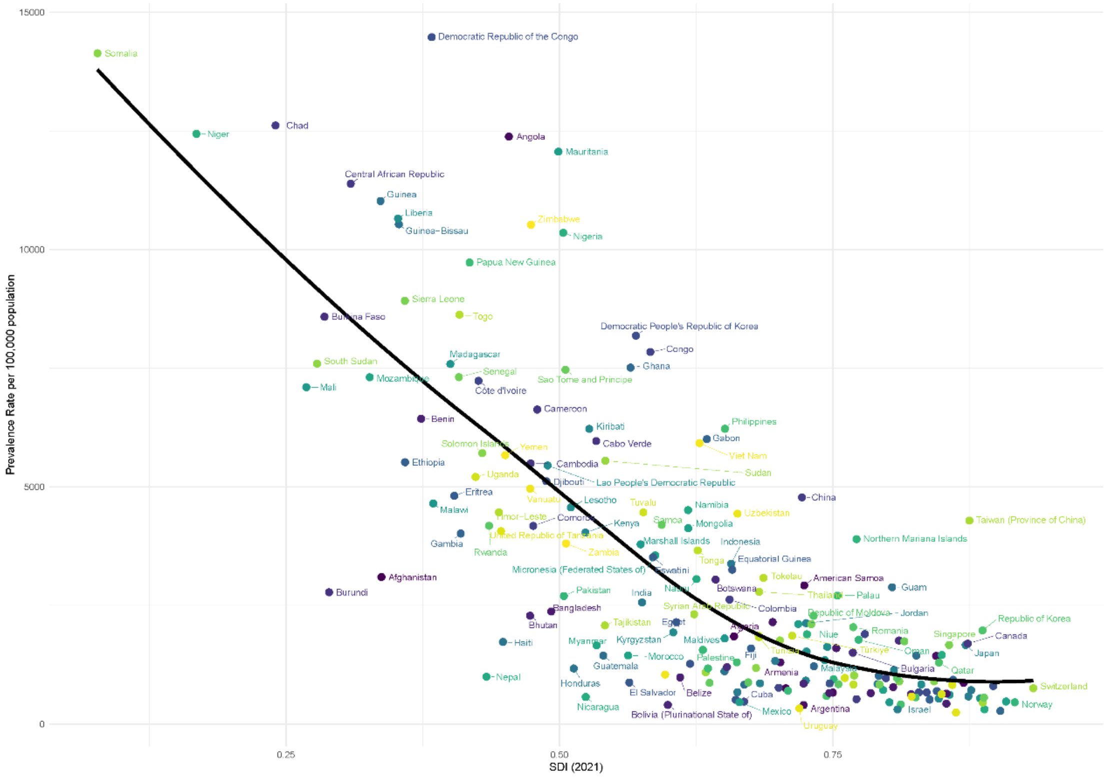

Fig.S22b Age-standardized Prevalence rates of cirrhosis due to hepatitis B, globally and for 204 counties, by SDI, 1990–2021

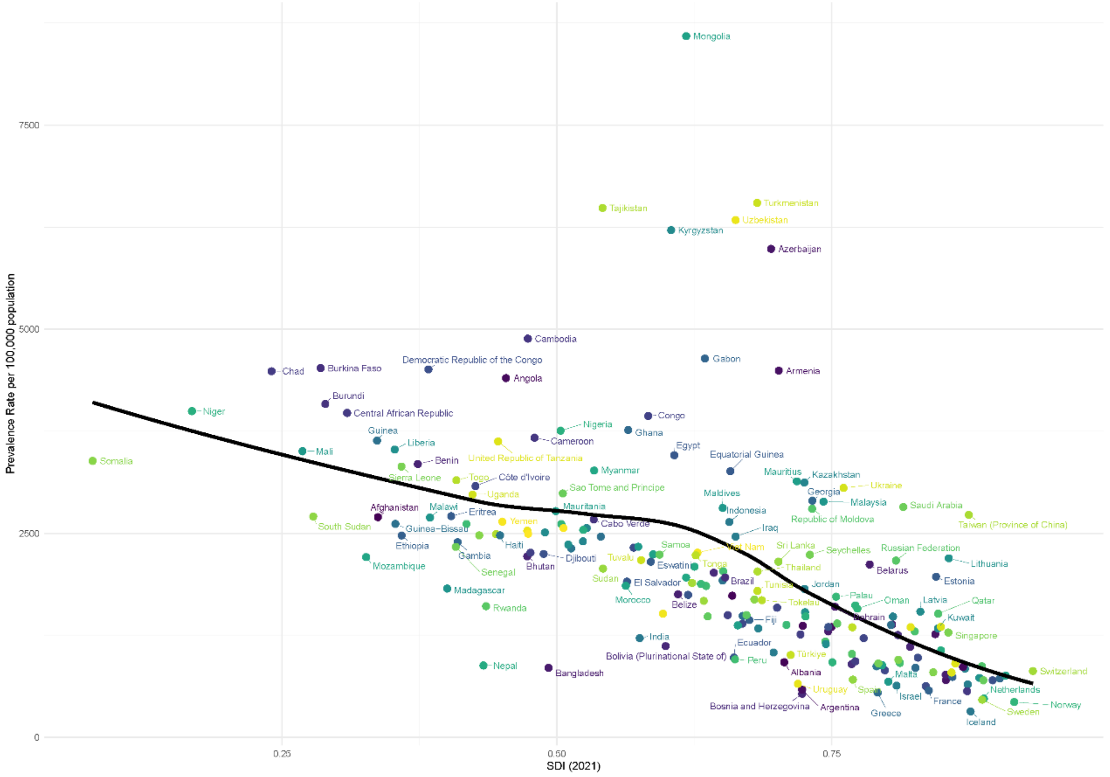

Fig.S22c Age-standardized Prevalence rates of cirrhosis due to hepatitis C, globally and for 204 counties, by SDI, 1990–2021

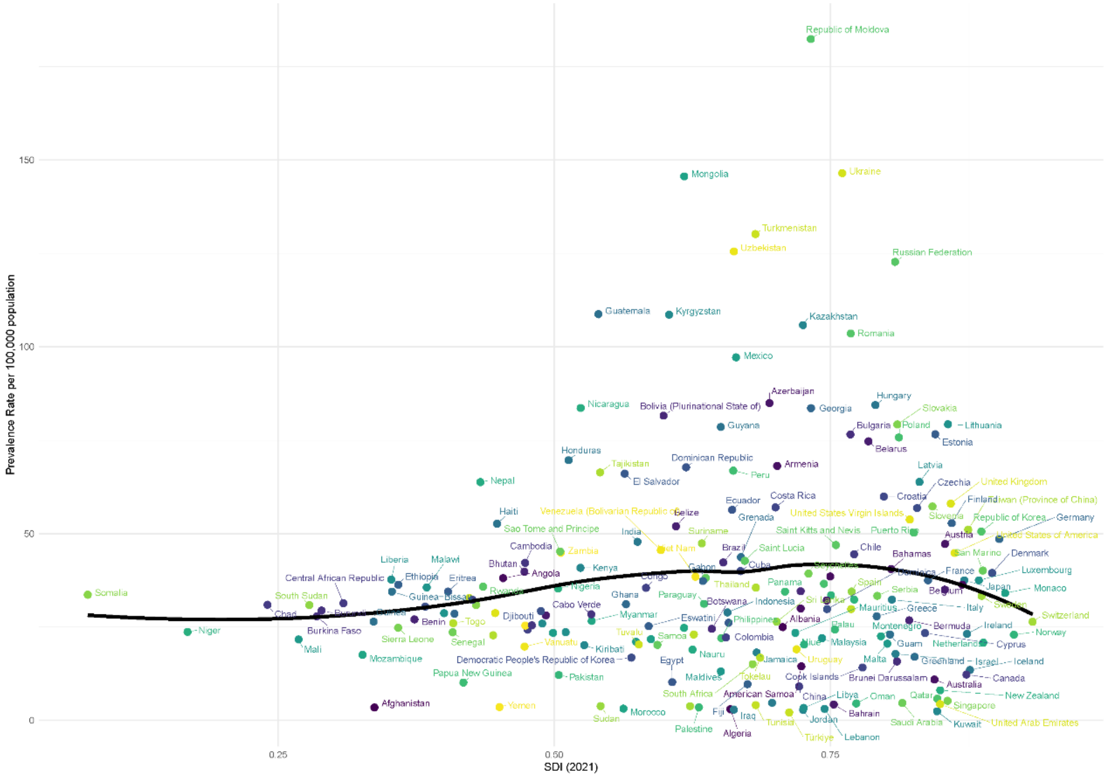

Fig.S22d Age-standardized Prevalence rates of cirrhosis due to alcohol, globally and for 204 counties, by SDI, 1990–2021

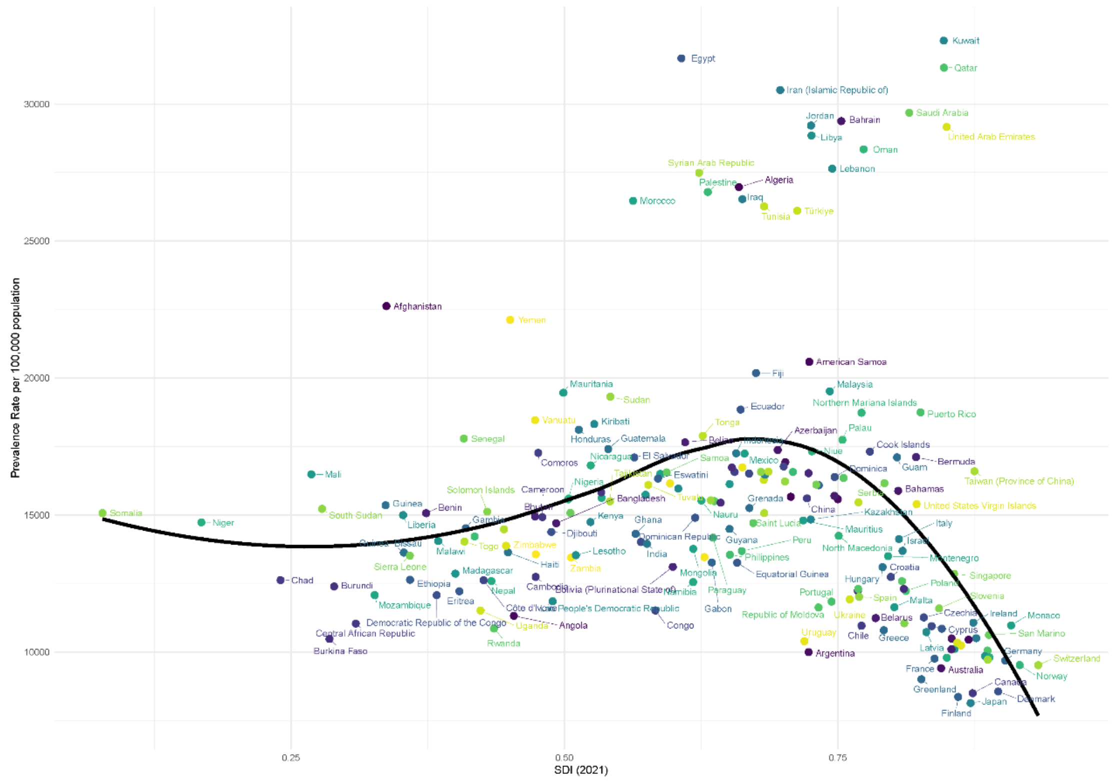

Fig.S22e Age-standardized Prevalence rates of cirrhosis due to NAFLD, globally and for 204 counties, by SDI, 1990–2021

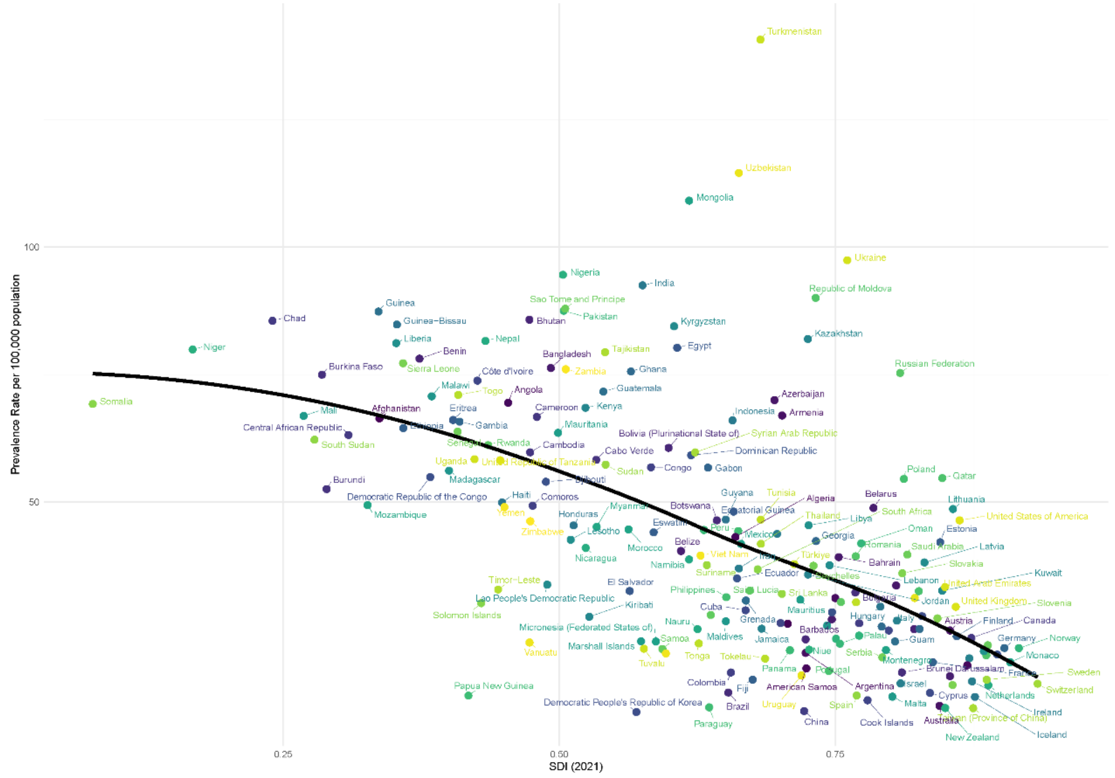

Fig.S22f Age-standardized Prevalence rates of cirrhosis due to other causes, globally and for 204 counties, by SDI, 1990–2021

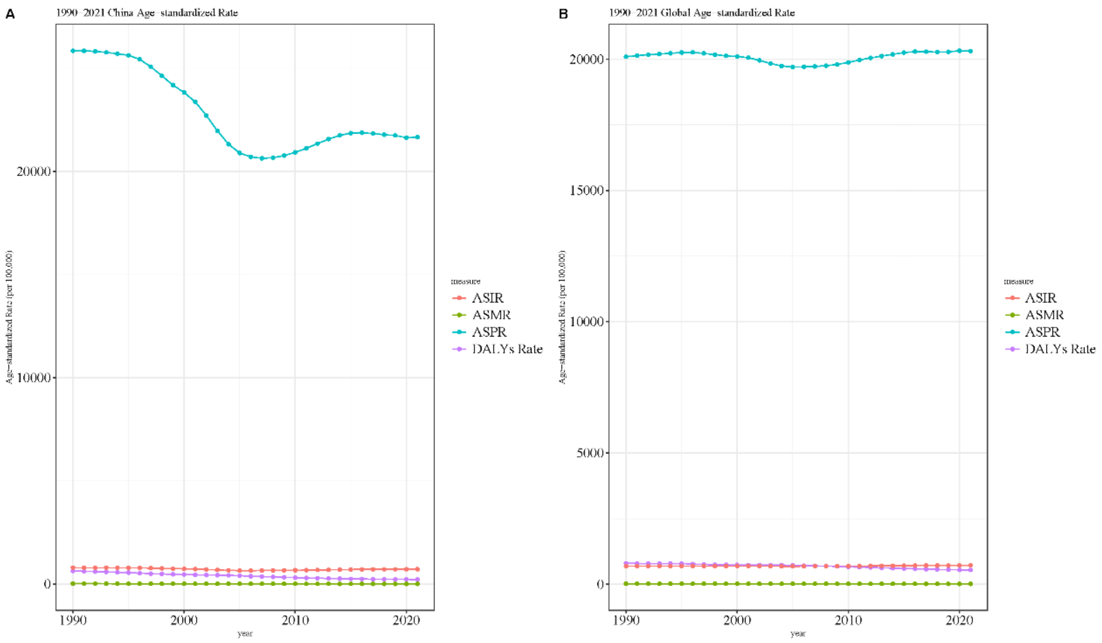

Fig.S23a Trend comparison of ASIR, ASPR, ASMR, and ASDR of cirrhosis in China and worldwide from 1990 to 2021

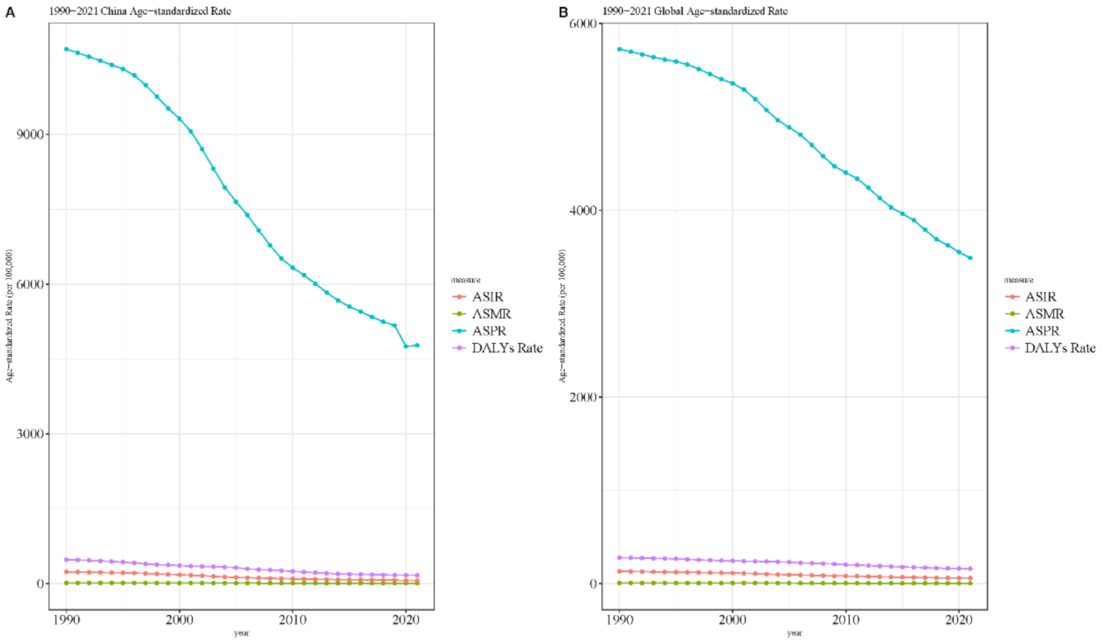

Fig.S23b Trend comparison of ASIR, ASPR, ASMR, and ASDR of cirrhosis due to hepatitis B in China and worldwide from 1990 to 2021

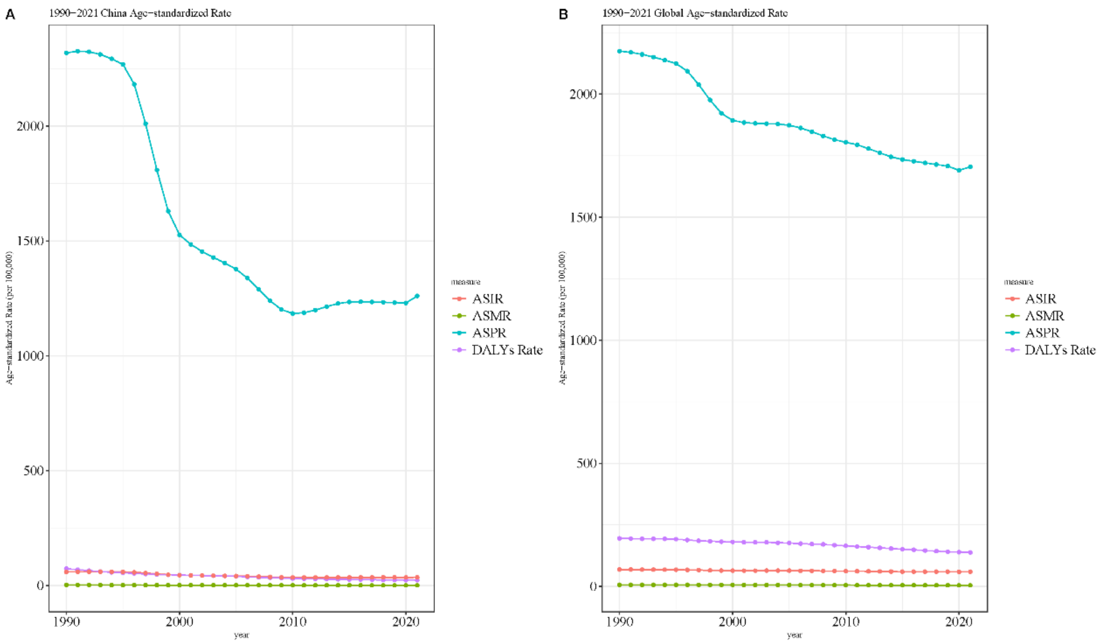

Fig.S23c Trend comparison of ASIR, ASPR, ASMR, and ASDR of cirrhosis due to hepatitis C in China and worldwide from 1990 to 2021

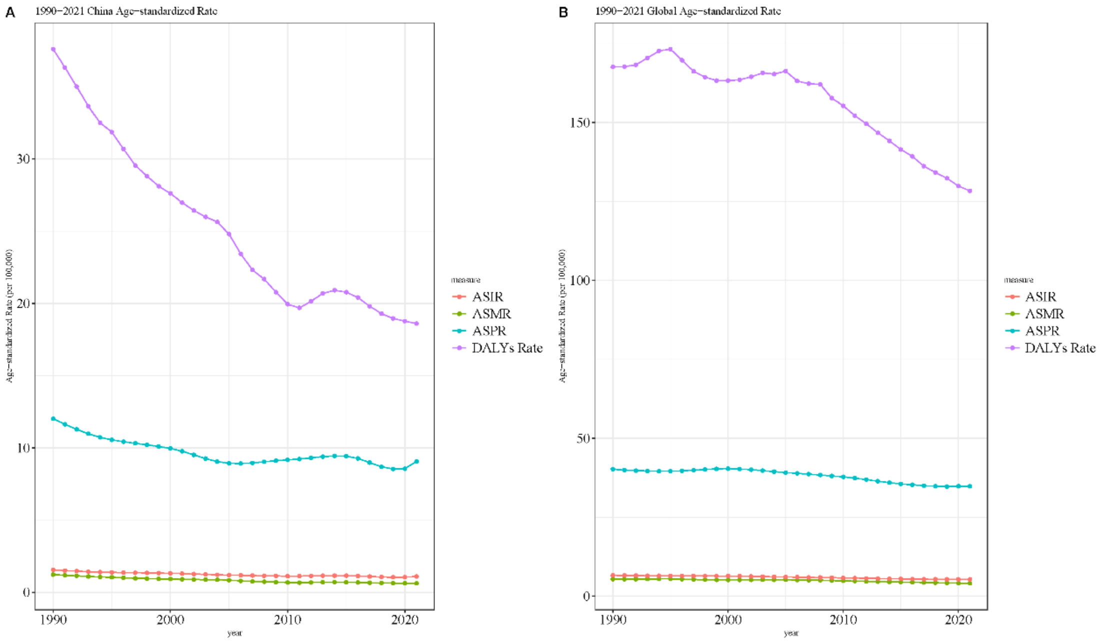

Fig.S23d Trend comparison of ASIR, ASPR, ASMR, and ASDR of cirrhosis due to alcohol in China and worldwide from 1990 to 2021

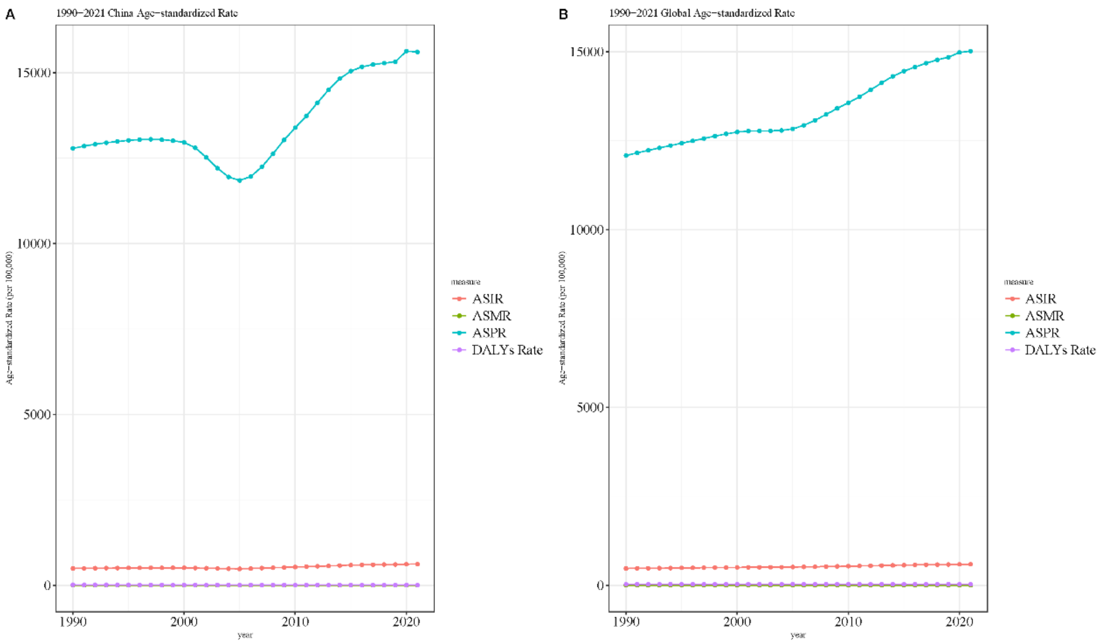

Fig.S23e Trend comparison of ASIR, ASPR, ASMR, and ASDR of cirrhosis due to NAFLD in China and worldwide from 1990 to 2021

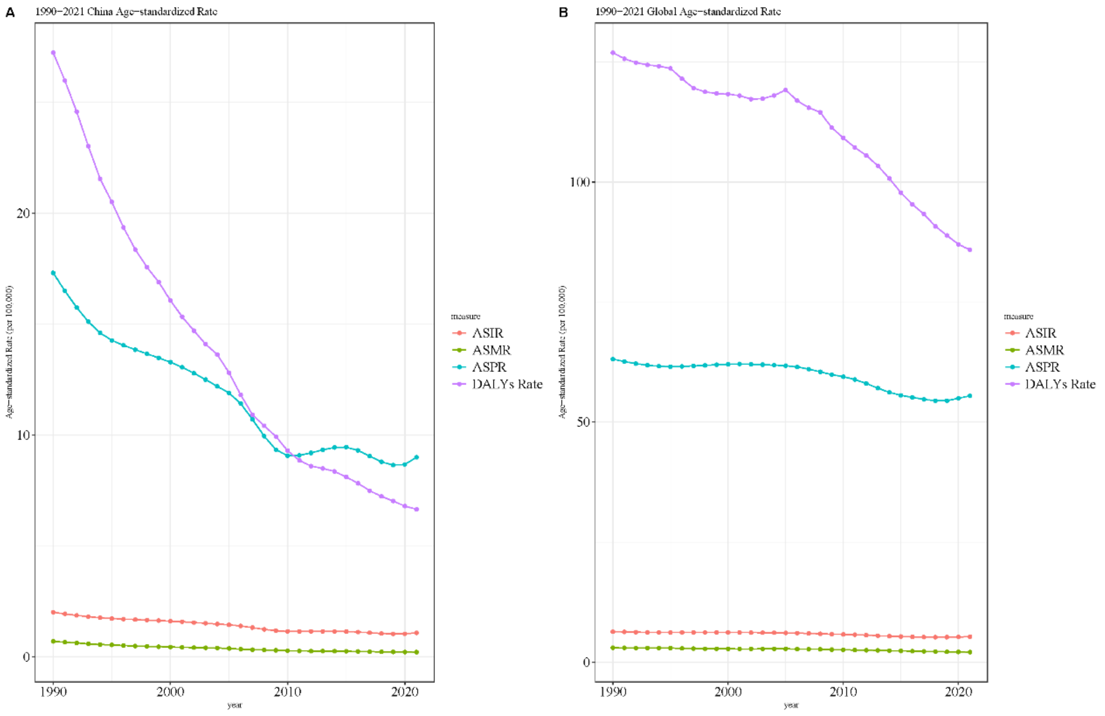

Fig.S23f Trend comparison of ASIR, ASPR, ASMR, and ASDR of cirrhosis due to other causes in China and worldwide from 1990 to 2021
